# Synthesis and *In Vitro* Antileishmanial Efficacy of Novel Ethylene Glycol Analogues of Benzothiadiazine‐1,1‐dioxide

**DOI:** 10.1002/cbdv.202402059

**Published:** 2024-10-31

**Authors:** Nadine Henning, Christina Kannigadu, Janine Aucamp, Helena D. Janse van Rensburg, Frank van der Kooy, David D. N'Da

**Affiliations:** ^1^ Centre of Excellence for Pharmaceutical Sciences (Pharmacen) North-West University Potchefstroom 2520 South Africa

**Keywords:** *Leishmania*, Promastigote, Amastigote, Carbonic anhydrase, Benzothiadiazine, Ethylene glycol

## Abstract

Leishmaniasis is a vector‐borne, parasitic disease affecting millions of people and animals worldwide. Current therapeutic options have proven to be ineffective in both treating the disease and preventing its spread. As a result, new drugs must be developed to effectively combat this disease. In this study, a series of 14 ethylene glycol analogues of benzothiadiazine‐1,1‐dioxide were synthesised to investigate their antileishmanial potential and cytotoxicity. Analogue **9**, 2‐(2‐phenoxyethyl)‐2*H*‐benzo[*e*][1,2,4]thiadiazine‐1,1‐dioxide, was identified as the most inhibitory compound as it was observed to moderately inhibit the growth of *L. major* (IC_50_ 103 μM) and *L. donovani* (IC_50_ 153 μM) promastigotes. However, in general, the series presented with low biological activity, which may be attributed to reduced target affinity and/or undesired cell culture protein binding.

## Graphical abstract

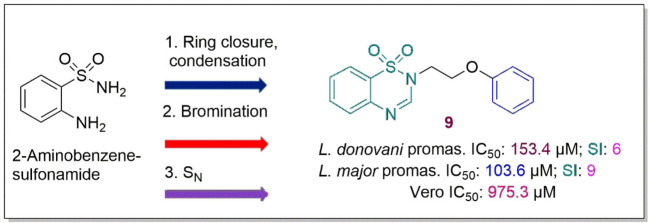

## 1 Introduction

Leishmaniasis is a parasitic disease that is caused by protozoa of the genus *Leishmania*.^[1]^ There are approximately 20 human‐infectious species of *Leishmania*, which are transmitted to the mammalian host *via* the bite of an infected *Phlebotomus* sandfly.[^1^, ^2^] Once infection has occurred, the disease can manifest itself in three main clinical forms: visceral leishmaniasis (VL), which is a dangerous systemic infection that affects the internal organs; cutaneous leishmaniasis (CL), which can be identified by skin lesions and ulceration; mucocutaneous leishmaniasis (MCL), where lesions are present on the nasopharyngeal and oral mucous membranes.[^3^, ^4^]

Leishmaniasis is found in over 90 nations in Southern Europe, the tropics and subtropics of Africa, Asia, the Middle East, the Mediterranean basin, and the Americas, the majority of which are developing countries.[^1^, ^5^] Despite having such a notable presence, this disease is still classified as a neglected tropical disease (NTD) due to a lack of financial investment in disease management strategies.^[6]^ As of 2023, the World Health Organization (WHO) estimated that 700 000 to 1 million new cases occur globally each year, with 350 million people living in endemic areas that are at risk of developing leishmaniasis.^[1]^

There are currently no vaccines or prophylactic drugs available for humans to prevent *Leishmania* infection, hence the primary treatment modality for the management of leishmaniasis remains chemotherapy.^[5]^ Current clinical antileishmanial drugs include the pentavalent antimonials, sodium stibogluconate and meglumine antimoniate, pentamidine, amphotericin B, miltefosine, paromomycin and the azole antibiotics, itraconazole, fluconazole and ketoconazole.[^5^, ^7^] Most of these drugs are toxic, expensive, and require nosocomial settings to be administered,[^5^, ^8^] which limit their use in leishmaniasis endemic countries.

Furthermore, global parasitic resistance to antileishmanial therapies is on the rise with resistance already evident against the pentavalent antimonial drugs and miltefosine.[^8^, ^9^] Combination drug therapy has accordingly been utilised to minimise drug resistance. However, due to drug misuse, overuse and the inherent genetic variety displayed by *Leishmania* spp., this remains an ongoing challenge.[^9^, ^10^] With these limitations taken into consideration, effectively combatting this NTD relies on the development of treatments that are safe, cost‐effective, and less vulnerable to the development of resistance. However, the similarities that can be drawn between eukaryotic protozoan cells and human cells pose a substantial challenge in the realm of antileishmanial treatment research.^[11]^ To reduce toxicity and undesired side effects, treatment must be as specific as possible towards the infective protozoa.^[12]^

A promising drug target that is under investigation is the carbonic anhydrase (CA) enzyme.[^11^, ^13^] To date, seven families of CA have been described in literature, namely *α*‐, *β*‐, *γ*‐, *δ*‐, *ζ*‐, *η*‐ and *θ*‐CA.^[11]^ CA present in humans is of the *α*‐family, whereas protozoa encode for both *α*‐ and *β*‐CA.^[14]^ As a result, the *β*‐CA not found in humans serves as a suitable pharmacological target for overcoming the problem of drug selectivity.^[15]^

Furthermore, *Leishmania* cells maintain near‐neutral intracellular pH and proliferate in such acidic conditions by relying on a cytosolic and a cell surface carbonic anhydrase, LmCA1 and LmCA2, respectively.^[16]^ Thus, targeting carbonic anhydrases from *Trypanosoma cruzi* and *Leishmania* species has emerged as a therapeutic strategy to obtain new antiprotozoal drugs.^[17]^ Notably, benzoxaborole derivatives have shown to inhibit carbonic anhydrases from *Trypanosoma cruzi* and *Leishmania donovani chagasi*.^[18]^ Likewise, *N*‐nitrosulfonamide demonstrates important inhibition of CA hence has been identified as a promising chemotype for targeting chagas disease and leishmaniasis.

The primary functional group in the CA inhibitor drug class is sulfonamide, which consists of a sulfur atom coupled to two oxygens and an amine.^[19]^ The benzothiadiazine structure has been previously studied as a scaffold for the synthesis of new antibacterial drugs.^[20]^ It is also found in clinically used drugs such as the thiazide diuretic hydrochlorothiazide (Hexazide), as well as diazoxide, which is used to manage hypoglycaemia in patients with excessive insulin production.[^21^, ^22^] Benzothiadiazines approved as diuretic agents (e. g., saluron, naturetin and renese) for the treatment of edema and hypertension, either in monotherapy or in combination to enhance the effectiveness of other antihypertensive drugs, especially in the more severe forms of hypertension,^[23]^ belong to the category of sulfonamides that inhibit carbonic anhydrase (CA).^[24]^

**Figure 1:**
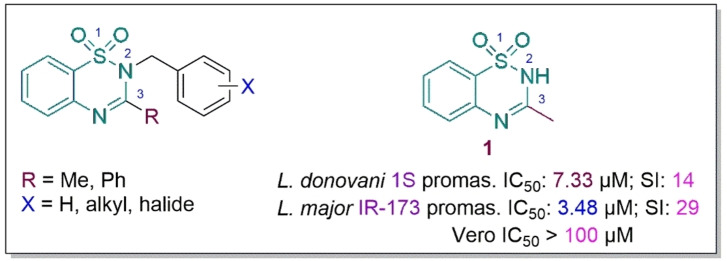
General structure of *N*‐benzyl analogues of 1,2,4‐benzothiadiazine‐1,1‐dioxide and *Leishmania* antipromastigote hit 1 as reported by Mangwegape et al.^[25]^

As part of our ongoing program aimed at the discovery of new, safe and affordable antileishmanial therapeutics, we previously investigated the antileishmanial efficacy of 3‐methyl‐*N*‐benzyl‐substituted derivatives of 1,2,4‐benzothiadiazine‐1,1‐dioxide.^[25]^ These compounds showed promising and selective antiprotozoan activity as they were found to be non‐toxic to mammalian cells. However, when compared to the parent 1,2,4‐benzothiadiazine‐1,1‐dioxide **1**, which had an IC_50_ <10 μM against *L. donovani* and *L. major* promastigotes, these analogues did not show a substantial increase in antileishmanial activity (Figure 1).^[25]^

Furthermore, **1** displayed sub‐micromolar activity against *L. donovani* strain 1S amastigote as well as a preference for amastigote over the promastigote form. This prompted further exploration of the 1,2,4‐benzothiadiazine‐1,1‐dioxide moiety as a potential antileishmanial synthon hence the current study.

Another aim of the previous study was to assess whether the enhancement of lipophilicity through the anchorage of the benzyl moiety to 1,2,4‐benzothiadiazine‐1,1‐dioxide synthon would result in improved antileishmanial activity. This did not appear to be the case.^[25]^ In the current study, we aimed to assess the impact of amphiphilicity on the antileishmanial activity. To this end, oligomeric ethylene glycol moieties were appended to the unsubstituted 1,2,4‐benzothiadiazine‐1,1‐dioxide synthon. Ethylene glycol chains were chosen based on their reported amphiphilic properties.^[26]^ In this study, terminal alkyl/aryl substituents as well as chain length of the ethylene oxide, were also considered to assess potential structure‐activity relationship (SAR), if any, in the series.

**Figure 2:**
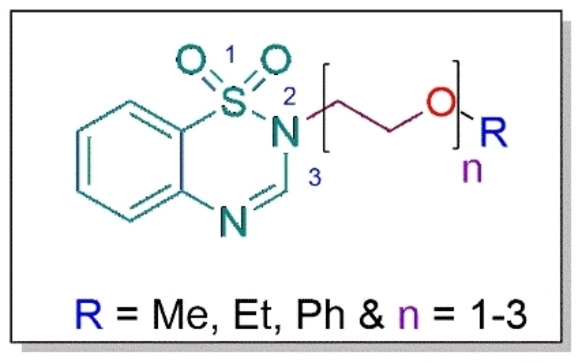
General structure of target compounds.

Furthermore, a propargyl moiety was appended to the 1,2,4‐benzothiadiazine‐1,1‐dioxide core to evaluate its impact on the biological activity, based on literature studies that report this moiety to greatly boost antileishmanial efficacy.^[27]^ The general structure of the target compounds is depicted in Figure 2. In addition, the novel analogues were tested on Vero cell line to assess their safety. We herein report the synthesis and biological activities of these derivatives.

## 2 Results and Discussion

### 2.1 Chemistry

**Scheme 1:**
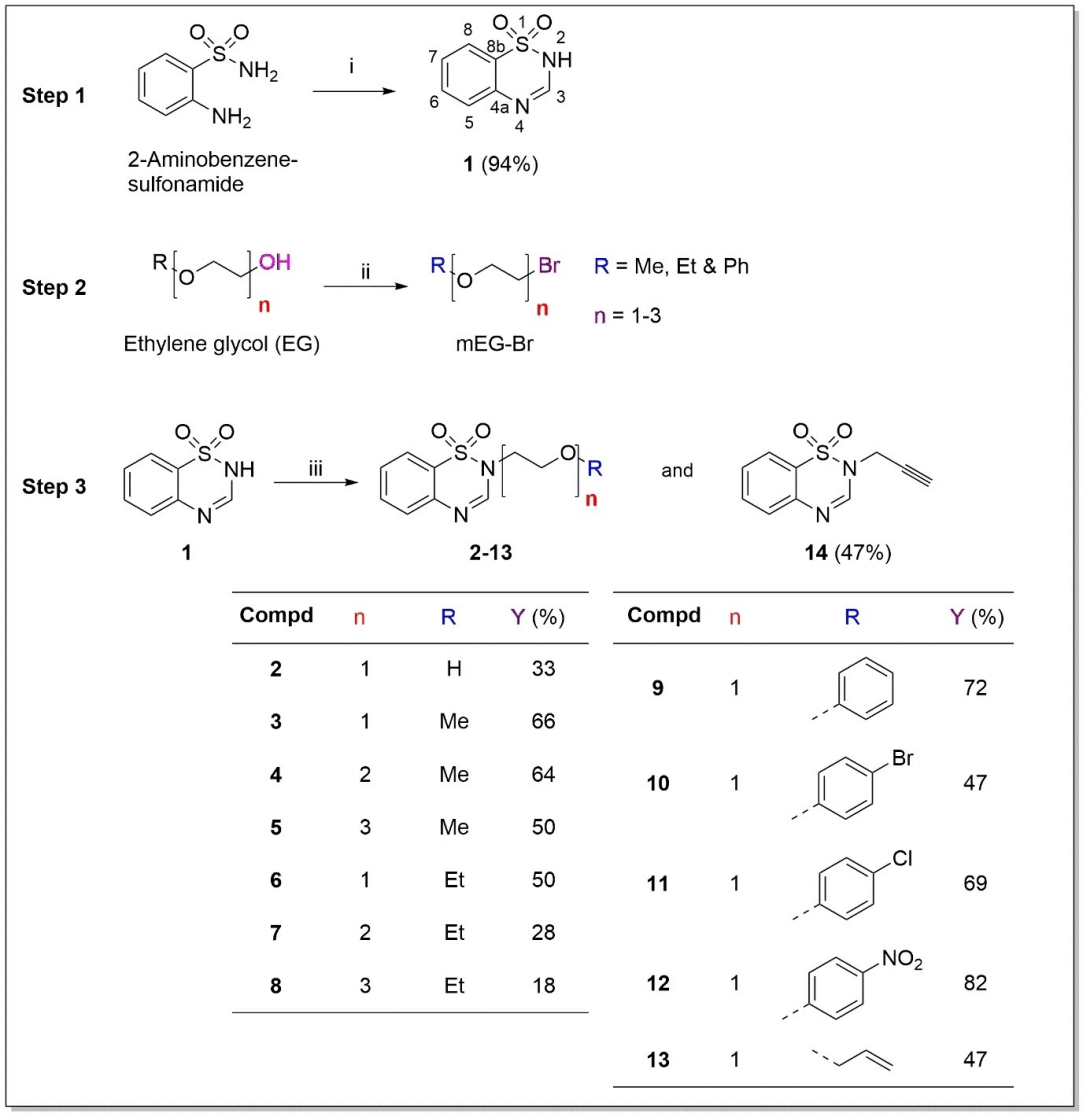
Three‐step synthesis of target EG appended benzothiadiazine‐1,1‐dioxide analogues. *Reagents and conditions*: (i) Triethyl orthoformate (7 equiv.), rt, 12 h (ring closure & condensation), (ii) PBr_3_ (1/3 equiv.), dry chloroform, rt, 12 h (bromination), (iii) mEG−Br (1.2 equiv.), DMF (4 ml), rt, 12 h (S_N_).

A series of 1,2,4‐benzothiadiazine‐1,1‐dioxide analogues bearing ethylene glycol (EG) oligomeric units at N‐2 position were synthesised using a three‐step process as illustrated in Scheme 1. First, the benzothiadiazine‐1,1‐dioxide scaffold was obtained through Schiff condensation of commercial 2‐aminobenzenesulfonamide with triethylorthoformate, acting as both reagent and solvent. This gave rise to a Schiff base, which allowed for ring closure and the subsequent formation of 2*H*‐benzo[*e*][1,2,4]thiadiazine‐1,1‐dioxide (**1**) in an excellent yield of 94 % (Scheme 1; Step 1). The adopted synthetic route had previously been reported.^[25]^ However, the reaction was herein reduced to 12 hours instead of 24–36 hours with no significant difference in the yield.

Second, a series of brominated ethylene glycol intermediates were formed through bromination of the alcohol group attached to the α‐carbon using phosphorous tribromide (PBr_3_) (Scheme 1; Step 2).

In the third step, the N‐2 atom of the benzothiadiazine‐1,1‐dioxide scaffold **1** was deprotonated using potassium carbonate (K_2_CO_3_). This was followed *in situ* by nucleophilic substitution (S_N_) with the various brominated ethylene glycol intermediates to afford the targeted benzothiadiazine‐1,1‐dioxide derivatives (**2–14**) in poor to excellent yields (17–81 %) after filtration and purification by column chromatography (DCM:MeOH, 19 : 1) (Scheme 1; Step 3).

Routine molecular analysis techniques including, nuclear magnetic resonance (NMR), high‐resolution mass spectroscopy (HRMS) and infra‐red (IR), were used to confirm the structures of compounds **1–14** (Supplementary Information). The disappearance of the N2‐H peak at *δ* 12.26 ppm on ^1^H‐NMR of the parent benzothiadiazine‐1,1‐dioxide **1**, indicated the successful synthesis of the derivatives **2–14** through the S_N_ reaction. This was also further corroborated by the disappearance of the NH‐peak stretching from compound **1** at 3253 cm^−1^ on IR spectra of all compounds **2–14**. Peaks attributed to the resonance of the aromatic H of the benzothiadiazine‐1,1‐dioxide core in all the synthesised compounds were found in the *δ* 7.81–7.33 ppm region as sequence of doublets and triplets with *J* values varying from 7.8 to 7.6 Hz. The formation of the target compounds was also confirmed on the ^1^H spectra having a characteristic singlet in 8.12–7.95 ppm, which was attributed to the resonance of the acidic proton H‐3.

The ^1^H spectra of EG oligomeric analogues **2–8** presented with a triplet in the *δ* 4.33–4.20 ppm region, which is attributed to H‐10 coupling with neighbouring H‐9 (*J* 4.9–5.1 Hz). This also confirms the successful linkage of the ethylene glycol side chain to the parent core structure. Compounds **3**, **4** and **5** displayed singlets of the methyl protons in the *δ* 3.09–2.11 ppm range. The resonance of methylene protons of the terminal ethoxy group in compounds **6**, **7** and **8** were clearly observed as a coalesced quartet in the *δ* 3.65–3.35 ppm range on their ^1^H spectra. IR further confirmed the formation of compound **2** with the presence of the broad O−H vibration peak at 3508 cm^−1^.

The aryl‐terminated compounds **9–12** displayed a triplet *ca. δ* 4.55 ppm that was assigned to H‐10. The increasing electron withdrawing groups (EWG), Cl, Br, and NO_2_, in *para*‐position of the phenyl of compounds **10**, **11** and **12**, respectively, in sequence of appearance had no significant impact on the chemical shift of this triplet. Furthermore, peaks of the aromatic hydrogens of the additional phenyl ring in compounds **9–12** are visible as doublets in the *δ* 8.00–6.50 ppm. IR also confirms the presence of each compound's unique electron withdrawing group through the stretching of the bond of the linked C atom, C−Br (572 cm^−1^), C−Cl (821 cm^−1^), and N−O (1311 cm^−1^).

Compound **13** possessed two vinylic *cis* H‐13_a_ and H‐13_b_ hydrogens which were assigned as coalesced double of doublets at *δ* 5.17 and *δ* 5.08 ppm, respectively and the corresponding ^2^ *J* coupling constant of 10.4 Hz. The multiplet *ca. δ* 5.80 ppm was assigned to the resonance of H‐12 as result of its relatively long‐distance coupling with vinyl protons Ha and Hb (*J* 15.8 and 17.1, respectively), and short distance coupling with neighbouring H‐11 protons. Analogue **14** was confirmed by the disappearance of the H‐10 peak *ca* 4.30 ppm as well as the distinctive absorption of the weak C≡C stretching at 2121 cm^−1^ on the IR spectrum.

On the ^13^C NMR, the aromatic carbons all appeared as singlets in the range of *δ* 163.7–113.2 ppm depending on the R‐substituent attached on the compound. IR further confirmed the structures of all synthesised analogues through the presence of the distinctive sulfonamide‐associated S=O stretching in the range of 1400–1349 cm^−1^ range.

The structures of compounds **1–14** were further confirmed by HRMS, in which the calculated *m/z* values reflected the experimental ones with an accuracy <3 ppm.^[28]^

Furthermore, purity of the synthesised compounds was found to be in the 87–99 % range as assessed by HPLC.

### 2.2 Biological Activity

The synthesised benzothiadiazine‐1,1‐dioxide derivatives were screened for potential *in vitro* antileishmanial activity against the promastigotes and the amastigotes of two *Leishmania* strains. These strains were *L. donovani* strain 9515, which causes the disease's more lethal form, VL, and *L. major* NIH S, which imparts CL, the most prevalent form of leishmaniasis.[^29^, ^30^] A single‐point screening at 10 μM of compound was initially used for the antileishmanial screenings according to the guidelines of Katsuno *et al*.,^[31]^ which place emphasis on compounds with IC_50_ below 10 μM and 1 μM for the classification as antileishmanial drug hits and leads, respectively. However, as very little activity was detected for all the compounds (majority growth inhibition <10 %) against both promastigote and intracellular amastigote forms of the parasite (Table 1), the maximum concentration was increased to 2 mM for subsequent testing to potentially determine SAR (Table 2). Amphotericin B (AmB) served as the reference antileishmanial drug. Cytotoxicity profiles of the synthesised derivatives were determined using Vero cells, with emetine (Em) serving as a positive control. The biological results are reported in Tables 1 and 2.

**Table 1:** Growth inhibition (%) of compounds against *L. donovani* and *L. major* promastigotes and intramacrophage amastigotes at 10 μM.

**Compd**.	*L. donovani* strain 9515			*L. major* strain IR‐173	
	Antipromastigote growth inhibition (%)	Anti‐amastigote growth inhibition (%)		Antipromastigote growth inhibition (%)	Anti‐amastigote growth inhibition (%)
**1**	10	0		0	0
**2**	0	0		0	0
**3**	2	0		0	0
**4**	0	0		0	0
**5**	5	0		0	0
**6**	2	0		0	0
**7**	0	0		0	0
**8**	3	0		0	0
**9**	7	0		58	0
**10**	0	0		0	0
**11**	3	0		0	0
**12**	9	0		0	0
**13**	15	0		0	0
**14**	1	0		23	0
**Em**	–	–		–	–
**AmB**	100	100		100	100

**Table 2:** Cytotoxicity (μM±Standard deviation) and antileishmanial activity of compounds against *L. donovani* and *L. major* promastigotes after growth inhibition (%) at maximum concentration of 2 mM.

**Compd**.	Vero IC_50_ (μM)	*L. donovani* 9515			*L. major* NIH S	
		antipromastigote IC_50_ (μM)	SI_1_		antipromastigote IC_50_ (μM)	SI_2_
**1**	323.10±215.24	611.75±30.90	0.53		860.75±46.46	0.38
**2**	752.30±173.67	839.30±41.86	0.90		1 073.30±162.21	0.70
**3**	501.90±151.60	481.85±7.57	1.04		701.25 ±40.80	0.72
**4**	988.50±10.61	701.65±4.17	1.41		918.80±22.34	1.08
**5**	999.10±16.83	830.05±10.54	1.20		1 117.95±333.83	0.89
**6**	470.45±224.93	333.20±45.11	1.41		586.15±47.02	0.80
**7**	727.00±61.66	426.05±34.72	1.71		491.30±28.57	1.48
**8**	921.90±93.90	809.20±20.22	1.14		909.00±123.74	1.01
**9**	975.30±3.68	153.40±7.92	6.36		103.60±4.53	9.41
**10**	663.10±24.04	905.95±62.15	0.73		1 400.50±68.59	0.47
**11**	747.20±187.81	290.40±21.35	2.57		300.20±3.68	2.49
**12**	666.60±57.98	410.90±27.86	1.62		783.40±408.14	0.85
**13**	530.70±15.70	359.90±12.45	2.23		455.35±9.55	1.17
**14**	534.70±21.78	237.65±14.50	2.25		185.00±22.06	2.89
**Em**	0.08±0.01					
**AmB**	57.77±3.22	0.02±0.00	2 888.50		0.11±0.01	525.18

SI_1_: Selectivity index=IC_50_ Vero/*L. donovani* 9515 antipromastigote IC_50_; SI_2_: Selectivity index=IC_50_ Vero/*L. major* antipromastigote IC_50_.

In general, no significant indications of potential antipromastigote activity were detected. All the compounds presented with IC_50_ values above 10 μM, disqualifying them from being deemed as potential antileishmanial hits.^[31]^ Their cytotoxicity IC_50_ values were also higher than 100 μM, deeming them non‐toxic but resulting in selectivity indices indicative of non‐intrinsic antipromastigote activity (SI<10).

As with the antipromastigote assay, no activity could be detected against the intracellular amastigote at 10 μM. Furthermore, 2 mM of the compounds presented with signs of host cell death (reduced macrophage populations and increased viable extracellular promastigote forms compared to the untreated control). Only compounds **5**, **7**, **9** and **11**–**14** presented with both host cell and parasite death, albeit only for *L. major*. Thus, due to the lack of sufficient antileishmanial activity observed at concentrations deemed both suitable (10 μM) and excessive (2 mM) for hit identification (according to the criteria of Katsuno et al.^[31]^) during the dual‐point screening, anti‐amastigote and host cell, IC_50_ determinations were not undertaken.

### 2.3 Predicted Physicochemical and Pharmacokinetic Properties

Due to the overall lack of significant biological activity and cytotoxicity, the *in silico* physicochemical properties of the compounds were determined to potentially elucidate contributing issues.

The preferred route of drug delivery is oral administration on account of the associated advantages, such as higher patient compliance, non‐invasiveness, and ease of administration.^[32]^ Lipinski's rule of five (LRO5)^[33]^ can be used to evaluate a compound's likelihood of absorption after oral administration and subsequent drug‐likeness.^[34]^ The rules consider not only the lipophilicity of a compound, which is required for permeation of the gastrointestinal (GI) tract's lipid bilayer, but also aqueous solubility. For pharmaceuticals to be present in adequate concentrations at the surface of the intestinal epithelium to facilitate absorption, they must have physicochemical qualities that allow solubilisation in water in the gastrointestinal tract.^[34]^ Furthermore, as compounds that comply with Lipinski's rule of five are more likely to have adequate absorption following oral administration, the SwissADME web tool was used to forecast the physicochemical properties of the synthesised analogues, and the results were recorded in Table 3.

**Table 3:** Molecular and ADME properties of the synthesised 1,2,4‐benzothiadiazine‐1,1‐dioxide derivatives as predicted by the SwissADME web tool, http://www.swissadme.ch.

Compd.	MW^[a]^ (g/mol)	Log*P*_o/w_^[b]^	Log *S* ^[c]^		RB^[f]^	HBD^[g]^	HBA^[h]^	PSA (Å^2^)^[i]^	Lipinski's violation^[j]^	GI Absorption^[k]^	Drug‐likeness^[l]^	Leadlikeness^[m]^
			ESOL^[d]^	Ali^[e]^								
1	182.20	0.67	−1.44	−1.12	0	1	3	66.91	0	High	Yes	No
2	226.25	0.43	−1.34	−1.08	2	1	4	78.35	0	High	Yes	No
3	240.28	0.98	−1.68	−1.41	3	0	4	67.35	0	High	Yes	No
4	284.33	1.08	−1.62	−1.45	6	0	5	79.58	0	High	Yes	Yes
5	328.38	1.11	−1.57	−1.49	9	0	6	85.81	0	High	Yes	No
6	254.31	1.25	−1.92	−1.80	4	0	4	67.35	0	High	Yes	Yes
7	298.36	1.39	−1.86	−1.83	7	0	5	76.58	0	High	Yes	Yes
8	342.41	1.47	−1.81	−1.87	10	0	6	85.81	0	High	Yes	No
9	302.35	2.33	−3.28	−3.29	4	0	4	67.35	0	High	Yes	Yes
10	381.24	2.82	−4.19	−4.01	4	0	4	67.35	0	High	Yes	No
11	336.79	2.69	−3.88	−3.94	4	0	4	67.35	0	High	Yes	Yes
12	347.35	1.54	−3.34	−4.08	5	0	6	113.17	0	High	Yes	Yes
13	266.32	1.52	−2.08	−2.08	5	0	4	67.35	0	High	Yes	Yes
14	220.25	1.28	−1.87	−1.49	1	0	3	58.12	0	High	Yes	No

[a] Molecular weight. [b] Calculated logP (consensus log P). [c] Predicted aqueous solubility, where log S is the logarithm of the amount of compound (in moles) able to dissolve a liter of water. [d] ESOL=estimated aqueous solubility, calculated using a topological method.^[35]^ [e] Calculated using a topological method^[36]^ with log S scale: insoluble <−10 < poorly <−6 < moderately <−4 < soluble <−2 very soluble <0 highly <. [f] Number of rotatable bonds (RB). [g] Number of hydrogen bond donors (HBD). [h] Number of hydrogen bond acceptors (HBA). [i] Polar surface area (PSA), RB ≤10 and PSA ≤140 Å^2^–good oral bioavailability.^[37]^ [j] Determined with reference to Lipinski's rule of five: MW ≤500 g/mol; LogP≤5; RB≤10; HBD≤5 (NH or OH); HBA ≤10 (N or/and O), no more than one violation allowed.^[33]^ [k] Qualitative determination in humans. [l] Determined by using Lipinski's rule of five: MW ≤500 g/mol; LogP ≤5; HBD ≤5; HBA ≤10; no more than one violation allowed.^[33]^ [m] According to Teague: 250 <MW<350, LogP_o/w_ <3.5 and RB <7.^[38]^ All values in this table were calculated using SwissADME web tool, http://www.swissadme.ch . ^[39]^

All the synthesised compounds have physicochemical properties well within the ranges set out by Lipinski^[33]^ and thus, they comply with the Lipinski's rule of five. Consequently, these analogues were possible drug‐like with a high predicted likelihood for GI absorption through passive diffusion. Compounds **4**, **6**, **7**, **9**, **11**, **12** and **13** also display physicochemical properties that allow them to possibly be classified as lead‐like.

The lack of overall *in vitro* biological activity was accordingly not attributed to solubility issues, as the compounds were all soluble in the growth medium (≤1 mM), supporting the predicted physicochemical properties of the compounds (Table 3). Interestingly, although the *in silico* identified lead‐like compounds did not present with lead‐relevant biological activities, analogues **6**, **7**, **9**, **11**, **12** and **13** did, however, present with the highest antipromastigote activity against both *Leishmania* spp. tested, apart from **14** which did not qualify as lead‐like but also presented with high activity. Furthermore, **7**, **9**, **11**, **12**, **13** and **14** were also the only compounds that killed both parasite and host cell during the 2 mM screening for *L. major* anti‐amastigote activity. These patterns were also detected in the host cell viability, but not observed for the Vero cytotoxicity data. Accordingly, the combination of molecular weight, log P and/or number of rotatable bond levels, which together contribute to the druglikeness of a compound,^[38]^ may have contributed to the higher biological activities of these compounds.

However, two hypotheses may be advanced to justify the overall lack of antileishmanial activity of the analogues: (a) reduced target site affinity due to the modifications of the parent structure **1**; (b) cell culture protein binding. Indeed, with regards to (a), if compounds do not bind to the active site of the targeted protein, in this case, the parasitic enzyme *β*‐CA,^[15]^ no pharmacological effect will be obtained, which is what has been observed for many of the compounds synthesised during this study. Furthermore, (b) the undesired binding of a drug to cell culture medium serum and/or proteins such as albumin may result in the formation of drug‐protein complex, and although this binding is reversible in nature, the short‐term negative effect is a contribution to lowering the biological activity of that drug.^[40]^ Such binding is influenced by a variety of factors including chemical structure of the drug/compound and its pKa, type of targeted protein and pH of the surrounding plasma.^[40]^ The antileishmanial assay media of this study are comprised of *inter alia* 10 % foetal bovine serum (FBS), which contains albumin (±2.38 g/100 mL). The human albumin binding of 1,2,4‐benzothiadiazine‐1,1‐dioxide and its analogues has been reported with the unsubstituted synthon being bound less.^[41]^ Alternatively, ethylene oxide and its macromolecule, poly(ethylene oxide) (PEO) *have been suggested to interact* with *protein* molecules through hydrogen bonding, hydrophobic and ionic interactions.[^42^, ^43^]

Previously reported active analogues were methyl‐substituted in position C‐3 of the benzothiadiazine‐1,1‐dioxide core. The substitution of the methyl with a phenyl ring curtailed the antileishmanial activity.^[25]^ These early findings and the outcome of the current study, i. e., the complete loss of antileishmanial activity upon replacement of the C‐3 methyl group with H, underscores (1) the significance of the methyl as a potential protein binding moiety, and (2) ethylene oxide as an unfavourable moiety for the enhancement of antileishmanial efficacy *in vitro*.

## 3 Conclusions

A series of novel benzothiadiazine‐1,1‐dioxide derivatives featuring ethylene glycol moieties were synthesised in poor to excellent yields (17–94 %) following a three‐step process. The synthetic methods used included cyclisation and Schiff base condensation, bromination and nucleophilic substitution (S_N_). The newly synthesised compounds were screened *in vitro* against two strains of *Leishmania* to determine their efficacy and cytotoxicity profiling using Vero cells. All the compounds were non‐toxic (>100 μM) to Vero cells, but an overall lack of antileishmanial activity was reported despite their promising predicted pharmacokinetic and physicochemical properties. Only analogue **9** featuring phenoxy moiety displayed moderate antipromastigote activity (IC_50_ <155 μM against both tested parasite strains). The lack of biological activity could possibly be attributed to reduced affinity to target proteins and/or the binding of the compounds to cell culture proteins. Future endeavour will focus on investigating the antileishmanial activity of novel benzothiadiazine‐1,1‐dioxide derivatives comprising C‐3 alkyl substituent with N‐side chains devoid of protein binding capacity such as EG.

## Material and Methods

### Materials

All chemical reagents used in this study were purchased from Sigma‐Aldrich (Johannesburg, South Africa). All solvents and the drying agent anhydrous magnesium sulphate were purchased from Associated Chemical Enterprises, ACE (South Africa). The base, potassium carbonate, was purchased from Merck, South Africa. All chemicals and reagents were of analytical grade and no further purification was required.

### General Procedures

The ^1^H and ^13^C nuclear magnetic resonance (NMR) spectra was recorded on a Bruker Avance^TM^ III 600 spectrometer at a frequency of 600 and 150 MHz, respectively, in deuterated dimethyl sulfoxide (DMSO‐*d_6_*). The residual protons of the solvent were used as a reference, and the chemical shifts were reported in parts per million (ppm). The abbreviations of the splitting patterns were as follows: singlet (s), doublet (d), doublet of doublet (dd), doublet of doublets of doublets (ddd), triplet (t), triplet of triplets (tt), quartet (q) and multiplet (m). High resolution mass spectrometry (HRMS) was recorded on a Bruker MicroTOF Q II mass spectrometer. It was equipped with an atmospheric pressure chemical ionisation (APCI) source set at 200 °C or 180 °C and analysed using Bruker Compass Data Analysis 4.0 software. A full scan ranged from 50–1500 *m/z* at a capillary voltage of 4500 V, an end plate offset voltage of −500 V, with the nebuliser set at 1.6 and 0.4 Bar, respectively, and a collision cell RF voltage of 100 Vpp. Infrared (FTIR) spectra were recorded on a Bruker Alpha‐P FTIR instrument. A BÜCHI melting point B‐545 instrument was used to determine melting points (mp) and were uncorrected. Thin layer chromatography (TLC) was performed on silica gel plates (^60^F_254_) that was acquired from Merck (South Africa).

Purity determination (HPLC): A Shimadzu iNexera LC‐2040 C system consisting of a quaternary pump, solvent degasser, autosampler, column oven, and photodiode array (PDA) detector was used to determine the purity of the compounds. LabSolutions software was used to control all instrument components and acquire, analyze, and store the data. Chromatographic separation was achieved on a Venusil® XBP C18(2); 2,1x50 mm; 3 μm; column (Agela technologies, Newar, Germany). Mobile phase A consisted of 0.1 % formic acid and B of MeOH containing 0.1 % formic acid. Gradient elution was performed starting at 90 % A for 0.5 min increasing to 100 % B at 3 min where it was kept until 6 min. The system was returned to 10 % A and allowed to re‐equilibrate until 10 min. The autosampler was maintained at 6 °C, and the PDA detector stored UV data over the range of 190–600 nm, and detection was conducted at 270 nm. The ratio of peak areas in the chromatograms was used to express the purity in percentage. All chromatograms are provided as Supplementary information.

### Synthesis

#### Synthesis of 2*H*‐Benzo[*e*][1,2,4]thiadiazine‐1,1‐dioxide 1

This intermediate was brought about as previously described by Mangwegape *et al*.^[25]^ To a two‐necked round bottom flask, 2‐aminobenzenesulfonamide (5.81 mmol, 1 equiv.) and triethyl orthoformate (40.6 mmol, 7 equiv.) were added, and the resulting mixture was refluxed at 100 °C for 12 hours. This reaction was monitored by thin‐layer chromatography (TLC) and cooled to room temperature upon completion. This resulted in the formation of a precipitate that was filtered off and washed twice with diethyl ether (Et_2_O) (2x10 mL) to yield the pure benzothiadiazine‐1,1‐dioxide as a white solid product.

White powder; yield: 94 %; mp 223–226 °C (Lit. 270 °C^[25]^); IR_*Vmax*_ (cm^−1^): 3253 (N−H) (w), 3104 (C−H) (w), 1475 (C=C) (m), 1370 (S=O) (m), 1275 (C−N) (m), 762 (C−H) (s); ^1^H‐NMR (600 MHz, DMSO‐*d_6_*) *δ* (ppm): 12.26 (s, 1H, H‐2), 7.95 (s, 1H, H‐3), 7.81 (d, *J* 7.8 Hz, 1H, H‐8), 7.68 (t, *J* 7.8 Hz, 1H, H‐7), 7.47 (t, *J* 7.6 Hz, 1H, H‐6), 7.33 (d, *J* 7.6 Hz, 1H, H‐5); ^13^C NMR (150 MHz, DMSO‐*d_6_*) *δ* (ppm): 148.0 (C‐3), 135.0 (C‐4a), 133.6 (C‐6), 127.2 (C‐8), 124.1 (C‐7), 122.6 (C‐8b), 117.6 (C‐5); HRMS‐APCI *m/z*: 183.0227 [M+H]^+^ (calcd. for C_7_H_7_N_2_O_2_S^+^, 183.0223; Δ*m/z* =0.0004); Purity (HPLC): 99 %.

#### Synthesis of Brominated Ethylene Glycol Intermediates

Briefly described, in a round bottom flask, ethylene glycol or derivatives (11 mmol, 3 equiv.) was dissolved in 30 mL of dry chloroform (CHCl_3_). While stirring at 0 °C, phosphorus tribromide (PBr_3_) (3.69 mmol, 1 equiv.) was added. The reaction was then warmed up to room temperature, refluxed for 12 hours at 60 °C and monitored by TLC. Afterwards, the reaction was quenched with water (10 mL), the aqueous phase was extracted with dichloromethane (DCM) (3x20 mL). The organic phase was dried over anhydrous magnesium sulphate (MgSO_4_), filtered, and concentrated on the vacuum to yield a clear, oily product. These intermediates were used in subsequent reactions without further purification.

Note: Dry Chloroform was prepared from drying reagent grade chloroform on molecular sieves prior to use.

#### Synthesis of Benzothiadiazine‐1,1‐dioxide Derivatives 2–14

Benzothiadiazine‐1,1‐dioxide **1** (5.49 mmol, 1 equiv.) was dissolved in dry DMF (4 mL) and added to a round bottom flask. Thereafter, potassium carbonate (1 equiv.) and brominated ethylene glycol (6.57 mmol, 1.2 equiv.) were added. The reaction was stirred at room temperature for 12 hours and monitored by TLC. Upon completion, the reaction mixture was poured into ice water (20 mL) causing the formation of a precipitate that was filtered and washed with filtered tap water. Compounds **2**, **8** and **14** were further purified using column chromatography on silica gel eluting with ethyl acetate. Analogue **7** was further purified by column chromatography on silica gel with DCM/MeOH (95 : 5, v/v) as eluent.

#### 2‐(2‐Hydroxyethyl)‐2*H*‐benzo[*e*][1,2,4]thiadiazine‐1,1‐dioxide 2

White powder; yield: 33 %; mp 167–170 °C; IR_*Vmax*_ (cm^−1^): 3508 (OH) (b), 3105 (C−H) (w), 1413 (C−N) (w), 1393 (S=O) (w), 767 (C−H) (s); ^1^H‐NMR (600 MHz, DMSO‐*d_6_*) *δ* (ppm): 8.00 (s, 1H, H‐3), 7.91 (dd, *J* 7.9, 1.5 Hz, 1H, H‐8), 7.77 (ddd, *J* 7.9, 7.3, 1.5 Hz, 1H, H‐7) 7.61 (d, *J* 7.6 Hz, 1H, H‐5), 7.55 (t, *J* 7.6 Hz, 1H, H‐6), 5.12 (t, *J* 5.1 Hz, 1H, H‐11), 4.20 (t, *J* 5.1 Hz, 2H, H‐10), 3.69 (q, *J* 5.1 Hz, 2H, H‐9); ^13^C NMR (150 MHz, DMSO‐*d_6_*) *δ* (ppm): 152.0 (C‐3), 135.5 (C‐4a), 133.8 (C‐6), 127.3 (C‐8), 125.1 (C‐7), 123.7 (C‐8b), 117.1 (C‐5), 58.1 (C‐10), 52.8 (C‐9); HRMS‐APCI *m/z*: 227.0484 [M+H]^+^ (calcd for C_9_H_11_N_2_O_3_S^+^, 227.0485; Δ*m/z* =0.0001); Purity (HPLC): 99 %.

#### 2‐(2‐Methoxyethyl)‐2*H*‐benzo[*e*][1,2,4]thiadiazine‐1,1‐dioxide 3

White powder; yield: 66 %; mp 107–110 °C; IR_*Vmax*_ (cm^−1^): 3072 (C−H) (w), 1418 (C−N) (m), 1400 (S=O) (w), 1067 (C−O) (m), 770 (C−H) (s); ^1^H‐NMR (600 MHz, DMSO‐*d_6_*) *δ* (ppm): 8.00 (s, 1H, H‐3), 7.89 (d, *J* 7.2 Hz, 1H, H‐8), 7.76 (t, *J* 7.2 Hz, 1H, H‐7), 7.62 (d, *J* 7.6 Hz, 1H, H‐5), 7.54 (t, *J* 7.6 Hz, 1H, H‐6), 4.31 (t, *J* 5.0 Hz, 2H, H‐10), 3.62 (t, *J* 5.0 Hz, 2H, H‐9), 3.26 (s, 3H, H‐11); ^13^C NMR (151 MHz, DMSO‐*d_6_*) *δ* (ppm): 152.0 (C‐3), 135.5 (C‐4a), 133.9 (C‐6), 127.5 (C‐8), 125.1 (C‐7), 123.7 (C‐8b), 117.2 (C‐5), 69.2 (C‐10), 59.0 (C‐11), 50.2 (C‐9); HRMS‐APCI *m/z*: 241.0651 [M+H]^+^ (calcd for C_10_H_13_N_2_O_3_S^+^. 241.0642; Δ*m/z* =0.0009); Purity (HPLC): 97 %.

#### 2‐[2‐(2‐Methoxyethoxy)ethyl]‐2*H*‐benzo[*e*][1,2,4]thiadiazine‐1,1‐dioxide 4

White powder; yield: 64 %; mp 80–83 °C; IR_Vmax_ (cm^−1^): 3054 (C−H) (w), 1404 (C−N) (m), 1349 (S=O) (w), 1095 (C−O) (s), 767 (C−H) (s); ^1^H‐NMR (600 MHz, DMSO‐*d_6_*) *δ* (ppm) 8.00: (s, 1H, H‐3), 7.89 (d, *J* 7.9 Hz, 1H, H‐8), 7.76 (t, *J* 7.9 Hz, 1H, H‐7), 7.62 (d, *J* 7.6 Hz, 1H, H‐5), 7.54 (t, *J* 7.6 Hz, 1H, H‐6), 4.30 (t, *J* 4.88 Hz, 2H, H‐10), 3.71 (t, *J* 5.0 Hz, 2H, H‐11), 3.52 (t, 2H, *J* 5.0 Hz, H‐12), 3.37 (t, 2H, *J* 4.88 Hz, H‐9), 3.15 (s, 3H, H‐13); ^13^C NMR (150 MHz, DMSO‐*d_6_*) *δ* (ppm): 152.0 (C‐3), 135.4 (C‐4a), 133.8 (C‐6), 127.4 (C‐8), 125.1 (C‐7), 123.6 (C‐8b), 117.2 (C‐5), 71.9 (C‐12), 70.3 (C‐11), 67.5 (C‐10), 58.7 (C‐13), 50.2 (C‐9); HRMS‐APCI *m/z*: 285.0913 [M+H]^+^ (calcd for C_12_H_17_N_2_O_4_S^+^, 285.0904; Δ*m/z* =0.0009); Purity (HPLC): 87 %.

#### 2‐{2‐[2‐(2‐Methoxyethoxy)ethoxy]ethyl}‐2*H*‐benzo[*e*][1,2,4]thiadiazine‐1,1‐dioxide 5

Light yellow powder; yield: 50 %; mp 81–84 °C; IR_*Vmax*_ (cm^−1^): 3043 (C−H) (w), 1412 (C−N) (m), 1350 (S=O) (w), 1025 (C−O) (s), 790 (C−H) (m); ^1^H‐NMR (600 MHz, DMSO‐*d_6_*) *δ* (ppm): 8.00 (s, 1H, H‐3), 7.89 (dd, *J* 7.9, 1.5 Hz, 1H, H‐8), 7.76 (ddd, *J* 7.9, 7.3, 1.5 Hz, 1H, H‐7), 7.62 (d, *J* 7.6 Hz, 1H, H‐5), 7.54 (t, *J* 7.6 Hz, 1H, H‐6), 4.30 (t, *J* 5.1 Hz, 2H, H‐10), 3.72 (t, *J* 5.1 Hz, 2H, H‐9), 3.53 (dd, *J* 5.7, 3.7 Hz, 2H, H‐11), 3.46 (dd, *J* 5.7, 3.7 Hz, 2H, H‐12), 3.43 (dd, *J* 5.7, 3.8 Hz, 2H, H‐13), 3.35 (dd, *J* 5.7, 3.8 Hz, 2H, H‐14), 3.20 (s, 3H, H‐15); ^13^C NMR (150 MHz, DMSO‐*d_6_*) *δ* (ppm): 152.0 (C‐3), 135.5 (C‐4a), 133.8 (C‐6), 127.4 (C‐8), 125.1 (C‐7), 123.7 (C‐8b), 117.2 (C‐5), 71.8 (C‐14), 70.6 (C‐13), 70.4 (C‐12), 70.3 (C‐11), 67.6 (C‐10), 58.6 (C‐15), 50.3 (C‐9); HRMS‐APCI *m/z*: 329.1167 [M+H]^+^ (calcd for C_14_H_21_N_2_O_5_S^+^, 329.1166; Δ*m/z*=0.0001); Purity (HPLC): 92 %.

#### 2‐(2‐Ethoxyethyl)‐2*H*‐benzo[*e*][1,2,4]thiadiazine‐1,1‐dioxide 6

White powder; yield: 50 %; mp 125–128 °C; IR_*Vmax*_ (cm^−1^): 3078 (C−H) (w), 1403 (C−N) (m), 1349 (S=O) (w), 1095 (C−O) (m), 787 (C−H) (m); ^1^H‐NMR (600 MHz, DMSO‐*d_6_*) *δ* (ppm): 8.00 (s, 1H, H‐3), 7.89 (d, *J* 7.9 Hz, 1H, H‐8), 7.76 (t, *J* 7.9 Hz, 1H, H‐7), 7.63 (d, *J* 7.6 Hz, 1H, H‐5), 7.54 (t, *J* 7.6 Hz, 1H, H‐6), 4.30 (t, *J* 5.0 Hz, 2H, H‐10), 3.64 (*q, *J* 5.0 Hz, 2H, H‐11), 3.42 (q, *J* 7.0 Hz, 2H, H‐9), 1.03 (t, *J* 7.0 Hz, 3H, H‐12); ^13^C NMR (150 MHz, DMSO‐*d_6_*) *δ* (ppm): 151.8 (C‐3), 135.2 (C‐4a), 133.7 (C‐6), 127.3 (C‐8), 124.9 (C‐7), 123.4 (C‐8b), 117.0 (C‐5), 66.6 (C‐10), 66.2 (C‐11), 50.2 (C‐9), 15.3 (C‐12); HRMS‐APCI *m/z*: 255.0799 [M+H]^+^ (calcd for C_11_H_15_N_2_O_3_S^+^, 255.0798; Δ*m/z* =0.0001); Purity (HPLC): 99 %.

*q Coalesced quartet

#### 2‐[2‐(2‐Ethoxyethoxy)ethyl]‐2*H*‐benzo[*e*][1,2,4]thiadiazine‐1,1‐dioxide 7

Light brown powder; yield: 28 %; mp 82–85 °C; IR_*Vmax*_ (cm^−1^): 3053 (C−H) (w), 1409 (C−N) (w), 1349 (S=O) (w), 1097 (C−O) (m), 762 (C−H) (m); ^1^H‐NMR (600 MHz, DMSO‐*d_6_*) *δ* (ppm) 8.03 (s, 1H, H‐3), 7.93 (d, *J* 7.9 Hz, 1H, H‐8), 7.80 (tt, *J* 7.9, 3.29 Hz, 1H, H‐7), 7.65 (d, *J* 7.6 Hz, 1H, H‐5), 7.57 (t, *J* 7.6 Hz, 1H, H‐6), 4.33 (t, *J* 5.0 Hz, 2H, H‐10), 3.75 (dd, *J* 10.7, 5.5 Hz 2H, H‐11), 3.56–3.54 (m, 2H, H‐12), 3.43 (*q, *J* 5.6 Hz, 2H, H‐13), 3.33 (d, *J* 5.0 Hz, 2H, H‐9), 1.04 (t, *J* 7.0 Hz, 3H, H‐14); ^13^C NMR (151 MHz, DMSO‐*d_6_*) *δ* (ppm) 151.4 (C‐3), 134.8 (C‐4a), 133.2 (C‐6), 126.8 (C‐8), 124.5 (C‐7), 123.0 (C‐8b), 116.6 (C‐5), 69.9 (C12), 69.2 (C11), 66.9 (C‐10), 65.6 (C‐13), 49.6 (C‐9), 15.0 (C‐14); HRMS‐APCI *m/z*: 299.1063 [M+H]^+^ (calcd for C_13_H_19_N_2_O_4_S^+^, 299.1061; Δ*m/z* =0.0002); Purity (HPLC): 99 %.

*q Coalesced quartet

#### 2‐{2‐[2‐(2‐Ethoxyethoxy)ethoxy]ethyl}‐2*H*‐benzo[*e*][1,2,4]thiadiazine‐1,1‐dioxide 8

White powder; yield: 18 %; mp 54–57 °C; IR_*Vmax*_ (cm^−1^): 3066 (C−H) (w), 1405 (C−N) (w), 1394 (S=O) (w), 1096 (C−O) (s), 789 (C−H) (s); ^1^H‐NMR (600 MHz, DMSO‐*d_6_*) *δ* (ppm): 8.01 (s, 1H, H‐3), 7.89 (d, *J* 7.8 Hz, 1H, H‐8), 7.76 (t, *J* 7.8 Hz, 1H, H‐7), 7.62 (d, *J* 7.6 Hz, 1H, H‐5), 7.54 (t, *J* 7.6 Hz, 1H, H‐6), 4.30 (t, *J* 5.0 Hz, 2H, H‐10); 3.71 (*q, *J* 5.0 Hz, 2H, H‐15), 3.52 (t, *J* 4.9 Hz, 2H, H‐12), 3.46 (dd, *J* 9.5, 4.8 Hz, 2H, H‐13), 3.41 (dd, *J* 9.7, 4.9 Hz, 2H, H‐11), 3.39–3.36 (m, 4H, H‐14/9), 1.07 (t, *J* 5.0 Hz, 3H, H‐16); ^13^C NMR (150 MHz, DMSO‐*d_6_*) *δ* (ppm): 152.0 (C‐3), 135.3 (C‐4a), 133.8 (C‐6), 127.4 (C‐8), 125.1 (C‐7), 123.6 (C‐8b), 117.2 (C‐5), 70.5 (C‐12/13), 69.7 (C‐11/14), 67.5 (C‐10), 66.1 (C‐15), 50.2 (C‐9), 15.7 (C‐16); HRMS‐APCI *m/z*: 343.1326 [M+H]^+^ (calcd for C_15_H_23_N_2_O_5_S^+^, 343.1323; Δ*m/z* =0.0003); Purity (HPLC): 96 %.

*q Coalesced quartet

#### 2‐(2‐Phenoxyethyl)‐2*H*‐benzo[*e*][1,2,4]thiadiazine‐1,1‐dioxide 9

White powder; yield: 72 %; mp 219–222 °C; IR_*Vmax*_ (cm^−1^): 3117 (C−H) (w), 1590 (C=C) (m), 1406 (C−N) (w), 1390 (S=O) (w), 1085 (C−O) (m), 781 (C−H) (w); ^1^H‐NMR (600 MHz, DMSO‐*d_6_*) *δ* (ppm): 8.14 (s, 1H, H‐3), 7.90 (d, *J* 7.9 Hz, 1H, H‐8), 7.78 (t, *J* 7.9 Hz, 1H, H‐7), 7.71 (d, *J* 7.6 Hz, 1H, H‐5), 7.55 (t, *J* 7.6 Hz, 1H, H‐6), 7.27 (t, *J* 7.9 Hz, 2H, H‐13), 6.94 (t, *J* 7.9 Hz, 1H, H‐14), 6.89 (d, *J* 7.9 Hz, 1H, H‐12), 4.56 (t, *J* 5.2 Hz, 2H, H‐10), 4.29 (t, *J* 5.2 Hz, 2H, H‐9); ^13^C NMR (150 MHz, DMSO‐*d_6_*) *δ* (ppm): 158.3 (C‐11), 152.0 (C‐3), 135.3 (C‐4a), 133.8 (C‐6), 130.1 (C‐13), 127.4 (C‐8), 125.0 (C‐7), 123.6 (C‐8b), 121.7 (C‐5), 117.1 (C‐14), 115.1 (C‐12), 65.1 (C‐10), 49.7 (C‐9); HRMS‐APCI *m/z*: 303.0800 [M+H]^+^ (calcd for C_15_H_15_N_2_O_3_S^+^, 303.0798; Δ*m/z* =0.0002); Purity (HPLC): 91 %.

#### 2‐[2‐(4‐Bromophenoxy)ethyl]‐2*H*‐benzo[*e*][1,2,4]thiadiazine‐1,1‐dioxide 10

White powder; yield: 47 %; mp 217–220 °C; IR_*Vmax*_ (cm^−1^): 3071 (C−H) (w), 1592 (C=C) (m), 1407 (C−N) (w), 1392 (S=O) (w), 1103 (C−O) (m), 782 (C−H) (w), 572 (C−Br) (m); ^1^H‐NMR (600 MHz, DMSO‐*d_6_*) *δ* (ppm) 8.15 (s, 1H, H‐3), 7.91 (d, *J* 7.6 Hz, 1H, H‐8), 7.80 (t, *J* 7.6 Hz, 1H, H‐7), 7.72 (d, *J* 7.5 Hz, 1H, H‐5), 7.57 (t, *J* 7.5 Hz, 1H, H‐6), 7.45 (d, *J* 8.6 Hz, 2H, H‐13), 6.89 (d, *J* 8.6 Hz, 2H, H‐12), 4.57 (s, 2H, H‐10), 4.30 (s, 2H, H‐9); ^13^C NMR (150 MHz, DMSO‐*d_6_*) *δ* (ppm): 157.7 (C‐11), 152.1 (C‐3), 135.3 (C‐4a), 133.9 (C‐6), 132.8 (C‐13), 127.6 (C‐8), 125.1 (C‐7), 123.6 (C‐8b), 117.5 (C‐12), 117.3 (C‐5), 113.2 (C‐14), 65.5 (C‐10), 49.6 (C‐9); HRMS‐APCI *m/z*: 380.9884 [M+H]^+^ (calcd for C_15_H_14_BrN_2_O_3_S^+^, 380.9904; Δ*m/z* =0.002); Purity (HPLC): 98 %.

#### 2‐[2‐(4‐Chlorophenoxy)ethyl]‐2*H*‐benzo[*e*][1,2,4]thiadiazine‐1,1‐dioxide 11

White powder; yield: 69 %; mp 189–192 °C; IR_*Vmax*_ (cm^−1^): 3065 (C−H) (w), 1594 (C=C) (m), 1407 (C−N) (w), 1392 (S=O) (w), 1103 (C−O) (m), 821 (C−Cl) (m), 783 (C−H) (w); ^1^H‐NMR (600 MHz, DMSO‐*d_6_*) *δ* (ppm): 8.16 (s, 1H, H‐3), 7.92 (d, *J* 7.9 Hz, 1H, H‐8), 7.81 (t, *J* 7.9 Hz, 1H, H‐7), 7.73 (d, *J* 7.5 Hz, 1H, H‐5), 7.57 (t, *J* 7.5 Hz, 1H, H‐6), 7.33 (d, *J* 9.0 Hz, 2H, H‐13), 6.94 (d, *J* 9.0 Hz, 2H, H‐12), 4.58 (t, *J* 5.1 Hz, 2H, H‐10), 4.31 (t, *J* 5.1 Hz, 2H, H‐9); ^13^C NMR (150 MHz, DMSO‐*d_6_*) *δ* (ppm): 157.2 (C‐11), 152.1 (C‐3), 135.3 (C‐4a), 133.9 (C‐6), 129.9 (C‐13), 127.5 (C‐7), 125.4 (C‐8b), 125.1 (C‐8), 123.5 (C‐5), 117.2 (C‐14), 116.9 (C‐12), 65.5 (C‐10), 49.6 (C‐9); HRMS‐APCI *m/z*: 337.0421 [M+H]^+^ (calcd for C_15_H_14_ClN_2_O_3_S^+^, 337.0409; Δ*m/z* =0.0012); Purity (HPLC): 99 %.

#### 2‐[2‐(4‐Nitrophenoxy)ethyl]‐2*H*‐benzo[*e*][1,2,4]thiadiazine‐1,1‐dioxide 12

White powder; yield: 82 %; mp 190–193 °C; IR_*Vmax*_ (cm^−1^): 3109 (C−H) (w), 1589 (C=C) (m), 1393 (S=O) (w), 1311 (N−O) (m), 1042 (C−O) (m) 777 (C−H) (m); ^1^H‐NMR (600 MHz, DMSO‐*d_6_*) *δ* (ppm): 8.19 (s, 1H, H‐3), 8.17 (d, *J* 9.1 Hz, 2H, H‐13), 7.90 (d, *J* 7.7 Hz, 1H, H‐8), 7.79 (t, *J* 7.7 Hz, 1H, H‐7), 7.72 (d, *J* 7.5 Hz, 1H, H‐5), 7.55 (t, *J* 7.5 Hz, 1H, H‐6), 7.11 (d, *J* 9.1 Hz, 2H, H‐12), 4.61 (t, *J* 4.8 Hz, 2H, H‐10), 4.46 (t, *J* 4.8 Hz, 2H, H‐9); ^13^C NMR (150 MHz, DMSO‐*d_6_*) *δ* (ppm): 163.7 (C‐11), 152.207 (C‐3), 141.9 (C‐14), 135.4 (C‐4a), 134.0 (C‐6), 127.7 (C‐7), 126.6 (C‐13), 125.2 (C‐8), 123.7 (C‐8b), 117.3 (C‐5), 115.9 (C‐12), 66.3 (C‐10), 49.5 (C‐9); HRMS‐APCI *m/z*: 348.0649 [M+H]^+^ (calcd for C_15_H_14_N_3_O_5_S^+^, 348.0649; Δ*m/z* =0.0000); Purity (HPLC): 89 %.

#### 2‐[2‐(Allyloxy)ethyl]‐2*H*‐benzo[*e*][1,2,4]thiadiazine‐1,1‐dioxide 13

White powder; yield: 47 %; mp 149–152 °C; IR_*Vmax*_ (cm^−1^): 3057 (C−H) (w), 1610 (C=C) (m), 1404 (C−N) (m), 1091 (C−O) (m), 789 (C−H) (m); ^1^H‐NMR (600 MHz, DMSO‐*d_6_*) *δ* (ppm): 8.02 (s, 1H, H‐3), 7.90 (d, *J* 7.7 Hz, 1H, H‐8), 7.75 (t, *J* 7.7 Hz, 1H, H‐7), 7.64 (d, *J* 7.5 Hz, 1H, H‐5), 7.57 (t, *J* 7.5 Hz, 1H, H‐6), 5.80–5.75 (m,, 1H, H‐12), 5.17 (*dd, *J* 10.4, *J* 1.5 Hz, 1H, H‐13a), 5.08 (*ddd, *J* 10.4, *J* 1.5 Hz, 1H, H‐13b), 4.33 (t, *J* 4.6 Hz, 2H, H‐11), 3.94 (d, *J* 4.6 Hz, 2H, H‐10), 3.66 (t, *J* 4.6 Hz, 2H, H‐9); ^13^C NMR (150 MHz, DMSO‐*d_6_*) *δ* (ppm): 152.0 (C‐3), 135.4 (C‐4a), 135.2 (C‐6), 133.8 (C‐12), 127.4 (C‐8), 125.1 (C‐7), 123.6 (C‐8b), 117.2 (C‐5), 117.1 (C‐13), 71.5 (C‐11), 66.6 (C‐10), 50.3 (C‐9); HRMS‐APCI *m/z*: 267.0811 [M+H]^+^ (calcd for C_12_H_15_N_2_O_3_S^+^, 267.0798; Δ*m/z* =0.0013); Purity (HPLC): 94 %.

*ddd Coalesced doublet of doublets

#### 2‐(Prop‐2‐yn‐1‐yl)‐2*H*‐benzo[*e*][1,2,4]thiadiazine‐1,1‐dioxide 14

Light brown powder; yield: 47 %; mp 193–196 °C; IR_*Vmax*_ (cm^−1^): 3267 (C−H) (m), 3075 (C−H) (w), 2121 (C≡C) (w), 1411 (C−N) (m), 1392 (S=O) (w), 785 (C−H) (m); ^1^H‐NMR (600 MHz, DMSO‐*d_6_*) *δ* (ppm): 8.17 (s, 1H, H‐3), 7.91 (d, *J* 7.6 Hz, 1H, H‐8), 7.82 (t, *J* 7.6 Hz, 1H, H‐7), 7.60 (d, *J* 7.8 Hz, 1H, H‐5), 7.59 (t, *J* 7.8 Hz, 1H, H‐6), 5.02 (d, *J* 2.2 Hz, 2H, H‐9), 3.51 (t, *J* 2.3 Hz, 1H, H‐11); ^13^C NMR (150 MHz, DMSO‐*d_6_*) *δ* (ppm): 151.1 (C‐3), 134.9 (C‐4a), 133.7 (C‐6), 127.7 (C‐8), 124.9 (C‐7), 123.8 (C‐8b), 117.2 (C‐5), 78.3 (C‐11), 77.7 (C‐10), 36.8 (C‐9); HRMS‐APCI *m/z*: 221.0370 M+H]^+^ (calcd for C_10_H_9_N_2_O_2_S^+^, 221.0380; Δ*m/z* =0.001); Purity (HPLC): 94 %.

### *In vitro* Biological Evaluation

#### Cytotoxicity Assay

The cytotoxicity of the synthesised compounds was evaluated using the resazurin assay. The assay involves live cells converting oxidized blue resazurin dye to pink, highly fluorescent resorufin through irreversible enzymatic reduction.^[44]^ This non‐toxic reagent is a useful instrument for determining drug toxicity and cell growth.

Dulbecco's modified Eagle's medium (DMEM) with high glucose (Separations) supplemented with 1 % L‐glutamine, non‐essential amino acids, and penicillin‐streptomycin (Sigma Aldrich), and 10 % fetal bovine serum (Thermofisher Scientific) was used to cultivate Vero cells. The cells were kept at 37 °C and 5 % CO_2_ in a humidified environment. For the assay, 96 well plates were prepared by pipetting 100 μL of cell suspension (60 000 cells/mL) per well and incubating the plates for 24 hours. The cells were then treated with seven two‐fold concentration dilutions of: (1) 1 μM emetine dihydrochloride (Sigma Aldrich) solution (positive control); (2) 2 mM experimental compound solutions. Cell‐free growth medium was included as a blank. The treated plates were incubated for 48 hours.

To initiate the resazurin assay, 50 μL of sterile‐filtered resazurin sodium salt (Sigma Aldrich) solution (0.01 % in phosphate‐buffered saline (PBS)) was added to the prepared plates and then incubated for 2 hours. A Thermofisher Scientific's GO Multiscan plate reader was used to measure the absorbance at 570 and 600 nm. SkanIt 4.0 Research Edition software was used to perform the data analysis for every biological replicate. After subtracting background absorbance (600 nm) from the 570 nm absorbance data, the mean absorbance was computed, and the percentage of viable cells was estimated using the formula below:

Cellviability%=(ΔAbssample-ΔAbsblank)/(ΔAbsnegcontrol-ΔAbsblank)X100

GraphPad Prism 5 was used to calculate the IC_50_ and Z‐score for each compound's biological replicate. The mean IC_50_ of the biological replicates and standard deviation (SD) was calculated to determine the final IC_50_ of each compound.

#### Antipromastigote Assay

The antipromastigote activity of synthesised compounds was evaluated using a modified method of the resazurin assay[^45^, ^46^] against two *Leishmania* strains.

*Leishmania donovani* (strain 9515 (MHOM/IN/95/9515)) and *L. major* (strain NIH S (MHOM/SN/74/Seidman)) promastigotes were cultured in M199 with Hank's salts and 0.68 mM L‐glutamine (Sigma Aldrich) supplemented with 0.0005 % hemin, 0.0001 % biotin, 0.1 mM adenine (Sigma Aldrich), 10 % fetal bovine serum, 50 U/mL Penicillin/Streptomycin solution, 4.2 mM sodium bicarbonate and 25 mM Hepes buffer. The promastigotes were maintained at an adjusted pH of 7.3–7.4 growth medium at 26 °C. For the resazurin assay, logarithmic phase promastigotes (1.25x10^6^ cells/mL) were seeded in 96 well plates (Nunc, Thermofisher Scientific) in the presence of seven two‐fold dilution concentrations of compounds for IC_50_ determination, with a maximum concentration of 2 mM and final volume of 100 μL/well. Amphothericin B (10 μM) served as the standard antileishmanial drug and parasite‐free growth medium was included as the blank. The plates were incubated for 48 hours at 26 °C in humidified environment.

Following the initial incubation, 50 μL of resazurin solution (0.01 % in PBS) was added to each well and the plates underwent further incubation at 26 °C for 24 hours in the dark. A Thermofisher Scientific's GO Multiscan plate reader was used to measure absorbance at 570 nm and 600 nm. SkanIt 4.0 Research Edition software was used to perform the data analysis for every biological replicate. Background absorbance (600 nm) was subtracted from the 570 nm absorbance data and the mean absorbance was computed. The cell viability and data analysis were performed as indicated in the cytotoxicity assay.

#### Intramacrophage Antileishmanial Growth Inhibition Assays

A modified resazurin assay[^47^, ^48^] and the two *Leishmania* strains were also used to assess the efficacy of synthesised compounds against intramacrophage parasites.

Human monocytic leukemia (THP‐1) cells were cultured in RPMI 1640 medium with L‐glutamine (Sigma Aldrich) supplemented with, 1 % penicillin/streptomycin solution (Lonza), 10 % fetal bovine serum (FBS; Thermofisher Scientific) and 2 g/L sodium bicarbonate (Sigma Aldrich). The cells were maintained in a humidified environment at 37 °C and 5 % CO_2_.

For the intramacrophage assay, a 500 000 cells/mL THP‐1 cell suspension was prepared, 25 ng/mL phorbol 12‐myristate 13‐acetate (PMA) added and the suspension seeded in 96 well plates (Nunc, Thermofisher Scientific) at 200 μL/well. Cell‐free growth medium was included as the blank. The plates were incubated for 48 hours in a humidified environment at 37 °C and 5 % CO_2_ to encourage the differentiation of the suspended cells to adherent macrophages.

To eliminate non‐adherent cells after the PMA treatment, the plates were carefully washed once with warm PBS. Then, a premade promastigote suspension (200 L/well) was added. The suspension consisted of stationary phase promastigotes in THP‐1 cell growth medium, with a multiplicity of infection (MOI) of 30 : 1. Parasite‐free medium was also added to selected THP‐1 cell‐containing wells to serve as parasite blank. The plates were thoroughly rinsed four times with warm PBS to get rid of extracellular parasites after being cultured for 24 hours to encourage macrophage infection. The cells were then treated with 160 μL of growth medium and 40 μL of either solvent (parasite blank and negative control with parasites) or 40 μL of compound solutions. For a dual‐point activity screening, 10 μM and 2 mM of compound were prepared in separate plates. Amphothericin B (10 μM) served as the standard antileishmanial drug. The plates were incubated for 72 hours in a humidified environment at 37 °C and 5 % CO_2_.

For the plates treated with 10 μM of compound, any serum and extracellular promastigotes that remained were carefully removed from the wells by rinsing with warm PBS. The wells were then treated with 20 μL of 0.05 % sodium dodecyl sulphate (SDS; Sigma Aldrich) in PBS for 30 sec to lyse the macrophages. Lysis was terminated with the addition of 180 μL promastigote growth medium with 10 % FBS. For the resazurin assay, 10 μL of resazurin solution (0.025 % in PBS) was added to each well and the plates were further incubated at 37 °C in for 72 hours in the dark to measure amastigote recovery to proliferative promastigotes.

For the subsequent assay using 2 mM of compound, each well was observed for host cell viability and extracellular parasites. Any remaining extracellular promastigotes were afterwards carefully removed from the wells by rinsing with warm PBS. The wells were then treated with 50 μL of resazurin solution (0.01 % in PBS) and the plates were further incubated at 37 °C for 4 hours in the dark to assess host cell viability.

A Thermofisher Scientific's GO Multiscan plate reader was used to measure absorbance at 570 nm and 600 nm. SkanIt 4.0 Research Edition software was used to perform the data analysis for every biological replicate. Background absorbance (600 nm) was subtracted from the 570 nm absorbance data and the mean absorbance was computed.

For the activity screening, the following formula was used to determine growth inhibition percentage:

Growthinhibition%=100-[(ΔAbssample-ΔAbsblank)/(ΔAbsnegcontrol-ΔAbsblank)X100]

The cell viability effects of the compounds on the host THP‐1 cells were calculated as indicated in the cell viability assay.

## Sample Availability

Samples of the compounds are available from David. D. N'Da.

## Funding

This work was financially supported by the North‐West University, Potchefstroom campus and the National Research Foundation (NRF) (Grant no: 129324 & 148781). The funders had no role in the study design, data collection and interpretation, nor the decision to submit the work for publication.

## Author Contributions

Conceptualization: [David D. N'Da]; Methodology: [Nadine Henning, Christina Kannigadu, Janine Aucamp, Helena DJ van Rensburg, DD N'Da]; Formal analysis and investigation: [Janine Aucamp, Frank van der Kooy and David D. N'Da]; Writing ‐ original draft preparation: [Nadine Henning]; Writing ‐ review and editing: [David D. N'Da]; Funding acquisition: [David D. N'Da]; Resources: [David D. N'Da]; Supervision: [David D. N'Da].

## Conflict of Interests

The authors declare no conflict of interest. The funders had no role in the design of the study, in the collection, analyses, or interpretation of data, in the writing of the manuscript, nor in the decision to publish the results.

4

## Supporting information

As a service to our authors and readers, this journal provides supporting information supplied by the authors. Such materials are peer reviewed and may be re‐organized for online delivery, but are not copy‐edited or typeset. Technical support issues arising from supporting information (other than missing files) should be addressed to the authors.

Supporting Information

## Data Availability

The data supporting this study′s findings are provided in the supplementary information
