# Supplementary material for: Synthesis and In Vitro Antileishmanial Efficacy of Novel Ethylene Glycol Analogues of Benzothiadiazine‐1,1‐dioxide
Source: Chem Biodivers. 2024 Oct 31;22(1):e202402059. doi: 10.1002/cbdv.202402059 (PMC11741150; doi:10.1002/cbdv.202402059)
Supplement: Supplementary file 1 — Supporting Information [file CBDV-22-e202402059-s001.pdf]

Chemistry & Biodiversity

Supporting Information

Synthesis and *In Vitro* Antileishmanial Efficacy of Novel Ethylene Glycol Analogues of Benzothiadiazine-1,1-dioxide

Nadine Henning, Christina Kannigadu, Janine Aucamp, Helena D. Janse van Rensburg, Frank van der Kooy, and David D. N'Da*

Supplementary Information

Synthesis and in Vitro Antileishmanial Efficacy of Novel Ethylene Glycol Analogues of Benzothiadiazine-1,1-dioxide

Nadine Henning[a], Christina Kannigadu[a], Janine Aucamp[a], Helena D. Janse van Rensburg[a], Frank van der Kooy[a], David D. N'Da*[a]

[a]Centre of Excellence for Pharmaceutical Sciences (Pharmacen), North-West University, Potchefstroom 2520, South Africa.

*Corresponding author: David.NDa@nwu.ac.za

ORCID:

Nadine Henning: 0000-0002-6568-5754

Christina Kannigadu: 0000-0001-9486-8406

Janine Aucamp: 0000-0002-3685-5532

Helena D. Janse van Rensburg: 0000-0001-5181-9428

Frank van der Kooy: 0000-0003-2024-0485

David D. N'Da: 0000-0002-2327-0551

Table of Contents

2*H*-Benzo[*e*][1,2,4]thiadiazine-1,1-dioxide (**1**) S2

2-(2-Hydroxyethyl)-2*H*-benzo[*e*][1,2,4]thiadiazine-1,1-dioxide (**2**) S5

2-(2-Methoxyethyl)-2*H*-benzo[*e*][1,2,4]thiadiazine-1,1-dioxide (**3**) S8

2-[2-(2-Methoxyethoxy)ethyl]-2*H*-benzo[*e*][1,2,4]thiadiazine-1,1-dioxide (**4**) S11

2-{2-[2-(2-Methoxyethoxy)ethoxy]ethyl}-2*H*-benzo[*e*][1,2,4]thiadiazine-1,1-dioxide (**5**) . S14

2-(2-Ethoxyethyl)-2*H*-benzo[*e*][1,2,4]thiadiazine-1,1-dioxide (**6**) S17

2-[2-(2-Ethoxyethoxy)ethyl]-2*H*-benzo[*e*][1,2,4]thiadiazine-1,1-dioxide (**7**) S20

2-{2-[2-(2-Ethoxyethoxy)ethoxy]ethyl}-2*H*-benzo[*e*][1,2,4]thiadiazine-1,1-dioxide (**8**) S23

2-(2-Phenoxyethyl)-2*H*-benzo[*e*][1,2,4]thiadiazine-1,1-dioxide (**9**) S26

2-[2-(4-Bromophenoxy)ethyl]-2*H*-benzo[*e*][1,2,4]thiadiazine-1,1-dioxide (**10**) S29

2-[2-(4-Chlorophenoxy)ethyl]-2*H*-benzo[*e*][1,2,4]thiadiazine-1,1-dioxide (**11**) S32

2-[2-(4-Nitrophenoxy)ethyl]-2*H*-benzo[*e*][1,2,4]thiadiazine-1,1-dioxide (**12**) S35

2-[2-(Allyloxy)ethyl]-2*H*-benzo[*e*][1,2,4]thiadiazine-1,1-dioxide (**13**) S38

2-(Prop-2-yn-1-yl)-2*H*-benzo[*e*][1,2,4]thiadiazine-1,1-dioxide (**14**) S41

2*H*-Benzo[*e*][1,2,4]thiadiazine-1,1-dioxide (1)

^{1}H NMR in DMSO

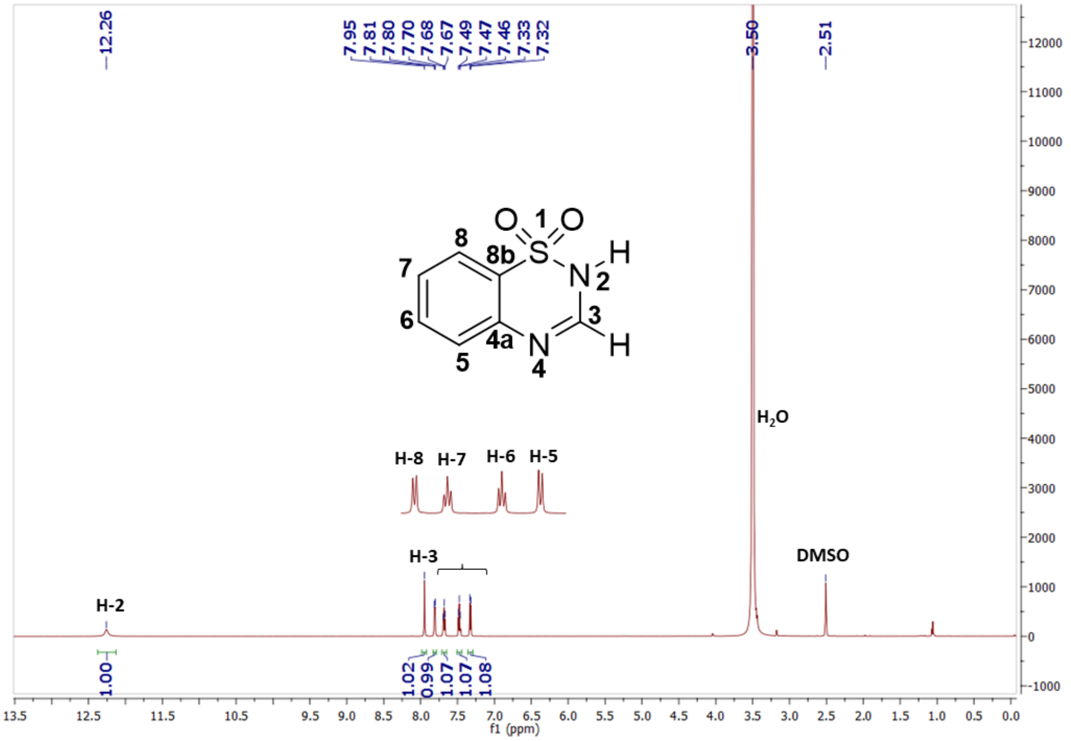

^{13}C NMR in DMSO

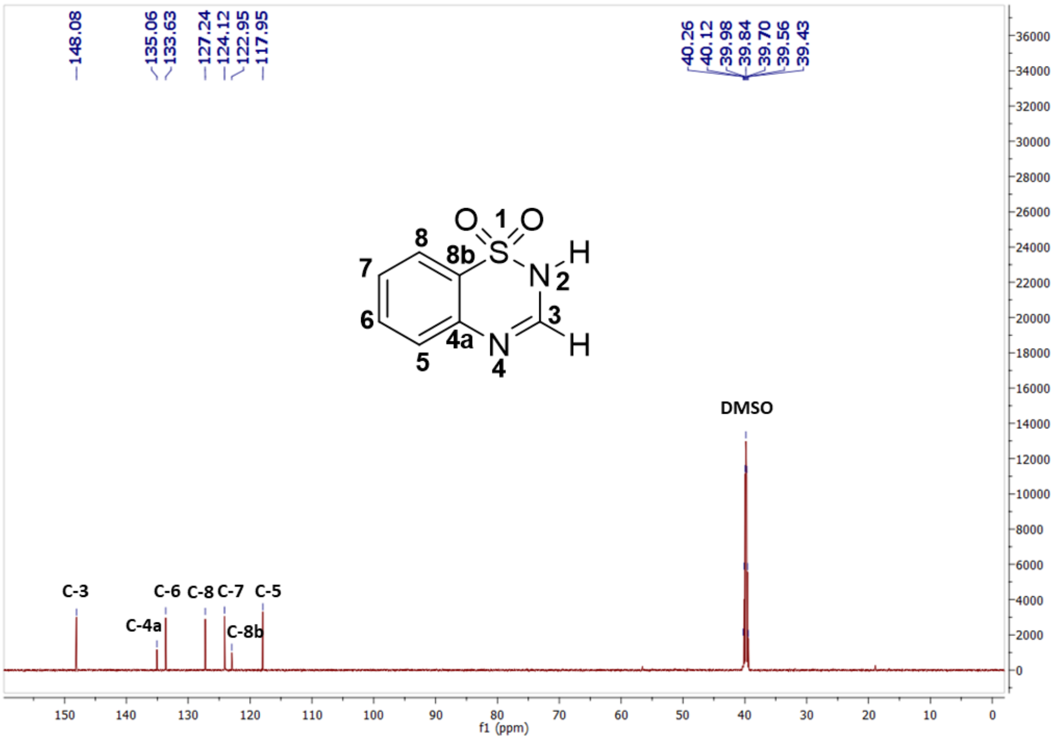

IR Spectrum

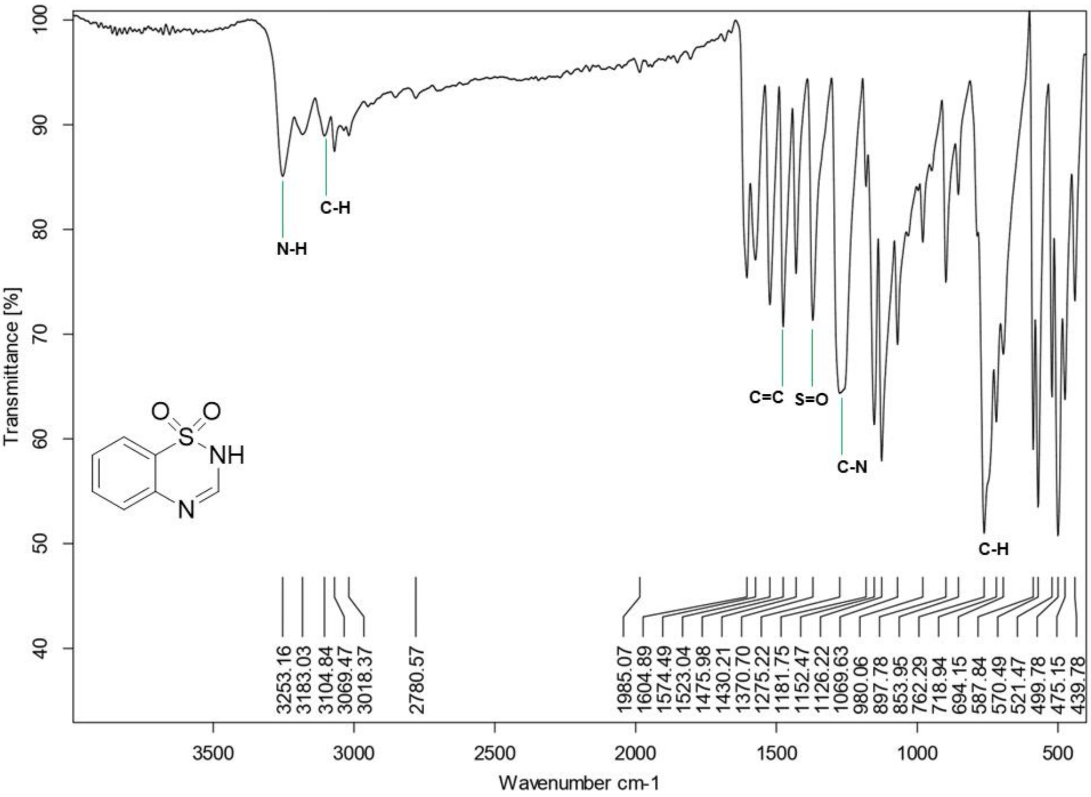

HRMS

Mass Spectrum SmartFormula Report

Analysis Info

Analysis Name	D:\Data\05102021\LADMS000015.d	Acquisition Date	10/5/2021 2:07:17 PM
Method	tune_low no focus50-1600da31052021.m	Operator	Dr JHL Jordaan
Sample Name	NH-1	Instrument / Ser#	micrOTOF-Q II 2010390
Comment			

Acquisition Parameter

Source Type	APCI	Ion Polarity	Positive	Set Nebulizer	1.8 Bar
Focus	Not active	Set Capillary	4500 V	Set Dry Heater	200 °C
Scan Begin	50 m/z	Set End Plate Offset	-500 V	Set Dry Gas	8.0 l/min
Scan End	1600 m/z	Set Collision Cell RF	150.0 Vpp	Set Divert Valve	Waste

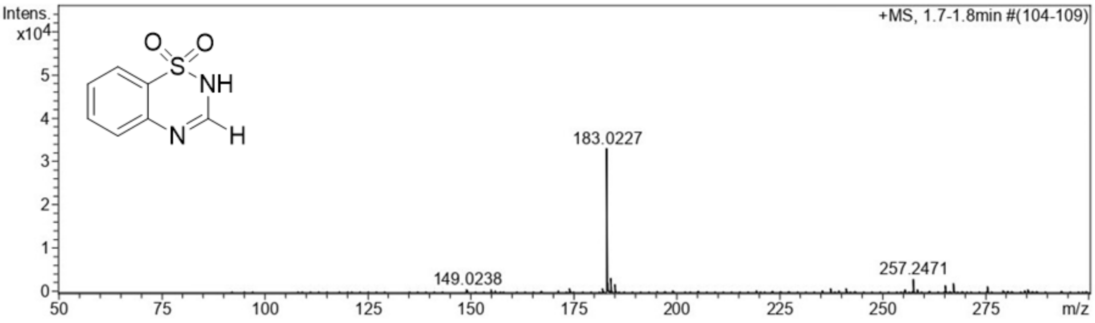

Meas. m/z	#	Formula	Score	m/z	err [mDa]	err [ppm]	mSigma	rdb	e⁻ Conf	N-Rule
183.0227	1	C 7 H 7 N 2 O 2 S	100.00	183.0223	-0.4	-2.2	6.0	5.5	even	ok

HPLC Purity

SHIMADZU LabSolutions **Analysis Report**

<Sample Information>

Sample Name	: NH-01		
Sample ID	: NH-01		
Data Filename	: NH-01_003.lcd		
Method Filename	: screening.lcm		
Batch Filename	: purity Sept 2024.lcb		
Vial #	: 1-2	Sample Type	: Unknown
Injection Volume	: 0,2 uL		
Date Acquired	: 16/09/2024 10:51:59	Acquired by	: System Administrator
Date Processed	: 16/09/2024 12:55:12	Processed by	: System Administrator

<Chromatogram>

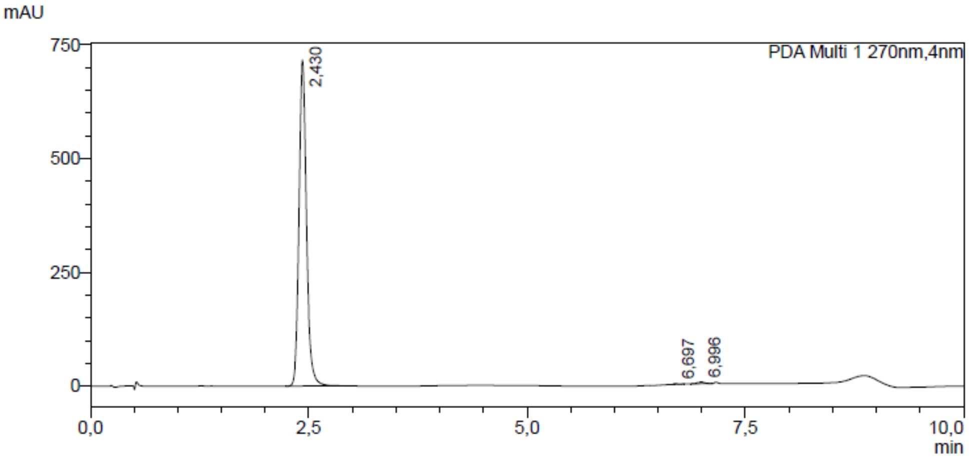

<Peak Table>

PDA Ch1 270nm

Peak#	Ret. Time	Area	Area%
1	2,430	4275689	99,408
2	6,697	5100	0,119
3	6,996	20361	0,473
Total		4301151	100,000

2-(2-Hydroxyethyl)-2*H*-benzo[*e*][1,2,4]thiadiazine-1,1-dioxide (2)

^{1}H NMR in DMSO

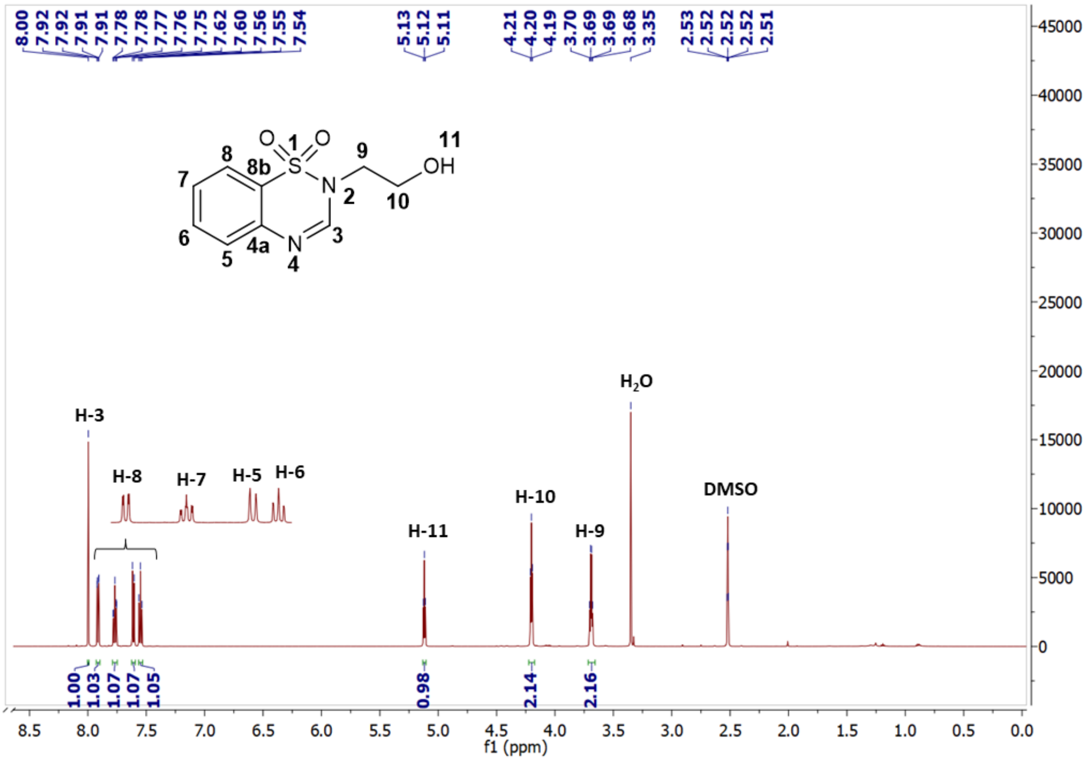

^{13}C NMR in DMSO

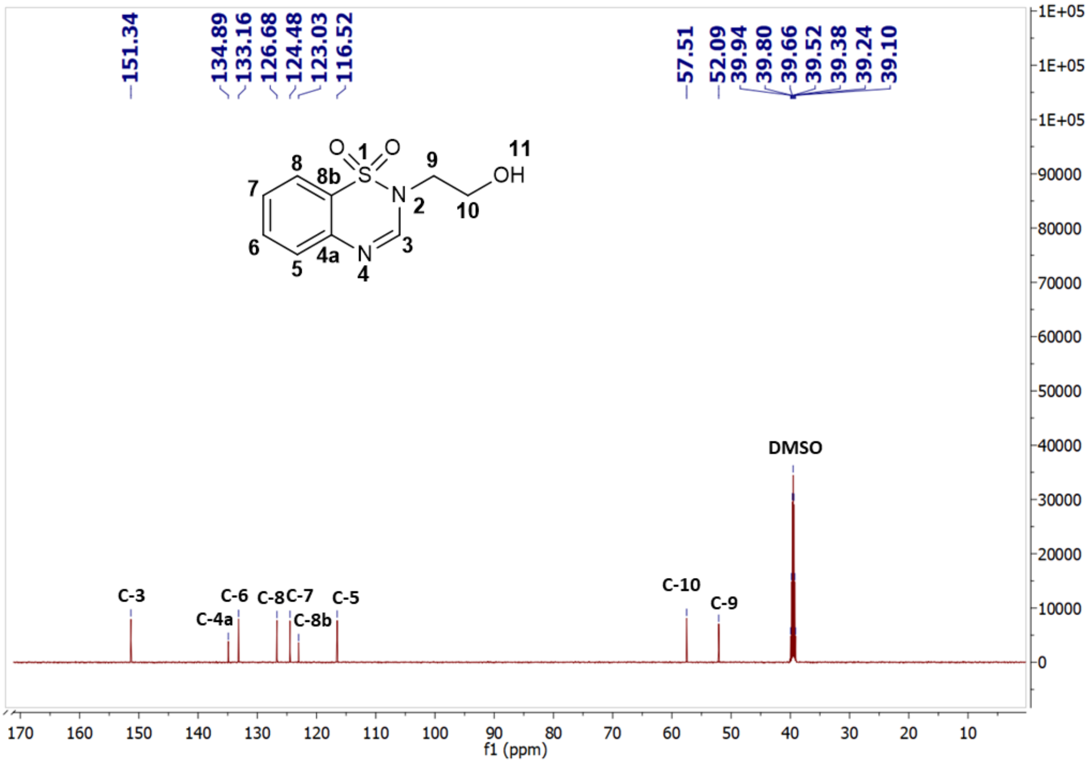

IR Spectrum

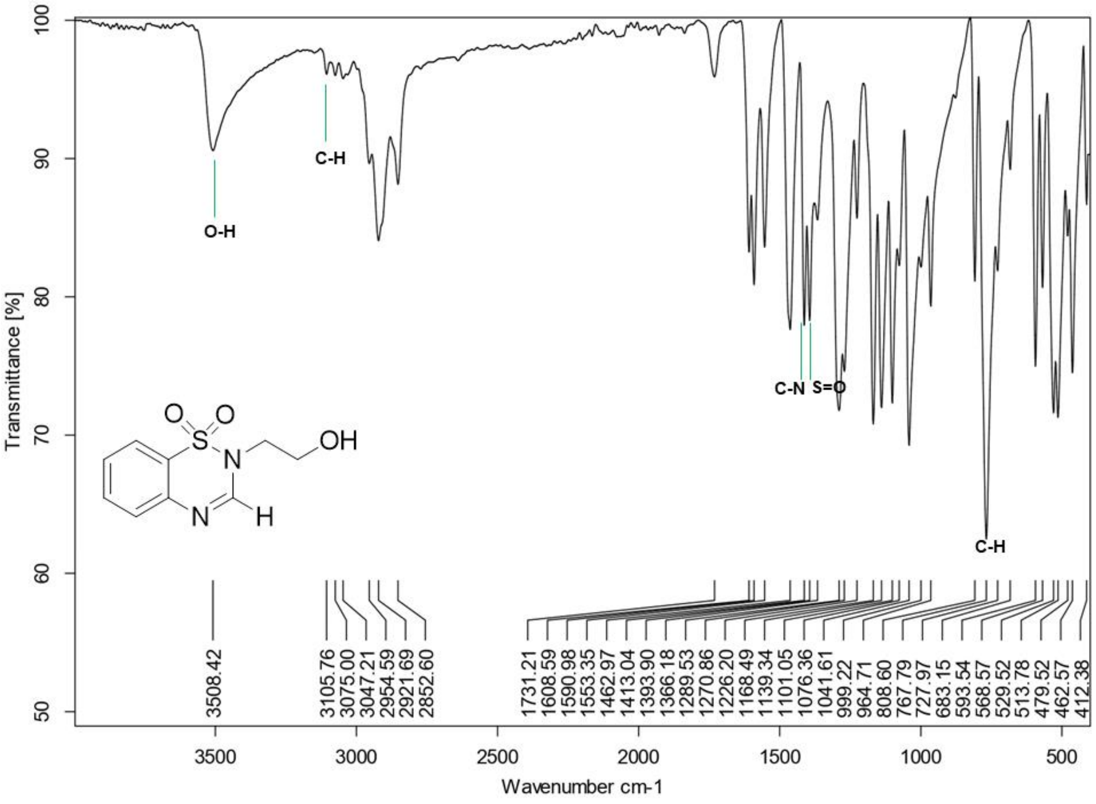

HRMS

Mass Spectrum SmartFormula Report

Analysis Info

Analysis Name	D:\Data\05102021\LADMS000013.d	Acquisition Date	10/5/2021 2:01:20 PM
Method	tune_low no focus50-1600da31052021.m	Operator	Dr JHL Jordaan
Sample Name	NH-9	Instrument / Ser#	micrOTOF-Q II 2010390
Comment			

Acquisition Parameter

Source Type	APCI	Ion Polarity	Positive	Set Nebulizer	1.8 Bar
Focus	Not active	Set Capillary	4500 V	Set Dry Heater	200 °C
Scan Begin	50 m/z	Set End Plate Offset	-500 V	Set Dry Gas	8.0 l/min
Scan End	1600 m/z	Set Collision Cell RF	150.0 Vpp	Set Divert Valve	Waste

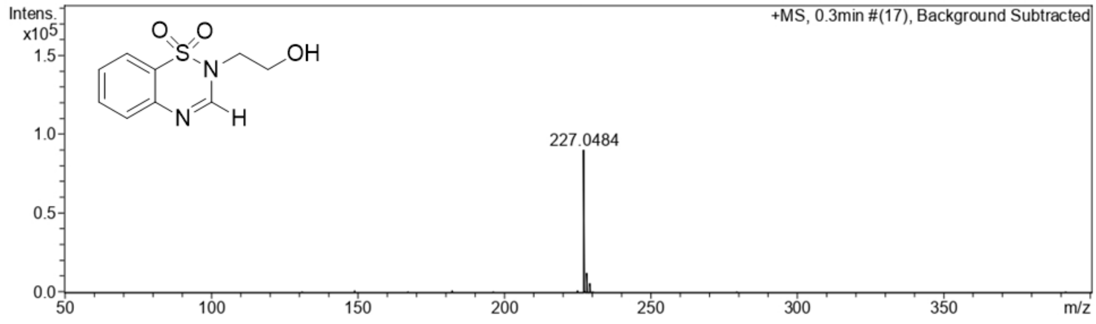

Meas. m/z	#	Formula	Score	m/z	err [mDa]	err [ppm]	mSigma	rdb	e⁻ Conf	N-Rule
227.0484	1	C 9 H 11 N 2 O 3 S	100.00	227.0485	0.0	0.2	12.6	5.5	even	ok

HPLC Purity

SHIMADZU LabSolutions **Analysis Report**

<Sample Information>

Sample Name	: NH-02		
Sample ID	: NH-02		
Data Filename	: NH-02_001.lcd		
Method Filename	: screening.lcm		
Batch Filename	: purity Sept 2024.lcb		
Vial #	: 1-3	Sample Type	: Unknown
Injection Volume	: 1 uL		
Date Acquired	: 16/09/2024 13:21:59	Acquired by	: System Administrator
Date Processed	: 16/09/2024 13:32:02	Processed by	: System Administrator

<Chromatogram>

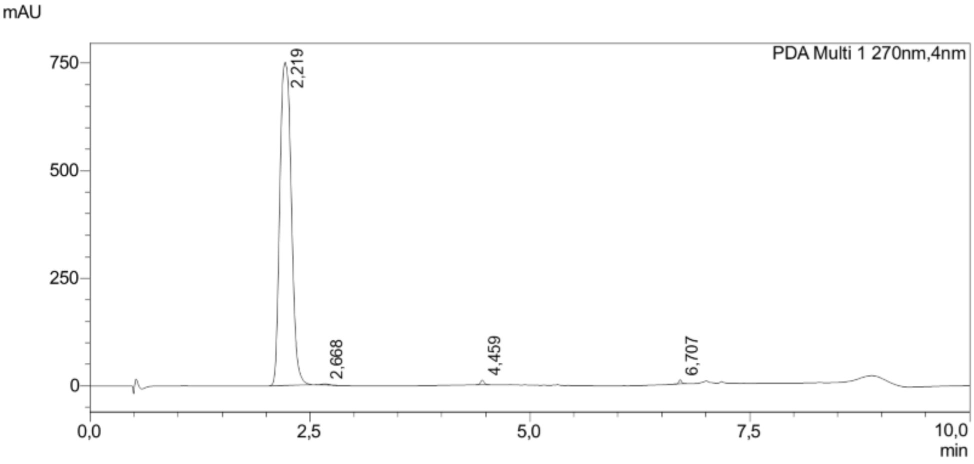

<Peak Table>

PDA Ch1 270nm

Peak#	Ret. Time	Area	Area%
1	2,219	6825613	98,951
2	2,668	18144	0,263
3	4,459	32850	0,476
4	6,707	21374	0,310
Total		6897981	100,000

2-(2-Methoxyethyl)-2*H*-benzo[*e*][1,2,4]thiadiazine-1,1-dioxide (3)

^{1}H NMR in DMSO

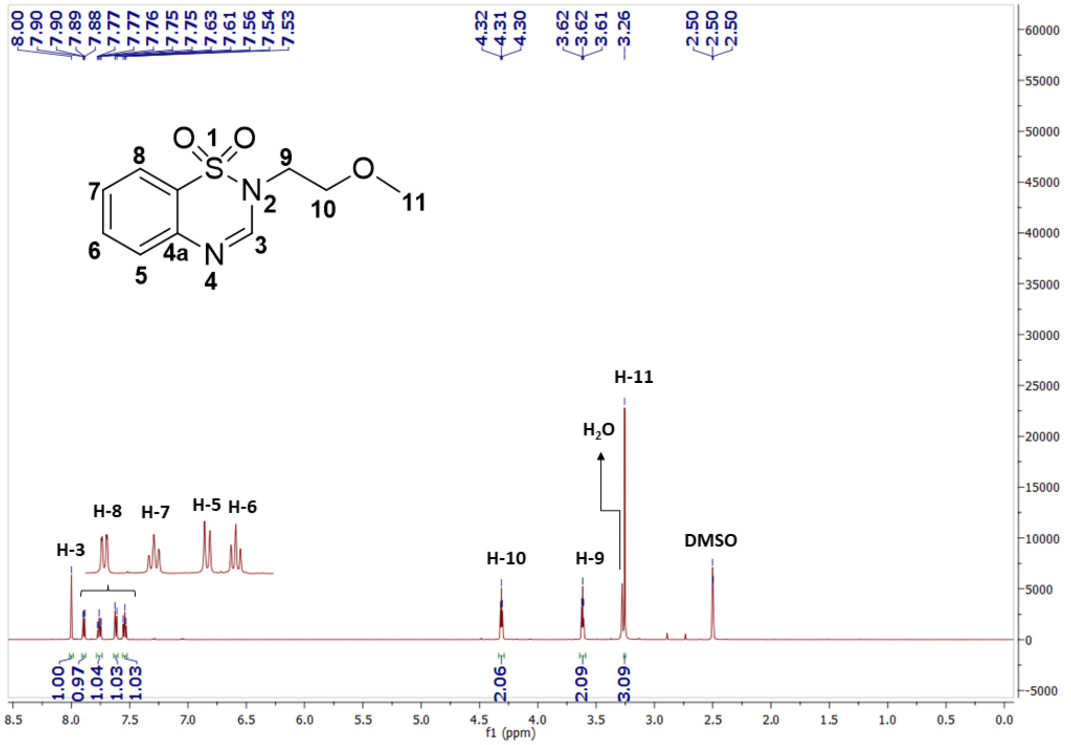

^{13}C NMR in DMSO

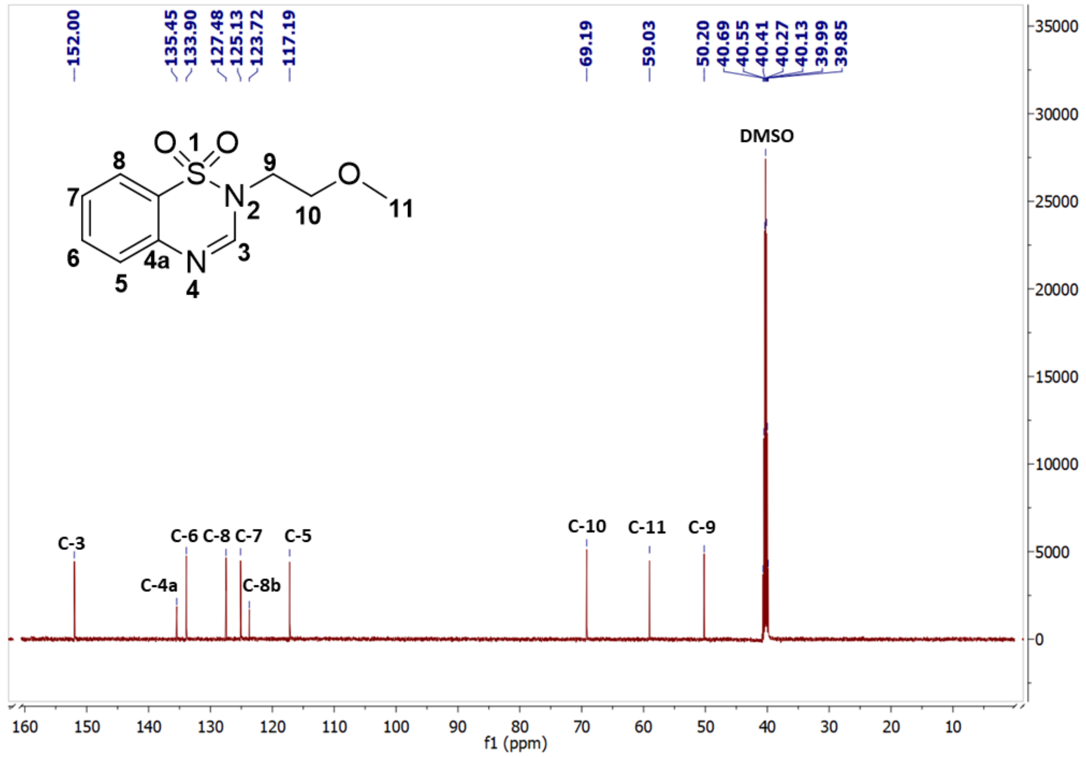

IR Spectrum

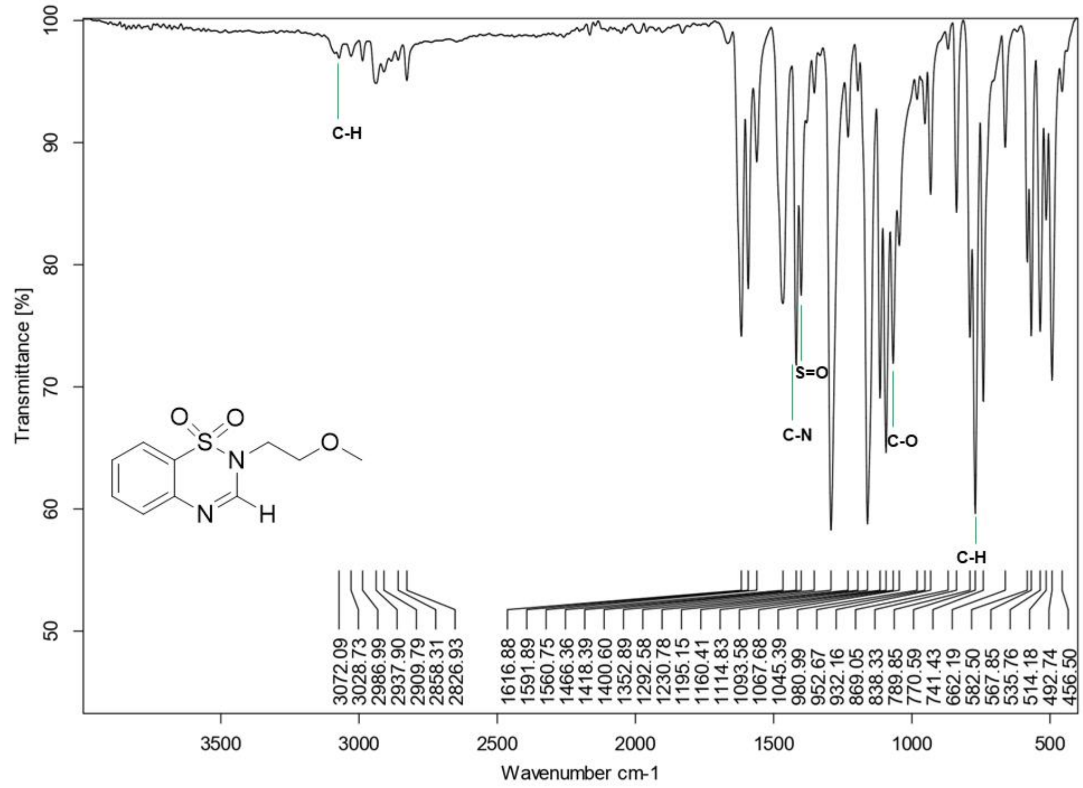

HRMS

Mass Spectrum SmartFormula Report

Analysis Info

Analysis Name	D:\Data\05102021\LADMS000011.d	Acquisition Date	10/5/2021 1:45:00 PM
Method	tune_low no focus50-1600da31052021.m	Operator	Dr JHL Jordaan
Sample Name	NH-7	Instrument / Ser#	micrOTOF-Q II 2010390
Comment			

Acquisition Parameter

Source Type	APCI	Ion Polarity	Positive	Set Nebulizer	1.8 Bar
Focus	Not active	Set Capillary	4500 V	Set Dry Heater	200 °C
Scan Begin	50 m/z	Set End Plate Offset	-500 V	Set Dry Gas	8.0 l/min
Scan End	1600 m/z	Set Collision Cell RF	150.0 Vpp	Set Divert Valve	Waste

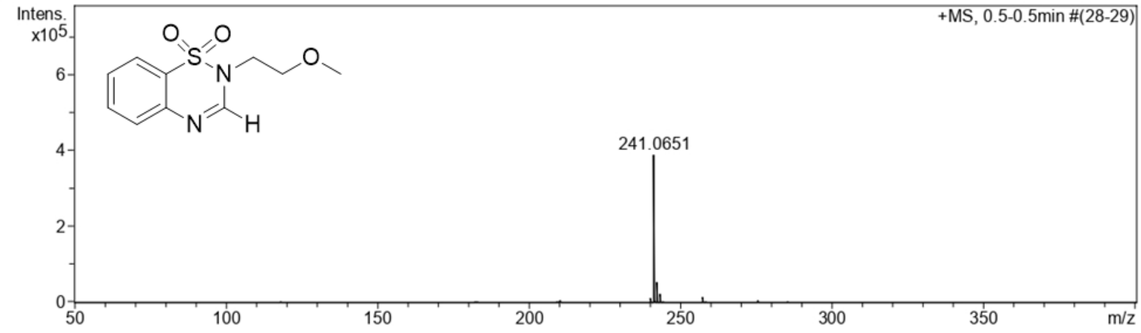

Meas. m/z	#	Formula	Score	m/z	err [mDa]	err [ppm]	mSigma	rdb	e⁻ Conf	N-Rule
241.0651	1	C 10 H 13 N 2 O 3 S	100.00	241.0641	-1.0	-4.1	5.7	5.5	even	ok

HPLC Purity

Analysis Report

SHIMADZU LabSolutions

<Sample Information>

Sample Name	: NH-03		
Sample ID	: NH-03		
Data Filename	: NH-03_005.lcd		
Method Filename	: screening.lcm		
Batch Filename	: purity Sept 2024.lcb		
Vial #	: 1-4	Sample Type	: Unknown
Injection Volume	: 0,2 uL		
Date Acquired	: 16/09/2024 11:12:45	Acquired by	: System Administrator
Date Processed	: 16/09/2024 12:39:59	Processed by	: System Administrator

<Chromatogram>

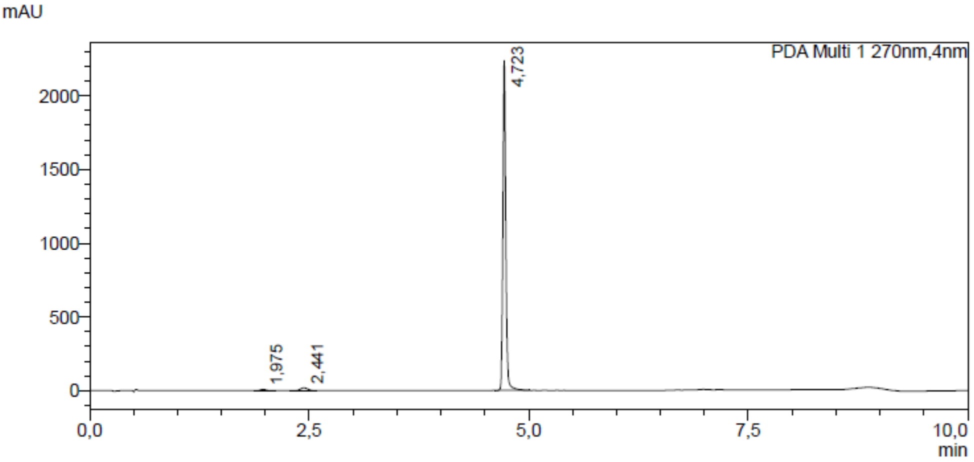

<Peak Table>

PDA Ch1 270nm

Peak#	Ret. Time	Area	Area%
1	1,975	39267	0,766
2	2,441	110854	2,164
3	4,723	4973145	97,070
Total		5123266	100,000

2-[2-(2-Methoxyethoxy)ethyl]-2*H*-benzo[*e*][1,2,4]thiadiazine-1,1-dioxide (4)

^{1}H NMR in DMSO

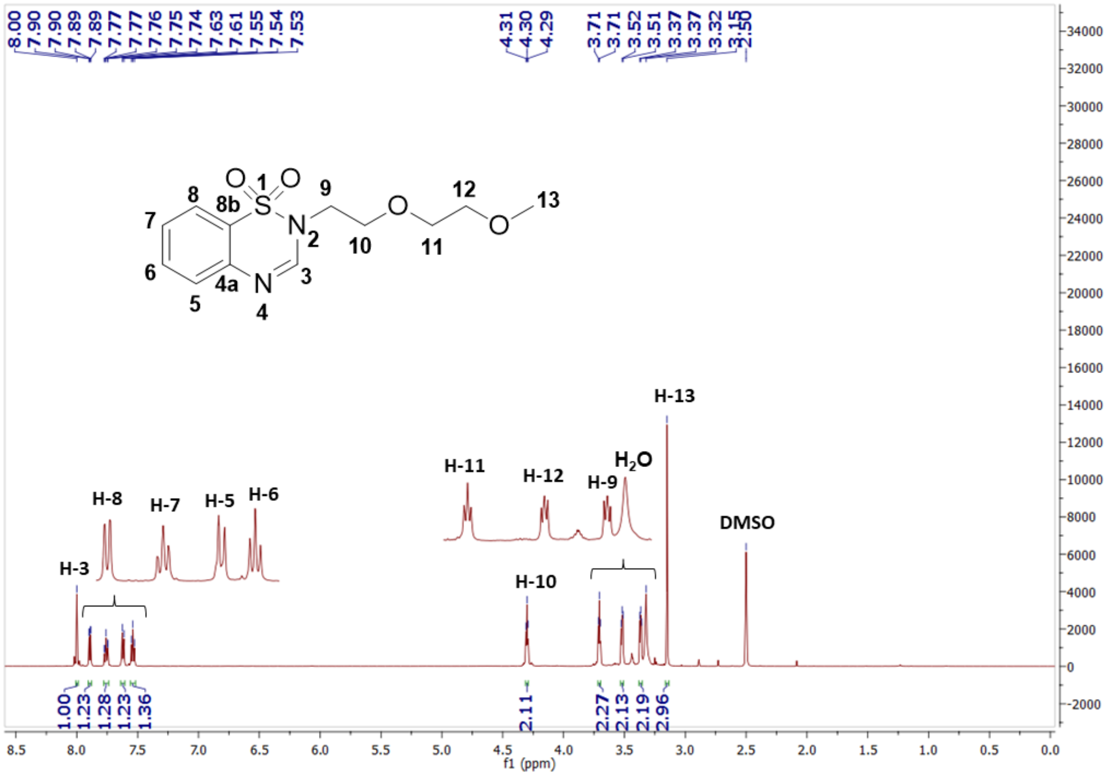

^{13}C NMR in DMSO

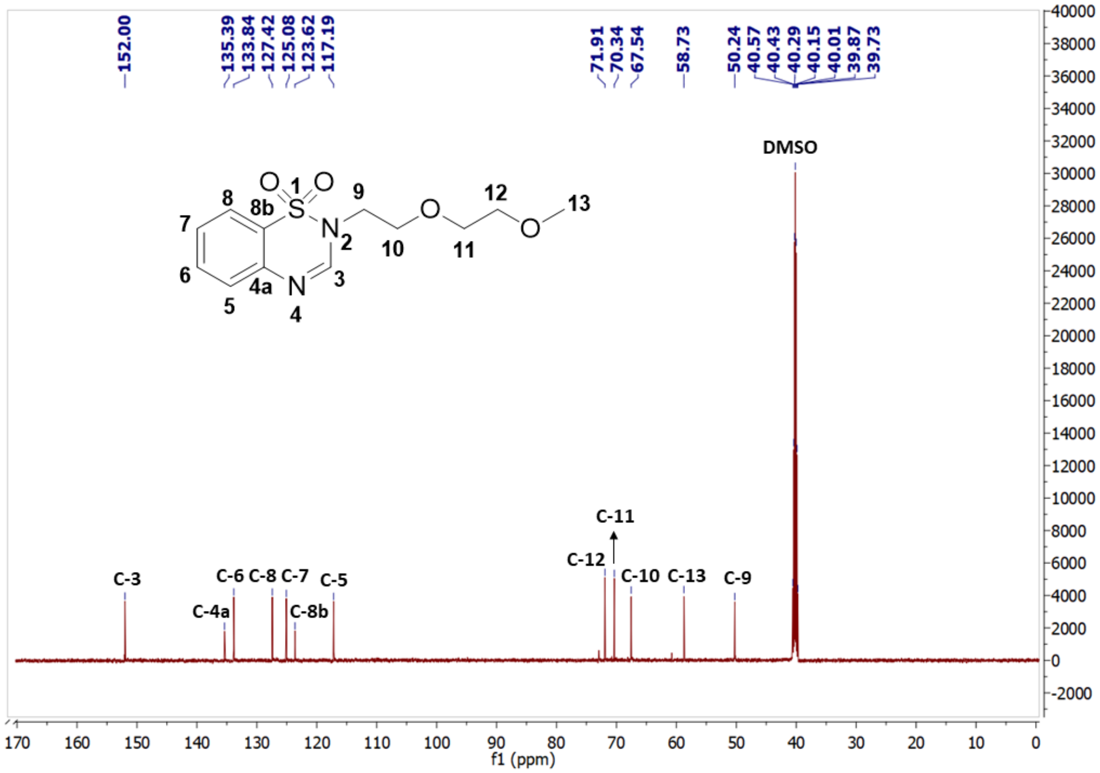

IR Spectrum

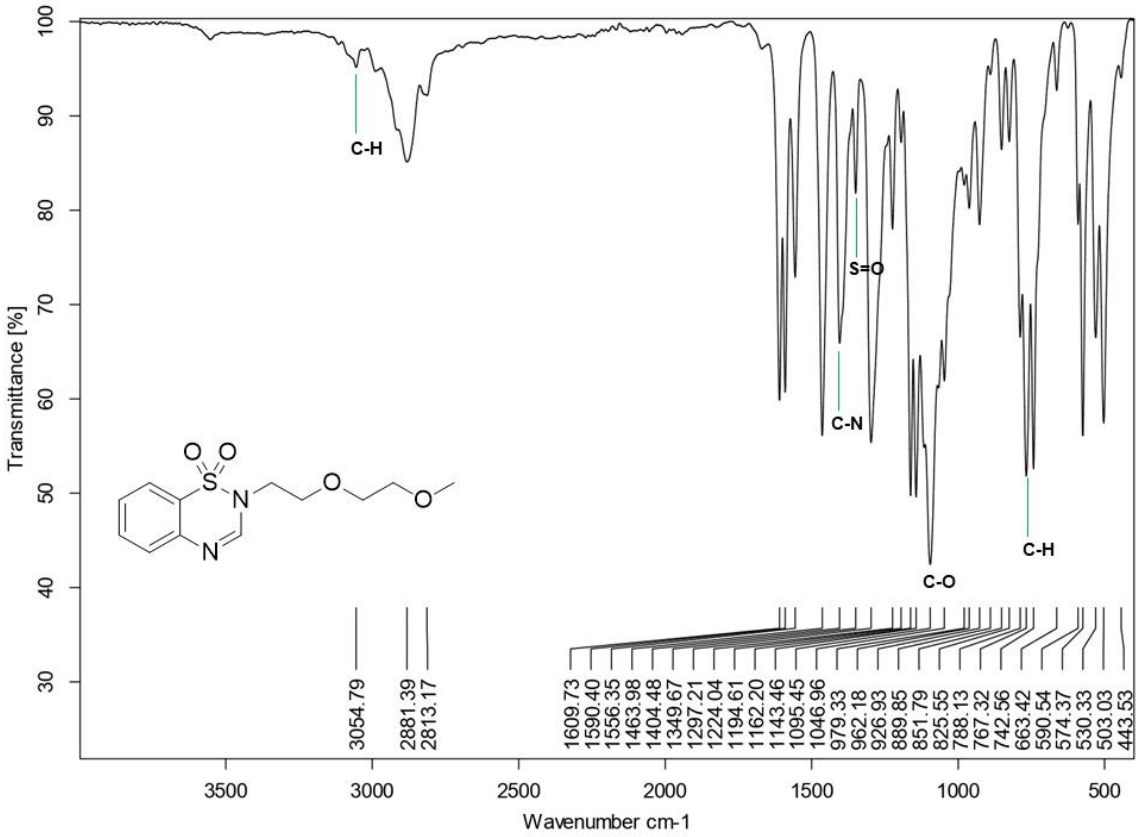

HRMS

Mass Spectrum SmartFormula Report

Analysis Info

Analysis Name	D:\Data\05102021\LADMS000010.d	Acquisition Date	10/5/2021 1:39:51 PM
Method	tune_low no focus50-1600da31052021.m	Operator	Dr JHL Jordaan
Sample Name	NH-6	Instrument / Ser#	micrOTOF-Q II 2010390
Comment			

Acquisition Parameter

Source Type	APCI	Ion Polarity	Positive	Set Nebulizer	1.8 Bar
Focus	Not active	Set Capillary	4500 V	Set Dry Heater	200 °C
Scan Begin	50 m/z	Set End Plate Offset	-500 V	Set Dry Gas	8.0 l/min
Scan End	1600 m/z	Set Collision Cell RF	150.0 Vpp	Set Divert Valve	Waste

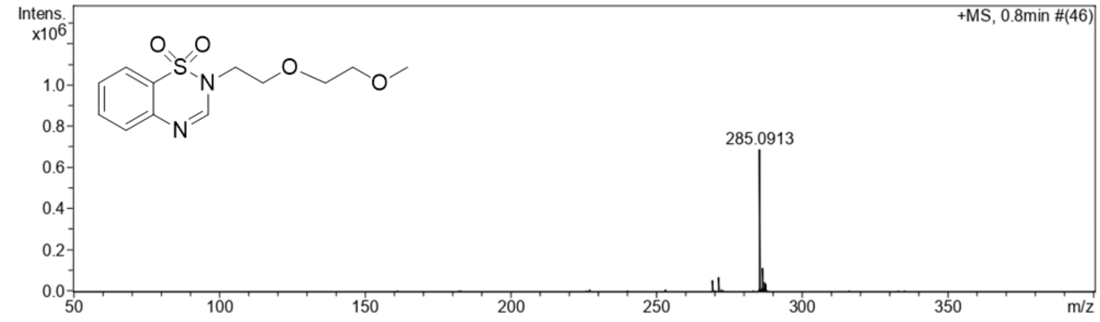

Meas. m/z	#	Formula	Score	m/z	err [mDa]	err [ppm]	mSigma	rdb	e⁻ Conf	N-Rule
285.0913	1	C 12 H 17 N 2 O 4 S	100.00	285.0904	-1.0	-3.4	11.2	5.5	even	ok

HPLC Purity

SHIMADZU LabSolutions **Analysis Report**

<Sample Information>

Sample Name	: NH-04		
Sample ID	: NH-04		
Data Filename	: NH-04_006.lcd		
Method Filename	: screening.lcm		
Batch Filename	: purity Sept 2024.lcb		
Vial #	: 1-5	Sample Type	: Unknown
Injection Volume	: 0,2 uL		
Date Acquired	: 16/09/2024 11:23:08	Acquired by	: System Administrator
Date Processed	: 16/09/2024 11:58:41	Processed by	: System Administrator

<Chromatogram>

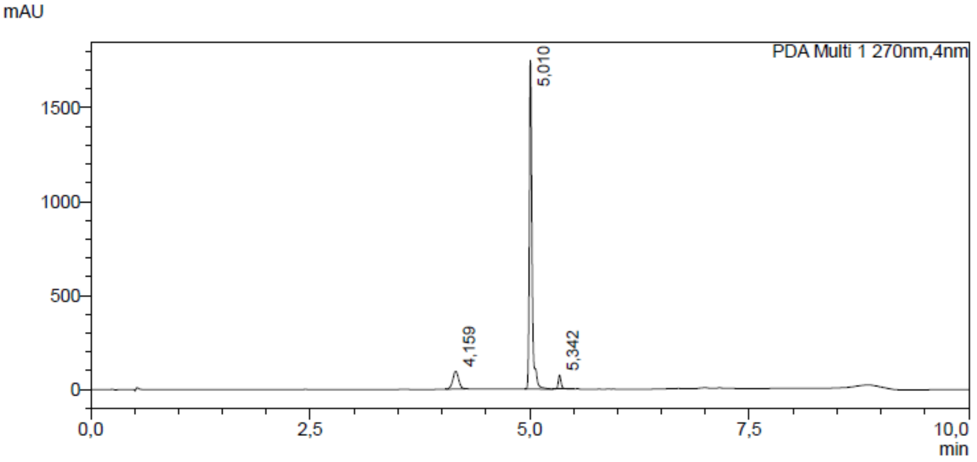

<Peak Table>

PDA Ch1 270nm

Peak#	Ret. Time	Area	Area%
1	4,159	434961	9,899
2	5,010	3803595	86,559
3	5,342	155646	3,542
Total		4394201	100,000

2-{2-[2-(2-Methoxyethoxy)ethoxy]ethyl}-2*H*-benzo[*e*][1,2,4]thiadiazine-1,1-dioxide (5)

^{1}H NMR in DMSO

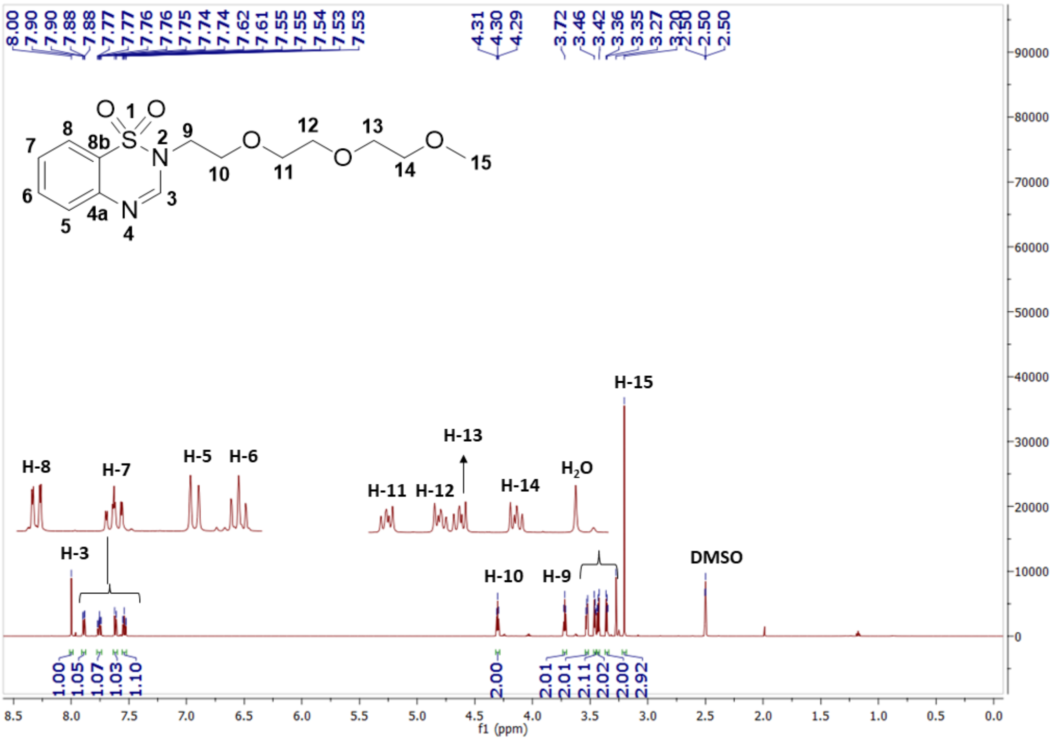

^{13}C NMR in DMSO

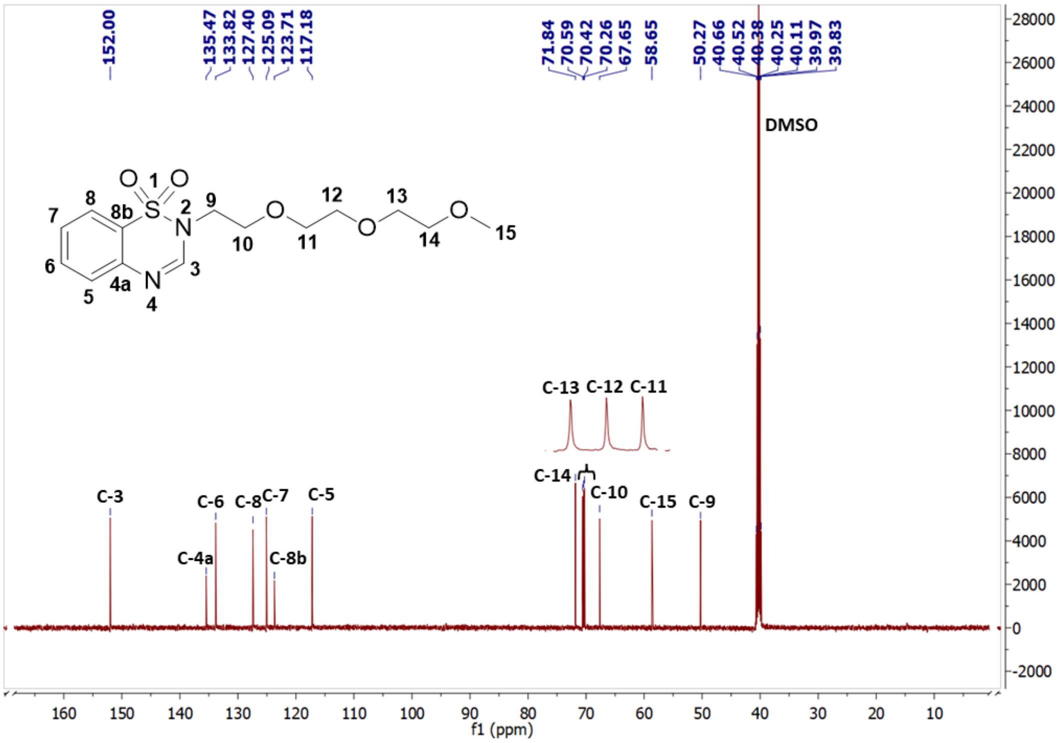

IR Spectrum

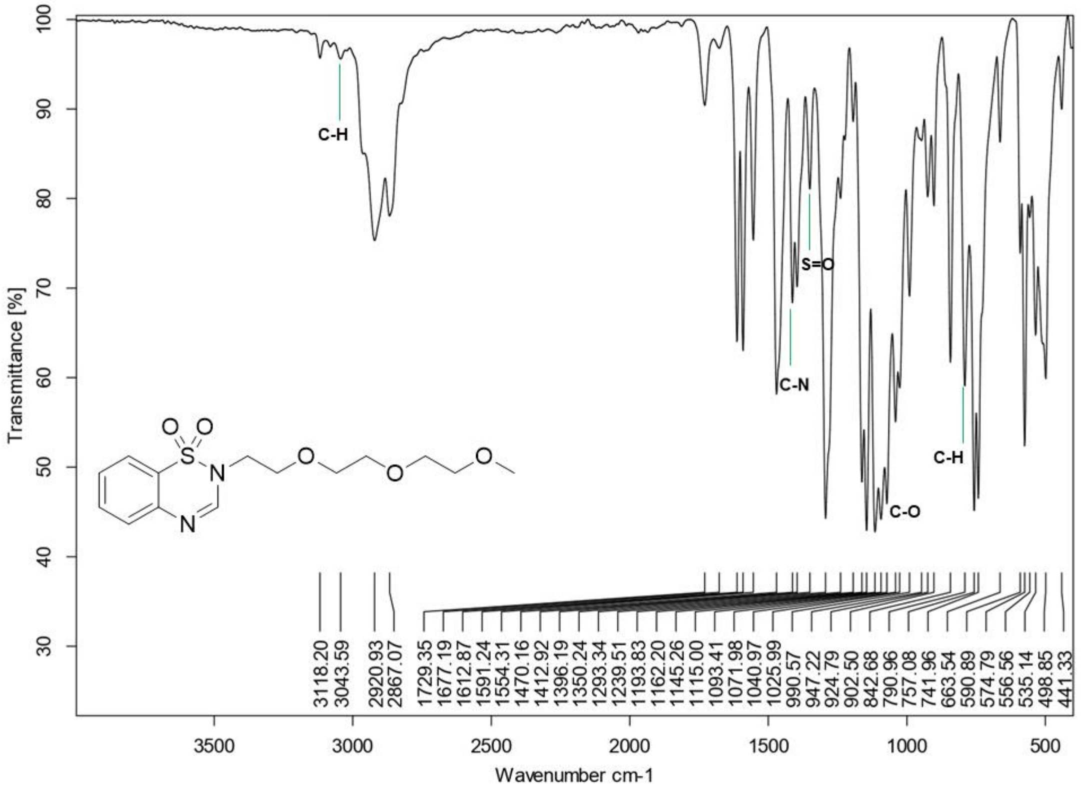

HRMS

Mass Spectrum SmartFormula Report

Analysis Info

Analysis Name	D:\Data\05102021\LADMS000007.d	Acquisition Date	10/5/2021 1:33:55 PM
Method	tune_low no focus50-1600da31052021.m	Operator	Dr JHL Jordaan
Sample Name	NH-3	Instrument / Ser#	micrOTOF-Q II 2010390
Comment			

Acquisition Parameter

Source Type	APCI	Ion Polarity	Positive	Set Nebulizer	1.8 Bar
Focus	Not active	Set Capillary	4500 V	Set Dry Heater	200 °C
Scan Begin	50 m/z	Set End Plate Offset	-500 V	Set Dry Gas	8.0 l/min
Scan End	1600 m/z	Set Collision Cell RF	150.0 Vpp	Set Divert Valve	Waste

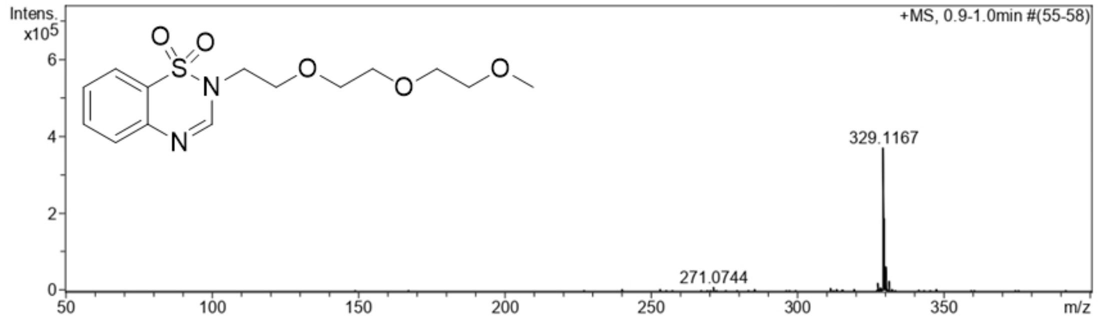

Meas. m/z	#	Formula	Score	m/z	err [mDa]	err [ppm]	mSigma	rdb	e¯ Conf	N-Rule
329.1167	1	C 14 H 21 N 2 O 5 S	100.00	329.1166	-0.1	-0.4	1.4	5.5	even	ok

HPLC Purity

SHIMADZU LabSolutions

Analysis Report

<Sample Information>

Sample Name	: NH-05		
Sample ID	: NH-05		
Data Filename	: NH-05_007.lcd		
Method Filename	: screening.lcm		
Batch Filename	: purity Sept 2024.lcb		
Vial #	: 1-6	Sample Type	: Unknown
Injection Volume	: 0,2 uL		
Date Acquired	: 16/09/2024 11:33:31	Acquired by	: System Administrator
Date Processed	: 16/09/2024 11:43:33	Processed by	: System Administrator

<Chromatogram>

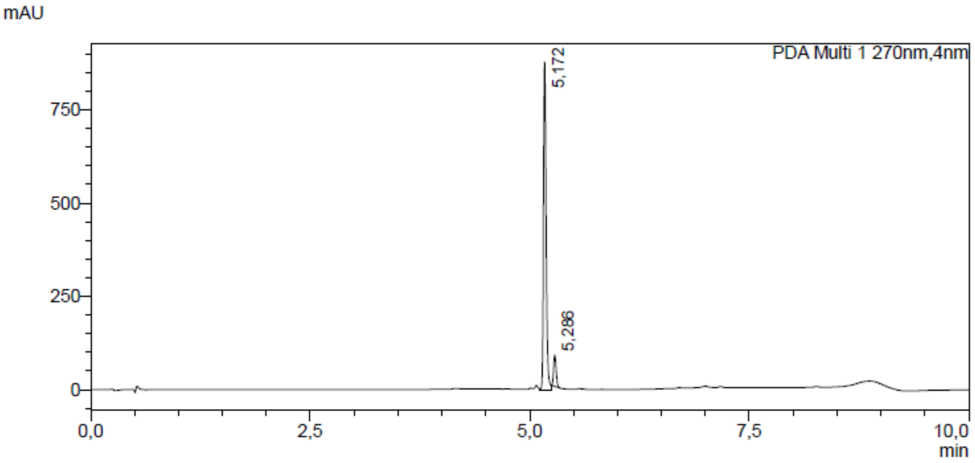

<Peak Table>

PDA Ch1 270nm

Peak#	Ret. Time	Area	Area%
1	5,172	1836155	91,825
2	5,286	163466	8,175
Total		1999621	100,000

2-(2-Ethoxyethyl)-2*H*-benzo[*e*][1,2,4]thiadiazine-1,1-dioxide (6)

^{1}H NMR in DMSO

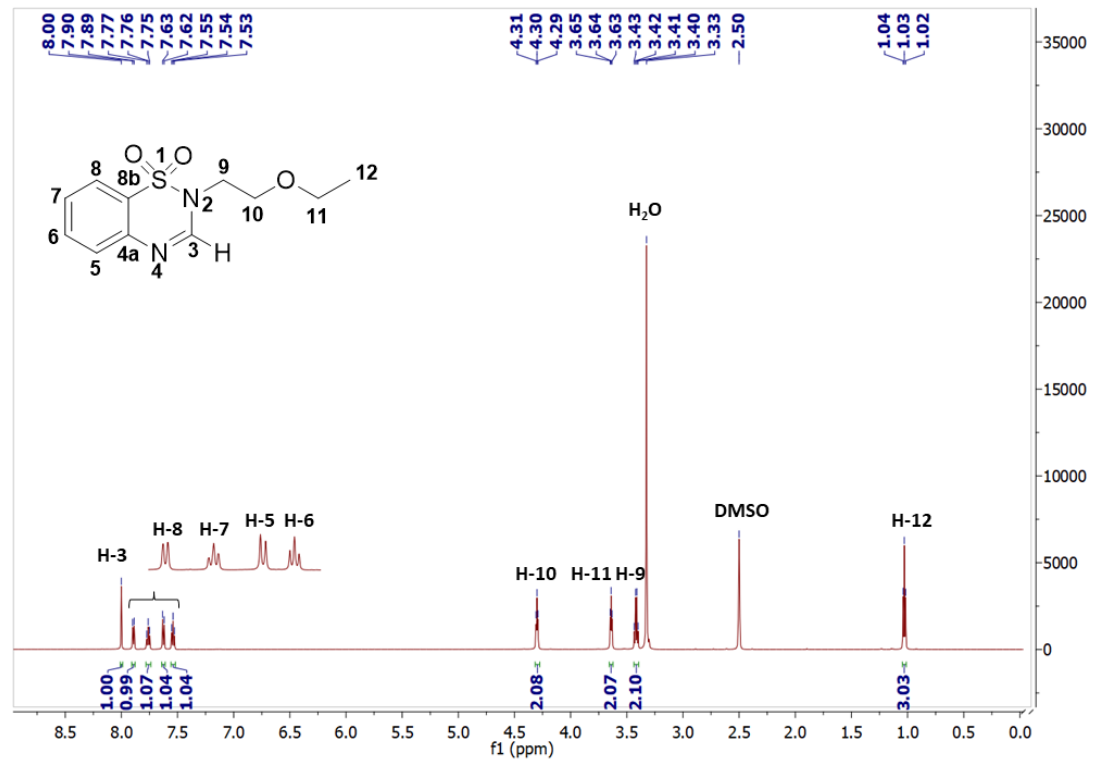

^{13}C NMR in DMSO

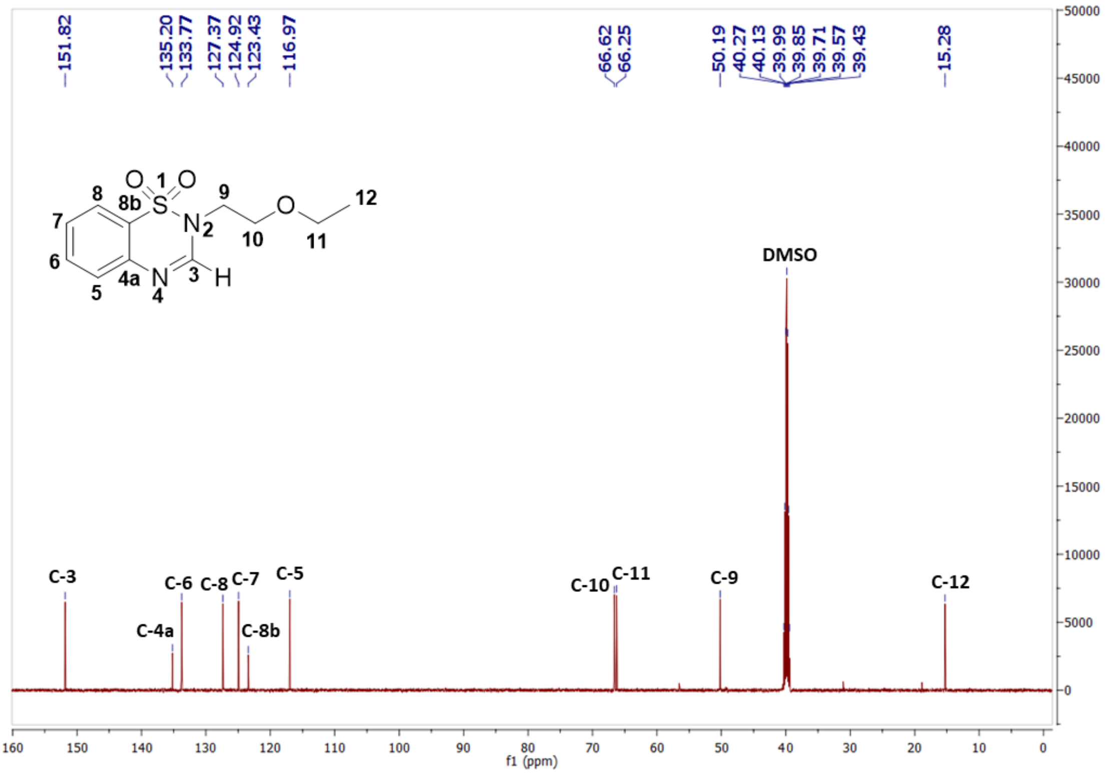

IR Spectrum

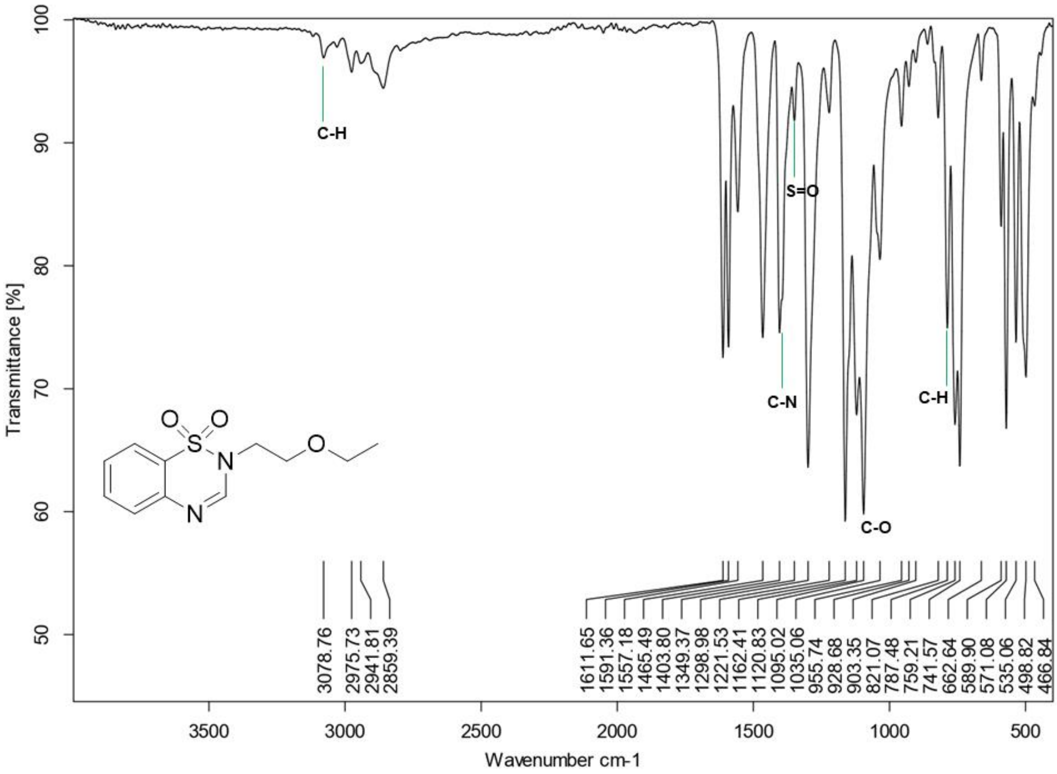

HRMS

Mass Spectrum SmartFormula Report

Analysis Info

Analysis Name	D:\Data\05102021\LADMS000008.d	Acquisition Date	10/5/2021 1:36:45 PM
Method	tune_low no focus50-1600da31052021.m	Operator	Dr JHL Jordaan
Sample Name	NH-4	Instrument / Ser#	micrOTOF-Q II 2010390
Comment			

Acquisition Parameter

Source Type	APCI	Ion Polarity	Positive	Set Nebulizer	1.8 Bar
Focus	Not active	Set Capillary	4500 V	Set Dry Heater	200 °C
Scan Begin	50 m/z	Set End Plate Offset	-500 V	Set Dry Gas	8.0 l/min
Scan End	1600 m/z	Set Collision Cell RF	150.0 Vpp	Set Divert Valve	Waste

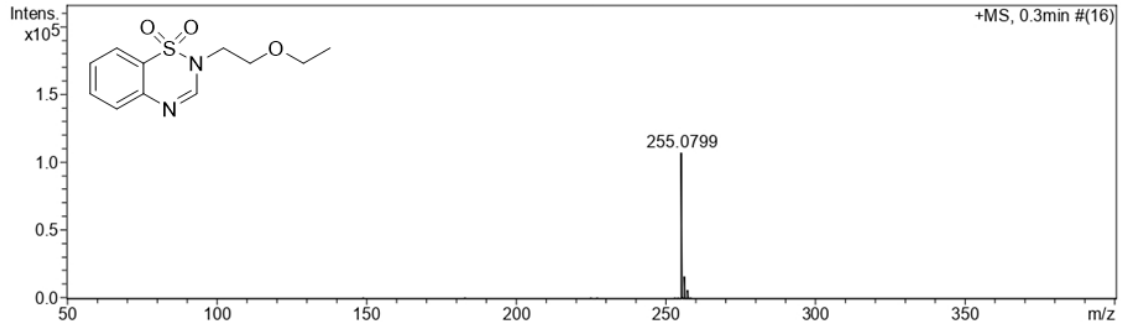

Meas. m/z	#	Formula	Score	m/z	err [mDa]	err [ppm]	mSigma	rdb	e⁻ Conf	N-Rule
255.0799	1	C 11 H 15 N 2 O 3 S	100.00	255.0798	-0.1	-0.3	8.0	5.5	even	ok

HPLC Purity

Analysis Report

<Sample Information>

Sample Name : NH-06
Sample ID : NH-06
Data Filename : NH-06_008.lcd
Method Filename : screening.lcm
Batch Filename : purity Sept 2024.lcb
Vial # : 1-7
Injection Volume : 0,2 uL
Date Acquired : 16/09/2024 11:43:56
Date Processed : 16/09/2024 11:53:57

Sample Type : Unknown
Acquired by : System Administrator
Processed by : System Administrator

<Chromatogram>

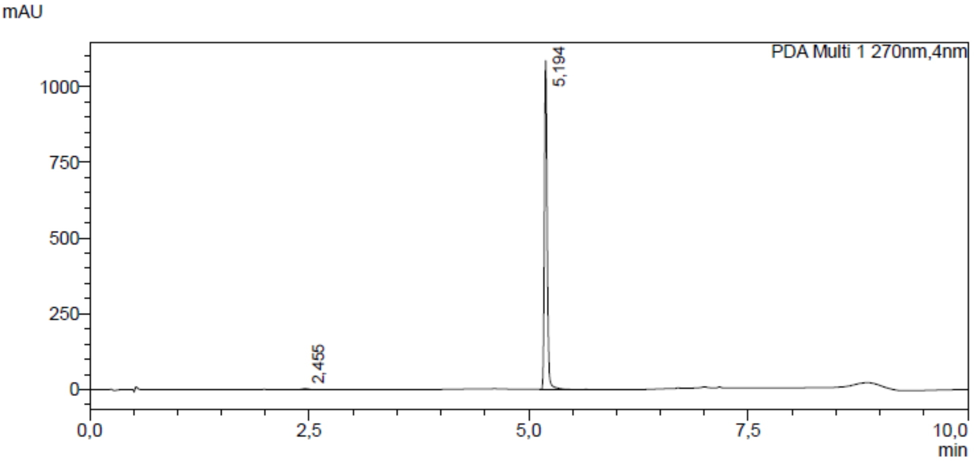

<Peak Table>

PDA Ch1 270nm

Peak#	Ret. Time	Area	Area%
1	2,455	13802	0,587
2	5,194	2337494	99,413
Total		2351296	100,000

2-[2-(2-Ethoxyethoxy)ethyl]-2*H*-benzo[*e*][1,2,4]thiadiazine-1,1-dioxide (7)

^{1}H NMR in DMSO

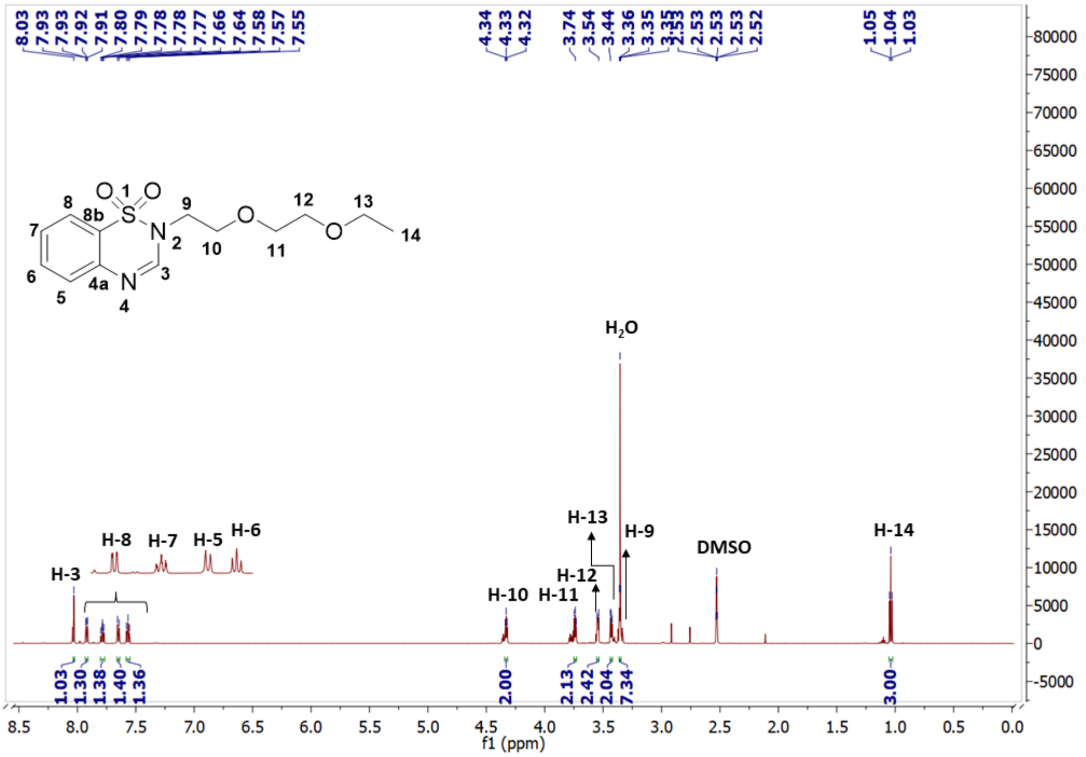

^{13}C NMR in DMSO

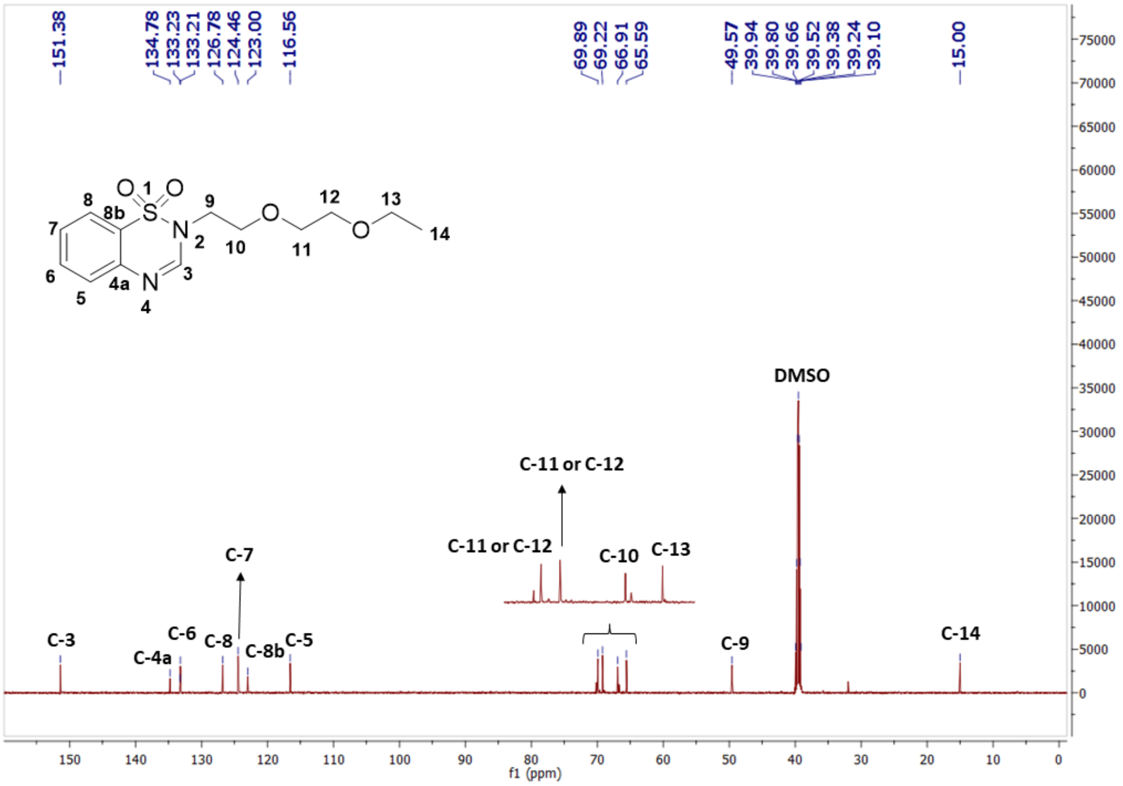

IR Spectrum

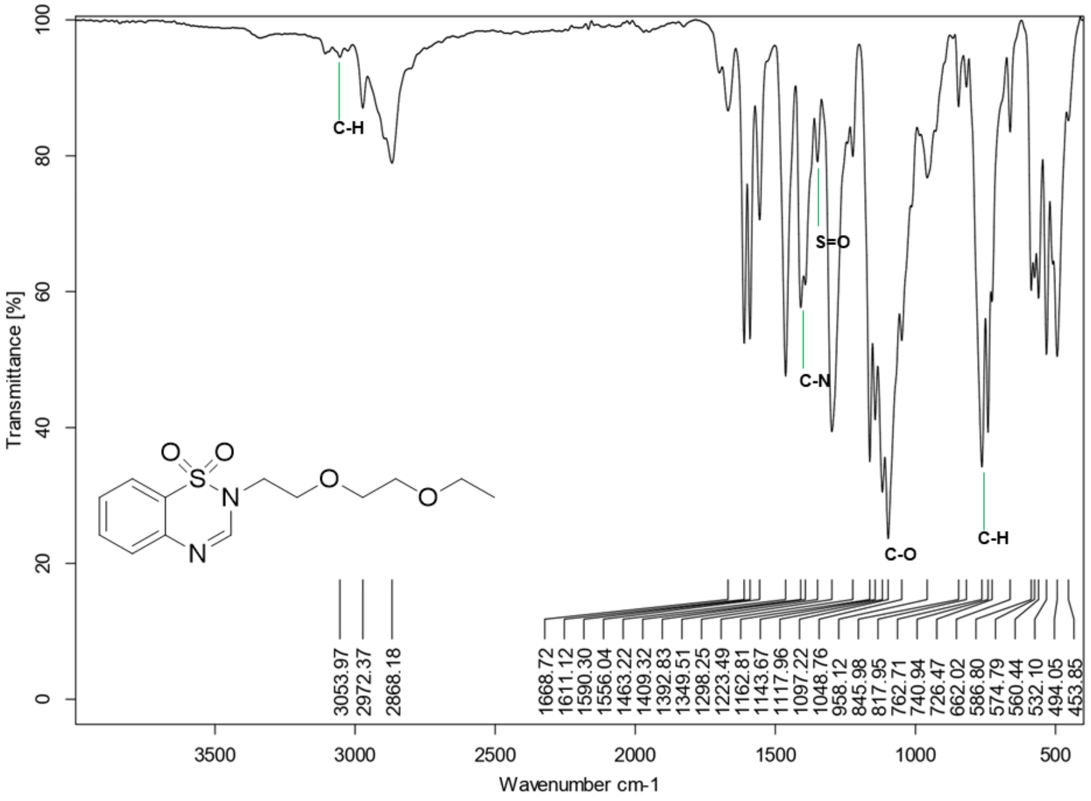

HRMS

Mass Spectrum SmartFormula Report

Analysis Info

Analysis Name	D:\Data\18112021\LADMS000002.d	Acquisition Date	11/18/2021 8:41:28 AM
Method	tune_low no focus50-1600da31052021.m	Operator	Dr JHL Jordaan
Sample Name	NH-11	Instrument / Ser#	micrOTOF-Q II 2010390
Comment			

Acquisition Parameter

Source Type	APCI	Ion Polarity	Positive	Set Nebulizer	1.8 Bar
Focus	Not active	Set Capillary	4500 V	Set Dry Heater	200 °C
Scan Begin	50 m/z	Set End Plate Offset	-500 V	Set Dry Gas	4.0 l/min
Scan End	1600 m/z	Set Collision Cell RF	150.0 Vpp	Set Divert Valve	Waste

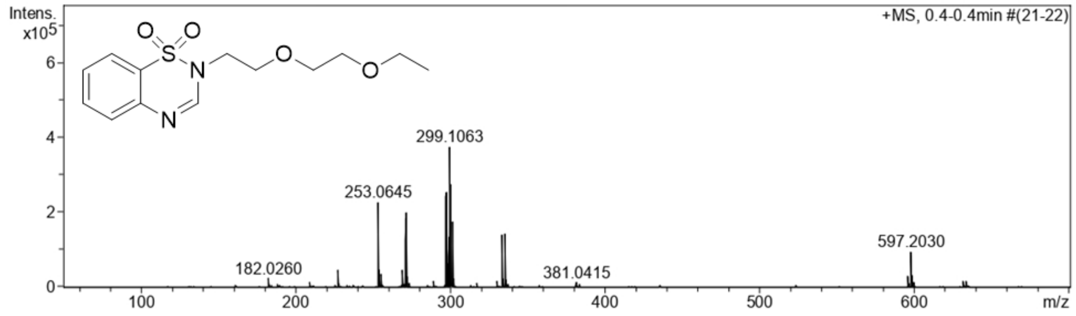

Meas. m/z	#	Formula	Score	m/z	err [mDa]	err [ppm]	mSigma	rdb	e⁻ Conf	N-Rule
299.1063	1	C 13 H 19 N 2 O 4 S	100.00	299.1060	-0.3	-0.9	355.0	5.5	even	ok

HPLC Purity

SHIMADZU LabSolutions

Analysis Report

<Sample Information>

Sample Name : NH-07
Sample ID : NH-07
Data Filename : NH-07_009.lcd
Method Filename : screening.lcm
Batch Filename : purity Sept 2024.lcb
Vial # : 1-8
Injection Volume : 0,2 uL
Date Acquired : 16/09/2024 11:54:20
Date Processed : 16/09/2024 12:48:13

Sample Type : Unknown
Acquired by : System Administrator
Processed by : System Administrator

<Chromatogram>

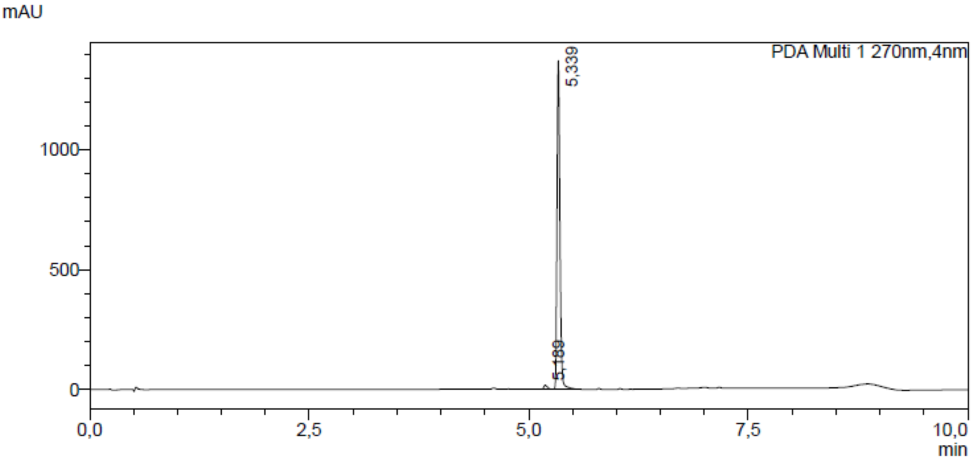

<Peak Table>

PDA Ch1 270nm

Peak#	Ret. Time	Area	Area%
1	5,189	48583	1,529
2	5,339	3128918	98,471
Total		3177501	100,000

2-{2-[2-(2-Ethoxyethoxy)ethoxy]ethyl}-2*H*-benzo[*e*][1,2,4]thiadiazine-1,1-dioxide (8)

^{1}H NMR in DMSO

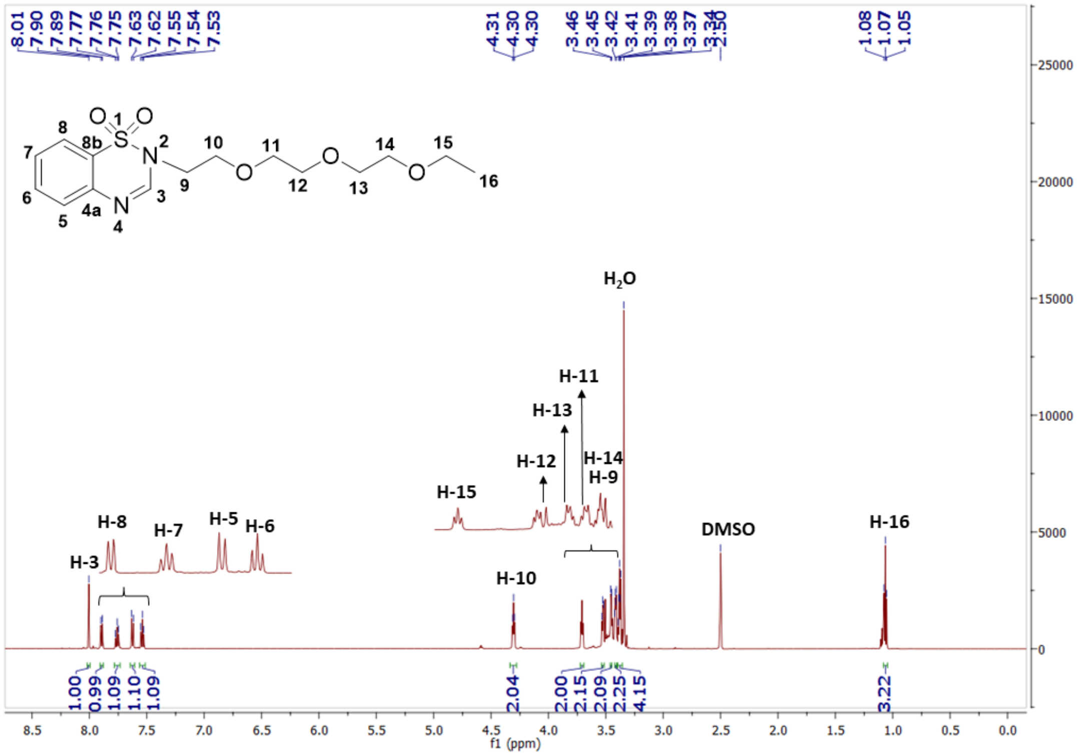

^{13}C NMR in DMSO

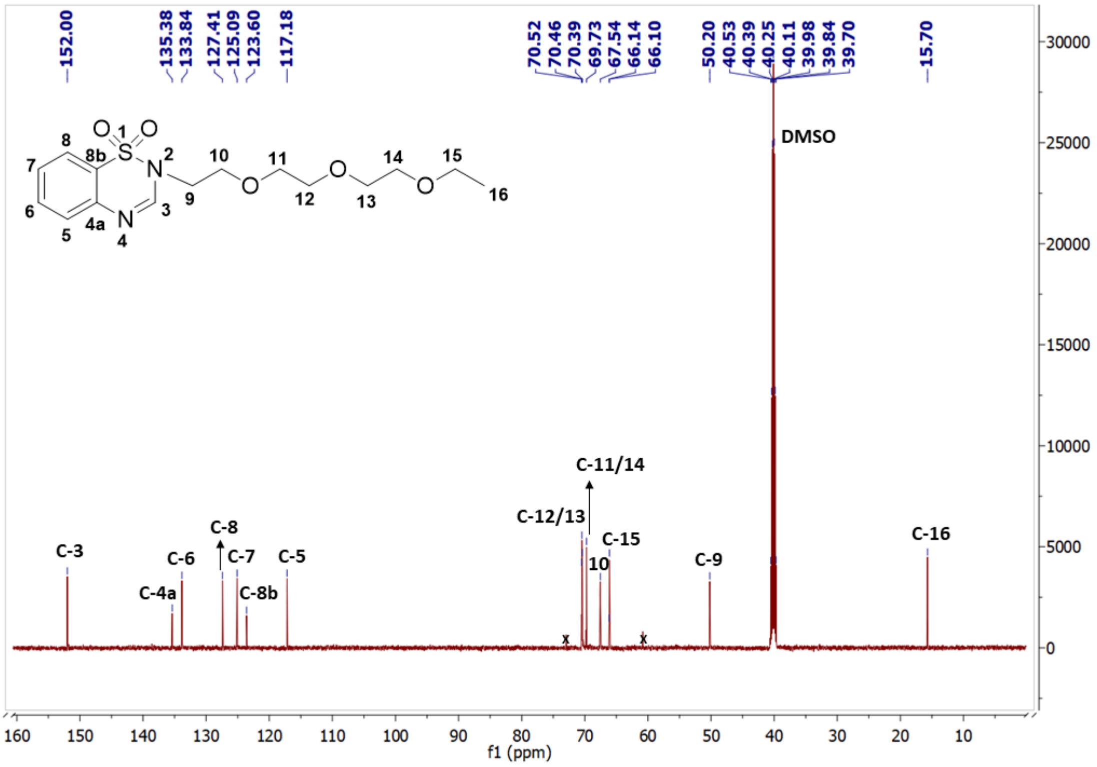

IR Spectrum

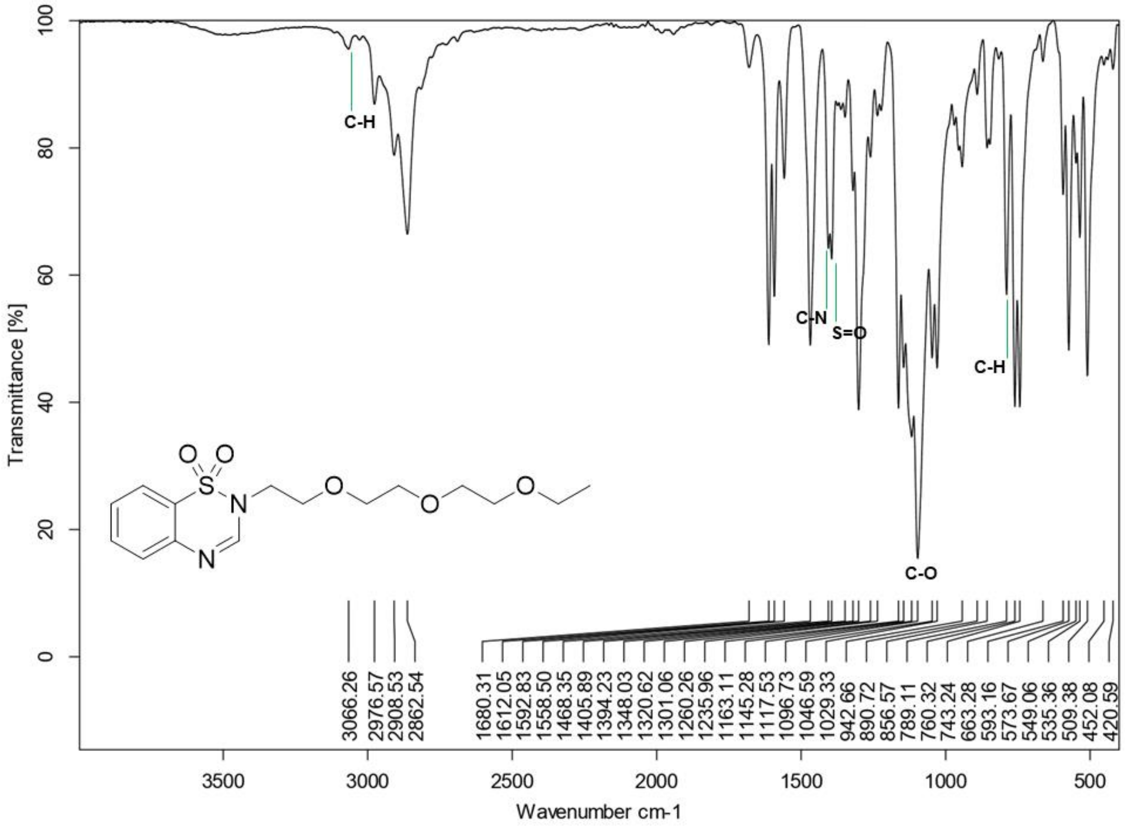

HRMS

Mass Spectrum SmartFormula Report

Analysis Info

Analysis Name	D:\Data\18112021\LADMS000005.d	Acquisition Date	11/18/2021 8:49:09 AM
Method	tune_low no focus50-1600da31052021.m	Operator	Dr JHL Jordaan
Sample Name	NH-13	Instrument / Ser#	micrOTOF-Q II 2010390
Comment			

Acquisition Parameter

Source Type	APCI	Ion Polarity	Positive	Set Nebulizer	1.8 Bar
Focus	Not active	Set Capillary	4500 V	Set Dry Heater	200 °C
Scan Begin	50 m/z	Set End Plate Offset	-500 V	Set Dry Gas	8.0 l/min
Scan End	1600 m/z	Set Collision Cell RF	150.0 Vpp	Set Divert Valve	Waste

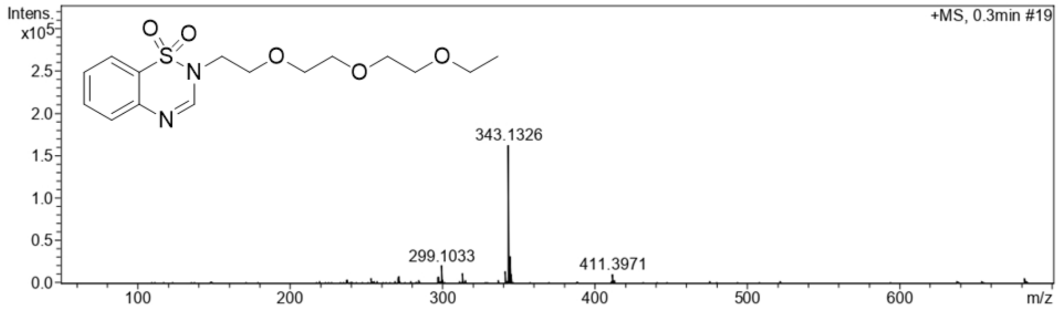

Meas. m/z	#	Formula	Score	m/z	err [mDa]	err [ppm]	mSigma	rdb	e⁻ Conf	N-Rule
343.1326	1	C 15 H 23 N 2 O 5 S	100.00	343.1322	-0.3	-1.0	8.0	5.5	even	ok

HPLC Purity

Analysis Report

SHIMADZU LabSolutions

<Sample Information>

Sample Name : NH-08
Sample ID : NH-08
Data Filename : NH-08_010.lcd
Method Filename : screening.lcm
Batch Filename : purity Sept 2024.lcb
Vial # : 1-9
Injection Volume : 0,2 uL
Date Acquired : 16/09/2024 12:04:44
Date Processed : 16/09/2024 12:49:40

Sample Type : Unknown
Acquired by : System Administrator
Processed by : System Administrator

<Chromatogram>

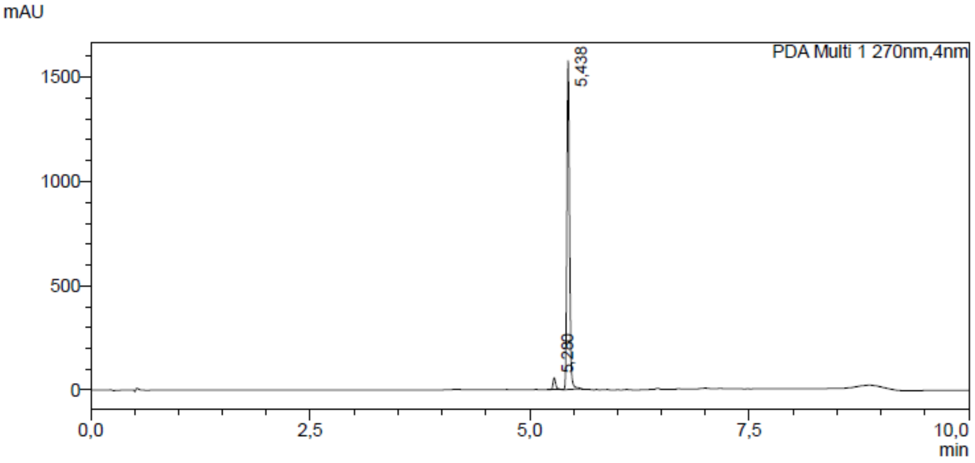

<Peak Table>

PDA Ch1 270nm

Peak#	Ret. Time	Area	Area%
1	5,280	126266	3,732
2	5,438	3256746	96,268
Total		3383012	100,000

2-(2-Phenoxyethyl)-2*H*-benzo[*e*][1,2,4]thiadiazine-1,1-dioxide (9)

^{1}H NMR in DMSO

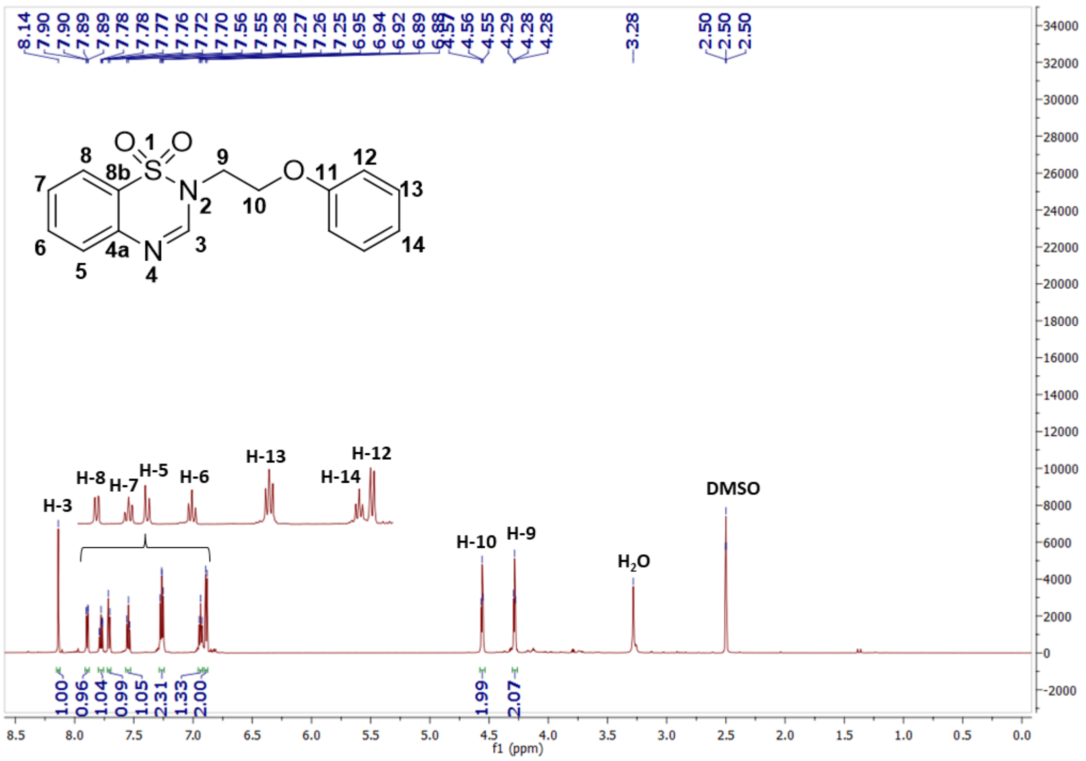

^{13}C NMR in DMSO

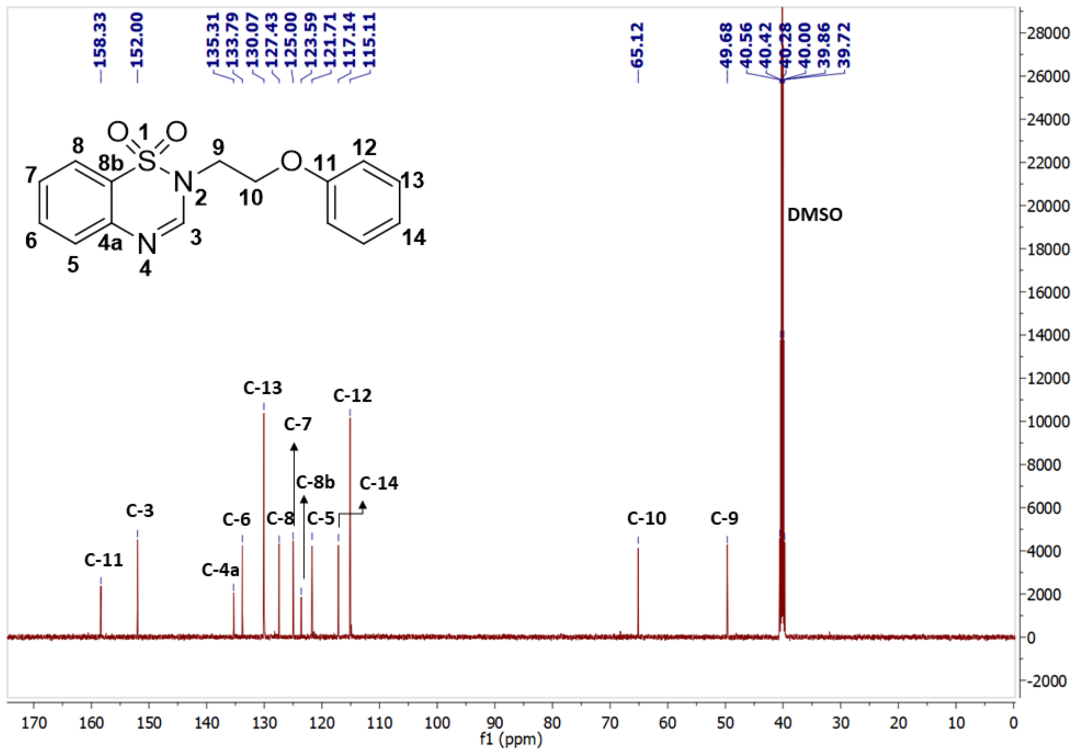

IR Spectrum

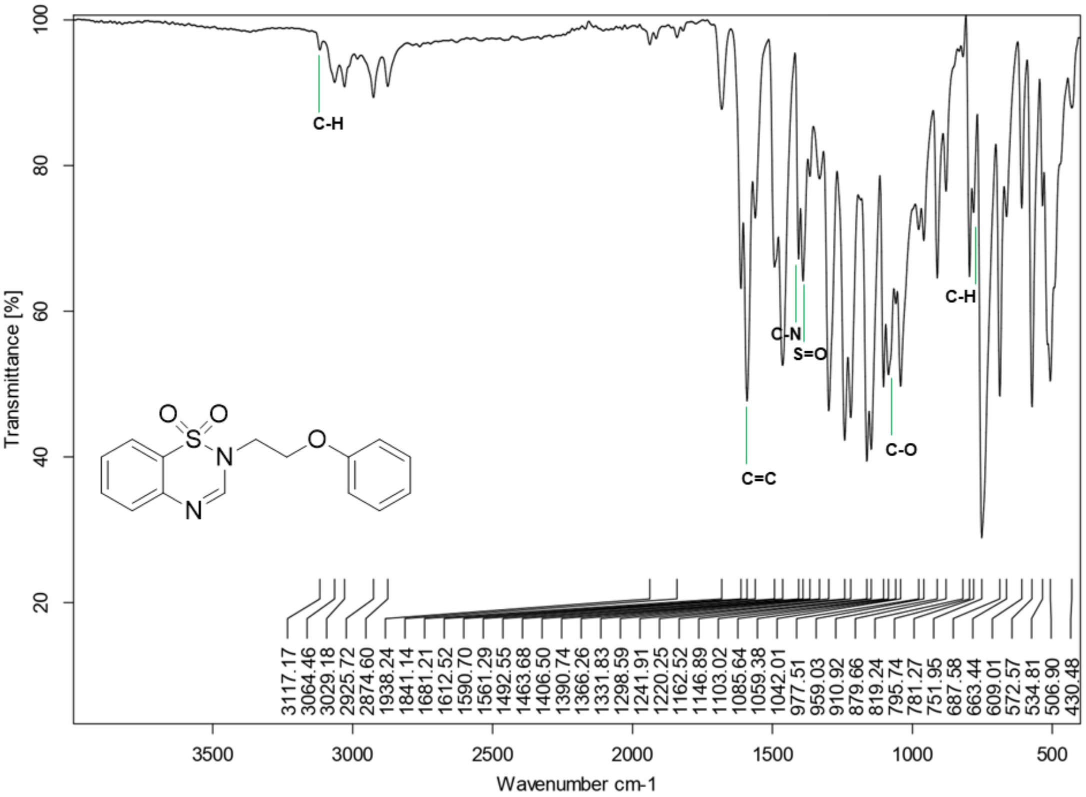

HRMS

Mass Spectrum SmartFormula Report

Analysis Info

Analysis Name	D:\Data\05102021\LADMS000009.d	Acquisition Date	10/5/2021 1:37:45 PM
Method	tune_low no focus50-1600da31052021.m	Operator	Dr JHL Jordaan
Sample Name	NH-5	Instrument / Ser#	micrOTOF-Q II 2010390
Comment			

Acquisition Parameter

Source Type	APCI	Ion Polarity	Positive	Set Nebulizer	1.8 Bar
Focus	Not active	Set Capillary	4500 V	Set Dry Heater	200 °C
Scan Begin	50 m/z	Set End Plate Offset	-500 V	Set Dry Gas	8.0 l/min
Scan End	1600 m/z	Set Collision Cell RF	150.0 Vpp	Set Divert Valve	Waste

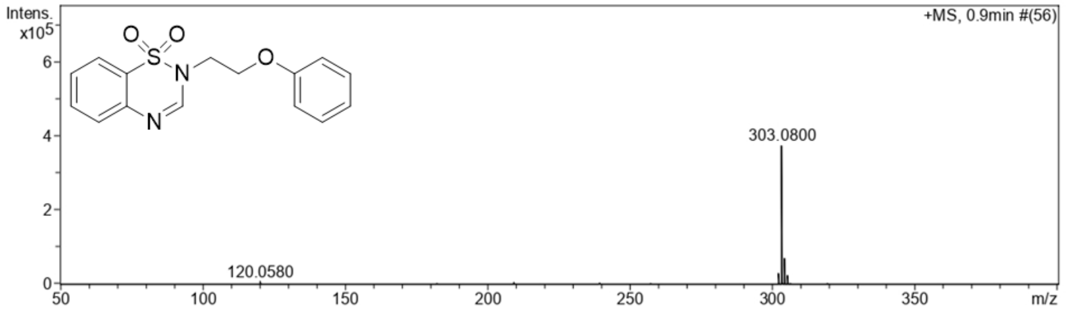

Meas. m/z	#	Formula	Score	m/z	err [mDa]	err [ppm]	mSigma	rdb	e⁻ Conf	N-Rule
303.0800	1	C 15 H 15 N 2 O 3 S	100.00	303.0798	-0.2	-0.6	5.1	9.5	even	ok

HPLC Purity

SHIMADZU LabSolutions

Analysis Report

<Sample Information>

Sample Name	: NH-09 HPLC grade001
Sample ID	: NH-09 HPLC grade001
Data Filename	: NH-09 HPLC grade001_006.lcd
Method Filename	: screening.lcm
Batch Filename	: purity Sept 2024.lcb
Vial #	: 1-17
Injection Volume	: 3 uL
Date Acquired	: 17/09/2024 10:00:43
Date Processed	: 17/09/2024 10:10:44
Sample Type	: Unknown
Acquired by	: System Administrator
Processed by	: System Administrator

<Chromatogram>

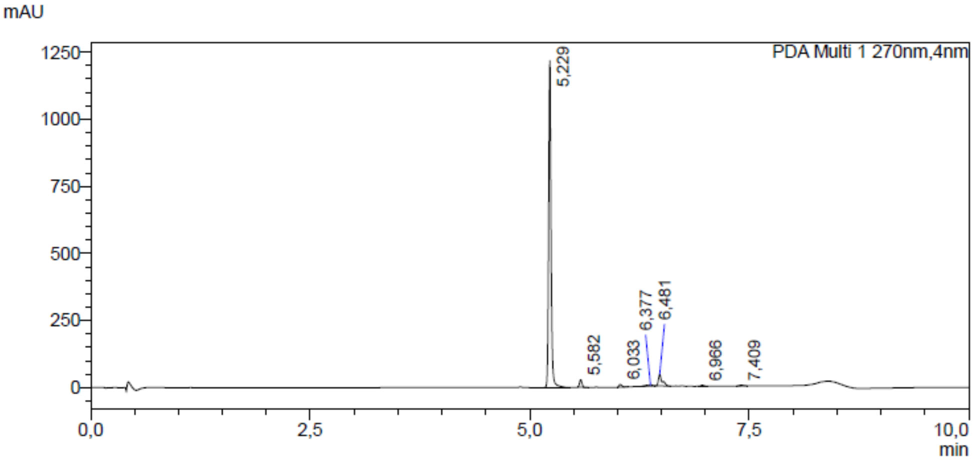

<Peak Table>

PDA Ch1 270nm

Peak#	Ret. Time	Area	Area%
1	5,229	2409992	90,460
2	5,582	53477	2,007
3	6,033	22499	0,845
4	6,377	27885	1,047
5	6,481	131514	4,936
6	6,966	9057	0,340
7	7,409	9723	0,365
Total		2664147	100,000

2-[2-(4-Bromophenoxy)ethyl]-2*H*-benzo[*e*][1,2,4]thiadiazine-1,1-dioxide (10)

^{1}H NMR in DMSO

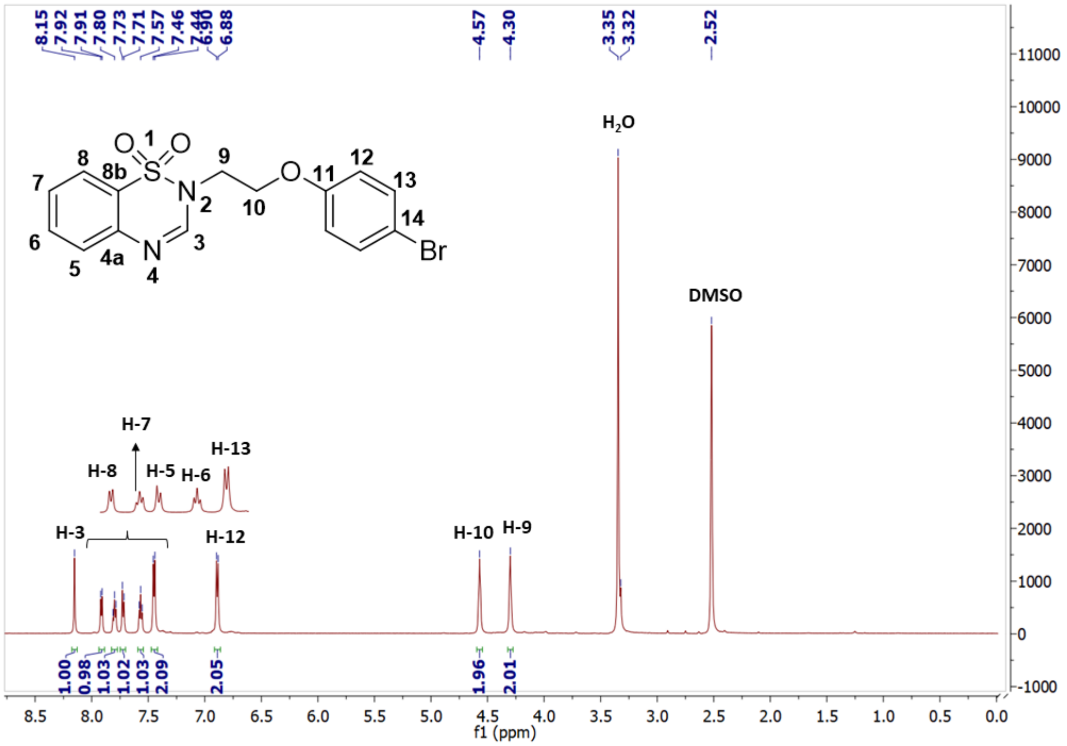

^{13}C NMR in DMSO

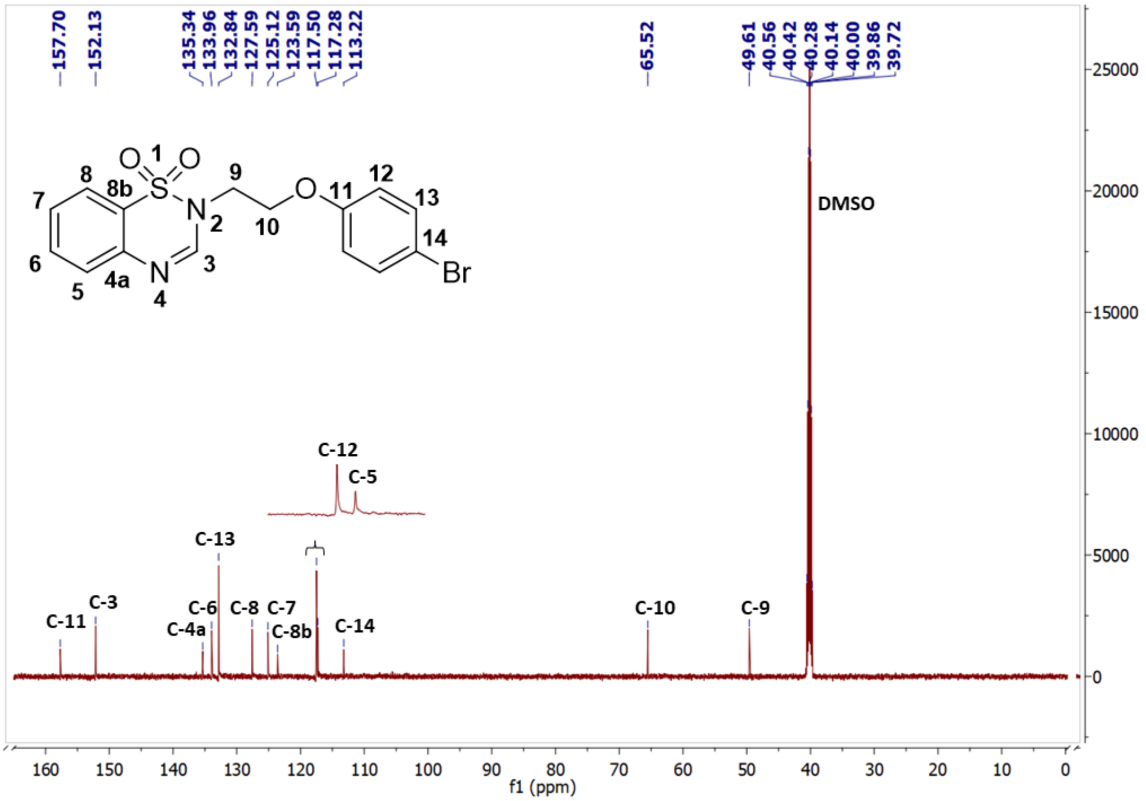

IR Spectrum

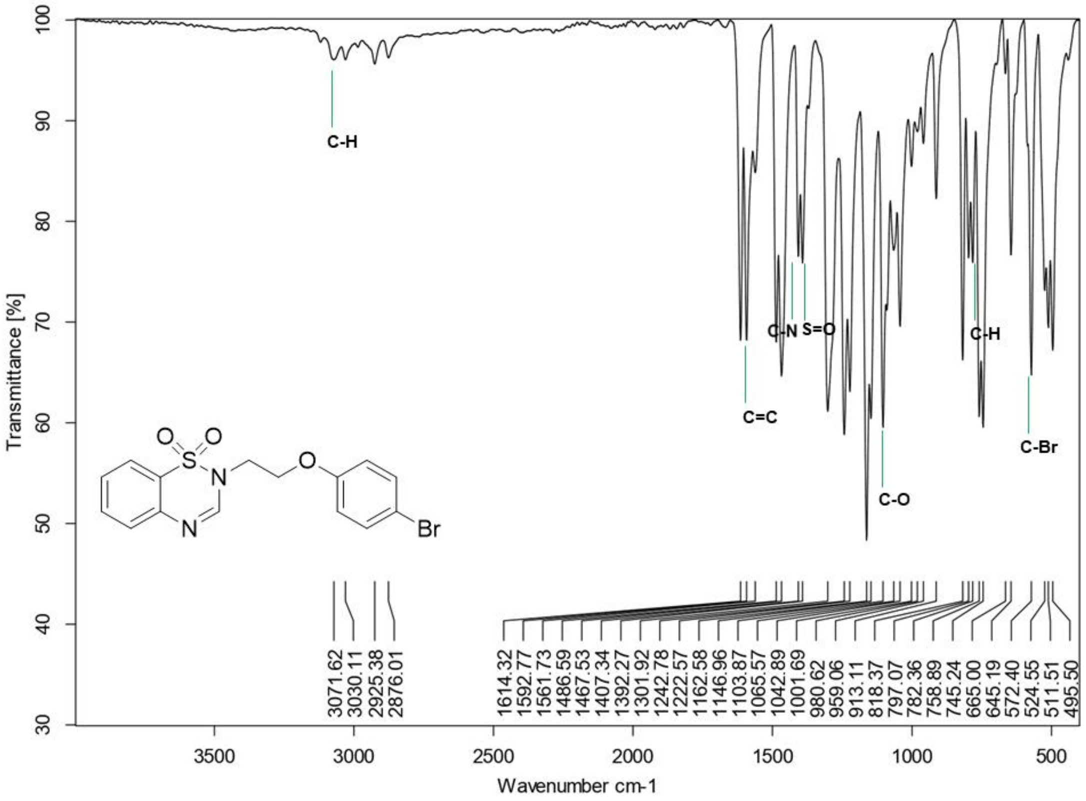

HRMS

Mass Spectrum SmartFormula Report

Analysis Info

Analysis Name	D:\Data\18112021\LADMS000003.d	Acquisition Date	11/18/2021 8:42:22 AM
Method	tune_low no focus50-1600da31052021.m	Operator	Dr JHL Jordaan
Sample Name	NH-12	Instrument / Ser#	micrOTOF-Q II 2010390
Comment			

Acquisition Parameter

Source Type	APCI	Ion Polarity	Positive	Set Nebulizer	1.8 Bar
Focus	Not active	Set Capillary	4500 V	Set Dry Heater	200 °C
Scan Begin	50 m/z	Set End Plate Offset	-500 V	Set Dry Gas	4.0 l/min
Scan End	1600 m/z	Set Collision Cell RF	150.0 Vpp	Set Divert Valve	Waste

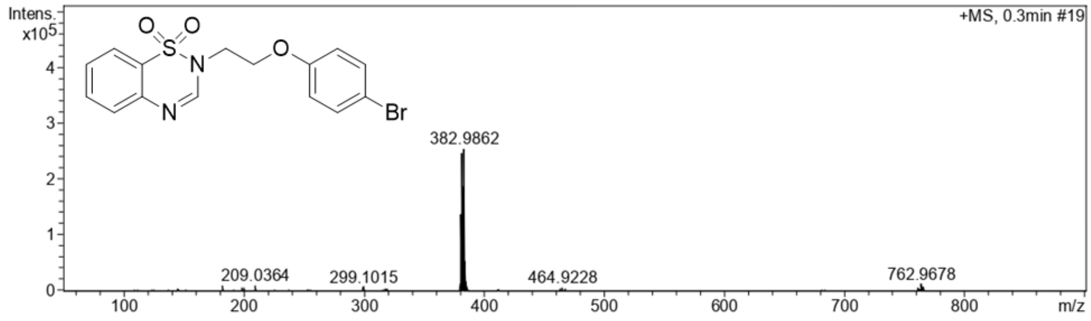

Meas. m/z	#	Formula	Score	m/z	err [mDa]	err [ppm]	mSigma	rdb	e⁻ Conf	N-Rule
380.9884	1	C 15 H 14 Br N 2 O 3 S	100.00	380.9903	1.9	5.0	229.5	9.5	even	ok

HPLC Purity

Analysis Report

SHIMADZU LabSolutions

<Sample Information>

Sample Name	: NH-10
Sample ID	: NH-10
Data Filename	: NH-10_003.lcd
Method Filename	: screening.lcm
Batch Filename	: purity Sept 2024.lcb
Vial #	: 1-11
Injection Volume	: 3 uL
Date Acquired	: 16/09/2024 13:42:48
Date Processed	: 16/09/2024 13:52:49
Sample Type	: Unknown
Acquired by	: System Administrator
Processed by	: System Administrator

<Chromatogram>

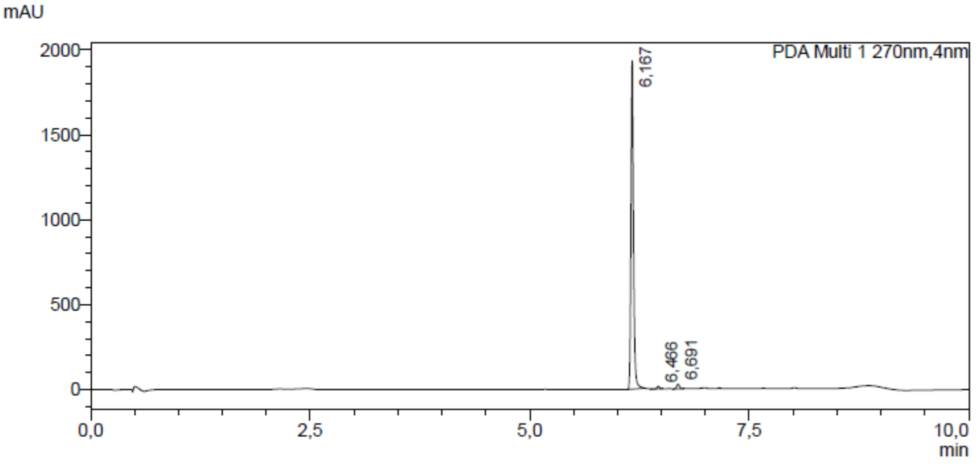

<Peak Table>

PDA Ch1 270nm

Peak#	Ret. Time	Area	Area%
1	6,167	3994275	97,813
2	6,466	27591	0,676
3	6,691	61733	1,512
Total		4083598	100,000

2-[2-(4-Chlorophenoxy)ethyl]-2*H*-benzo[*e*][1,2,4]thiadiazine-1,1-dioxide (11)

^{1}H NMR in DMSO

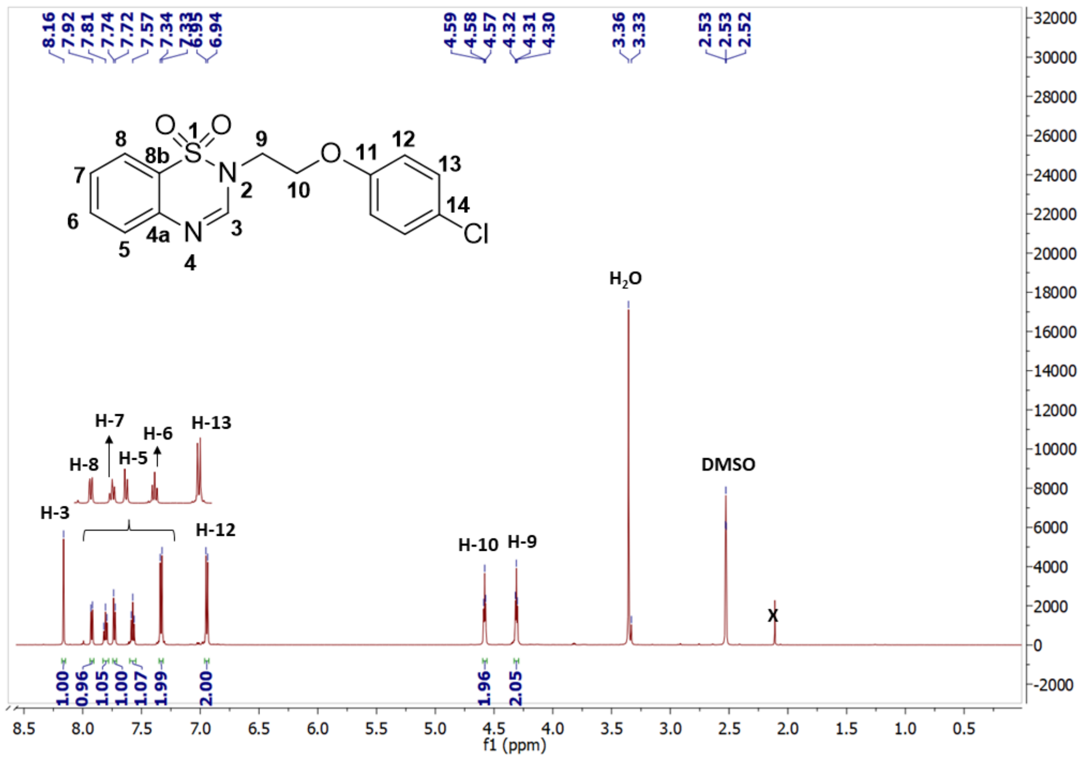

^{13}C NMR in DMSO

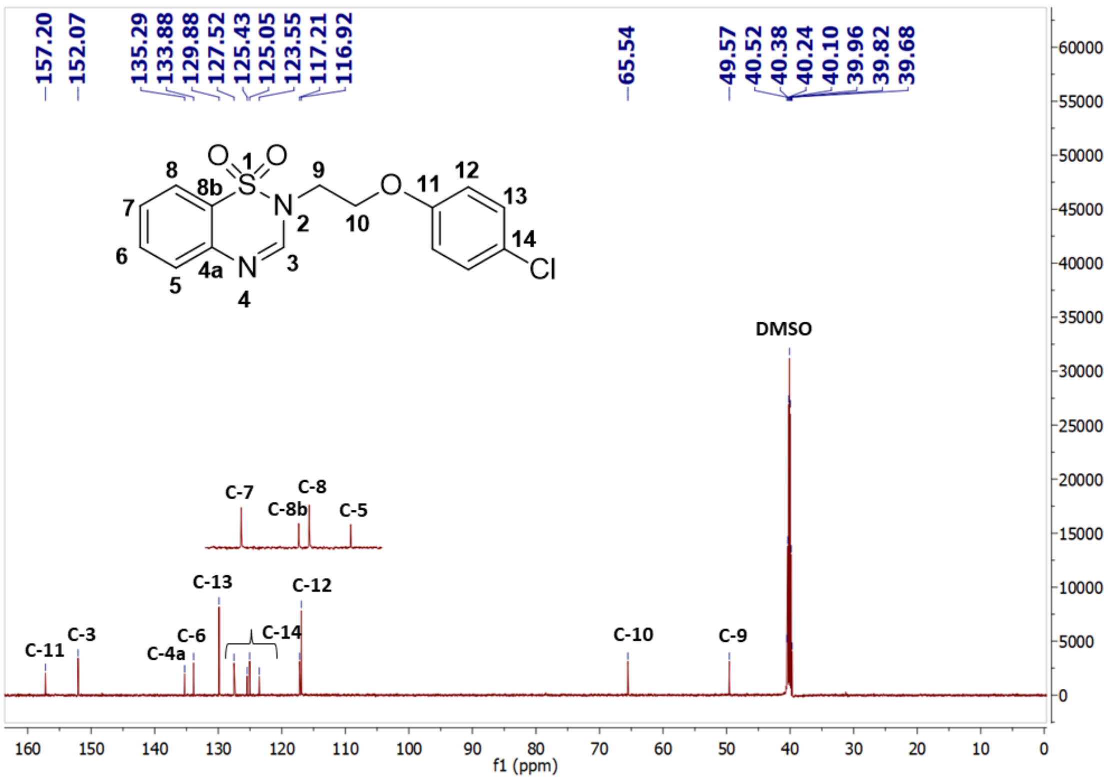

IR Spectrum

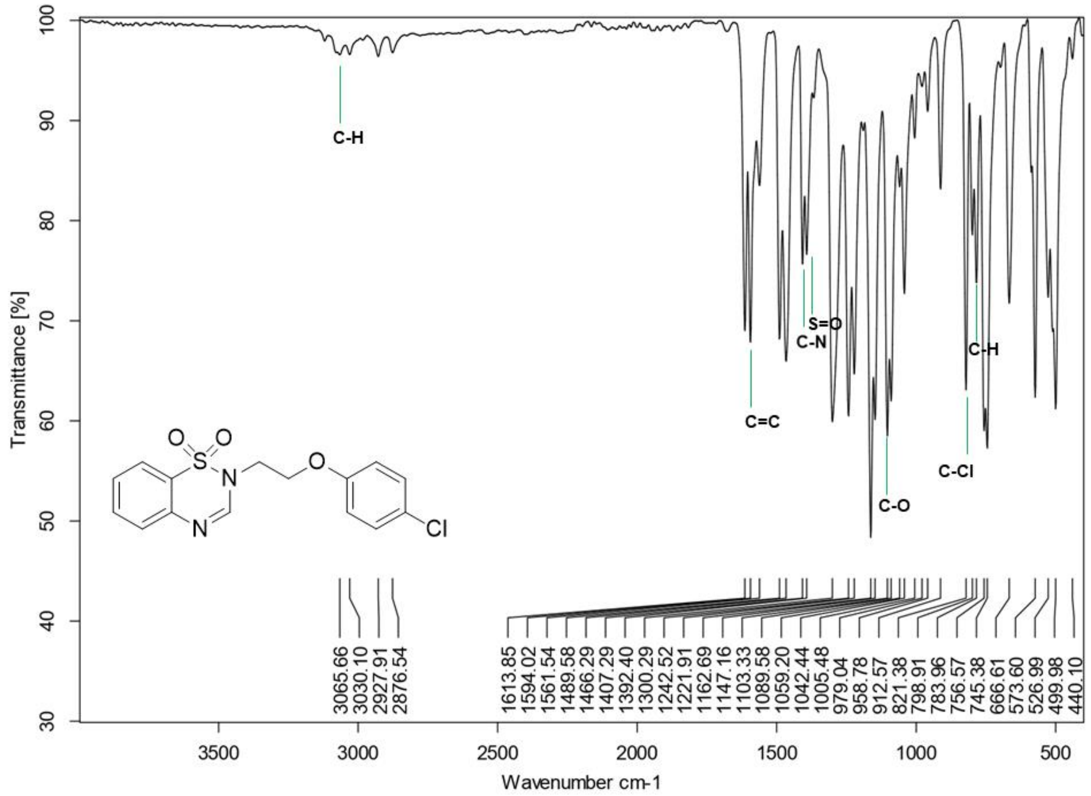

HRMS

Mass Spectrum SmartFormula Report

Analysis Info

Analysis Name	D:\Data\18112021\LADMS000007.d	Acquisition Date	11/18/2021 9:01:39 AM
Method	tune_low no focus50-1600da31052021.m	Operator	Dr JHL Jordaan
Sample Name	NH-14	Instrument / Ser#	micrOTOF-Q II 2010390
Comment			

Acquisition Parameter

Source Type	APCI	Ion Polarity	Positive	Set Nebulizer	1.8 Bar
Focus	Not active	Set Capillary	4500 V	Set Dry Heater	200 °C
Scan Begin	50 m/z	Set End Plate Offset	-500 V	Set Dry Gas	8.0 l/min
Scan End	1600 m/z	Set Collision Cell RF	150.0 Vpp	Set Divert Valve	Waste

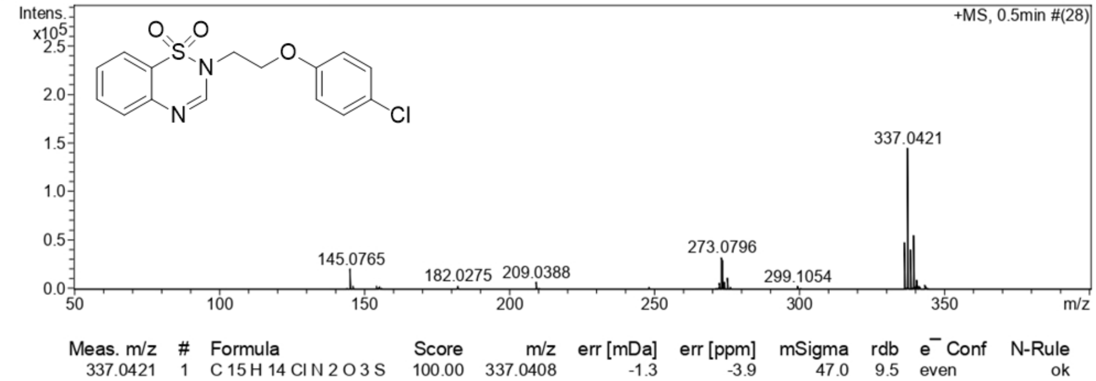

Meas. m/z	#	Formula	Score	m/z	err [mDa]	err [ppm]	mSigma	rdb	e⁻ Conf	N-Rule
337.0421	1	C 15 H 14 Cl N 2 O 3 S	100.00	337.0408	-1.3	-3.9	47.0	9.5	even	ok

HPLC Purity

Analysis Report

SHIMADZU LabSolutions

<Sample Information>

Sample Name : NH-11
Sample ID : NH-11
Data Filename : NH-11_004.lcd
Method Filename : screening.lcm
Batch Filename : purity Sept 2024.lcb
Vial # : 1-12
Injection Volume : 1 uL
Date Acquired : 16/09/2024 13:53:10
Date Processed : 16/09/2024 14:03:11

Sample Type : Unknown
Acquired by : System Administrator
Processed by : System Administrator

<Chromatogram>

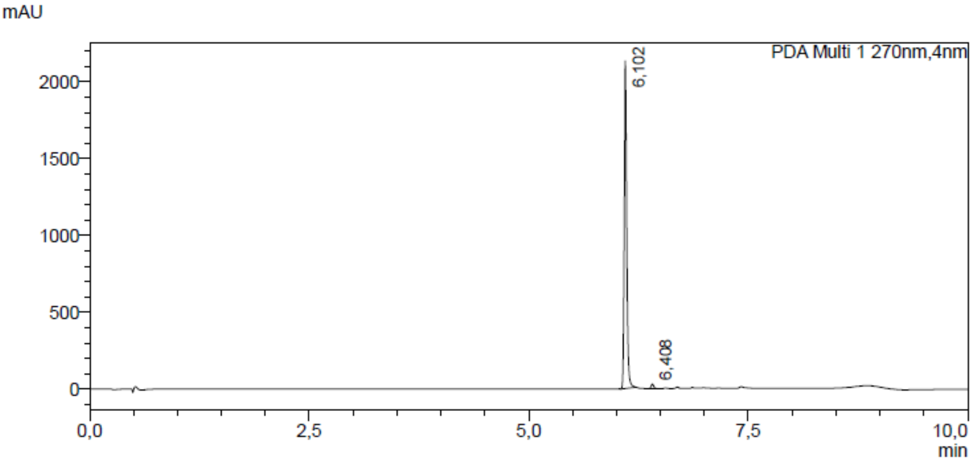

<Peak Table>

PDA Ch1 270nm

Peak#	Ret. Time	Area	Area%
1	6,102	4273371	98,540
2	6,408	63308	1,460
Total		4336678	100,000

2-[2-(4-Nitrophenoxy)ethyl]-2*H*-benzo[*e*][1,2,4]thiadiazine-1,1-dioxide (12)

^{1}H NMR in DMSO

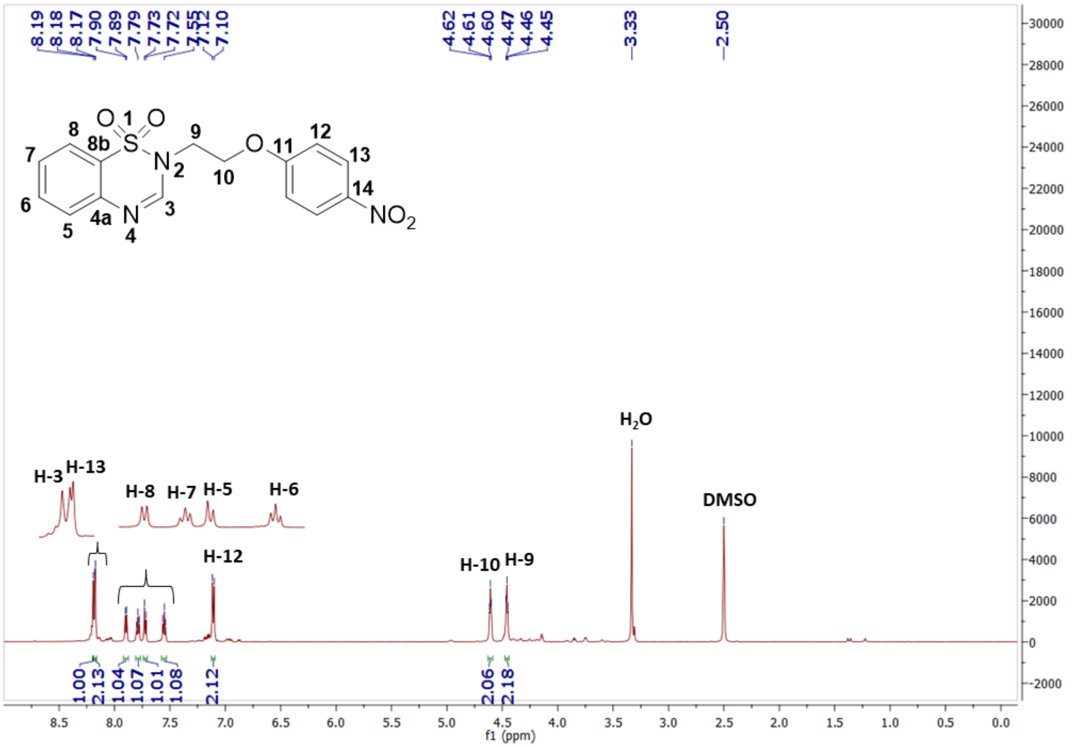

^{13}C NMR in DMSO

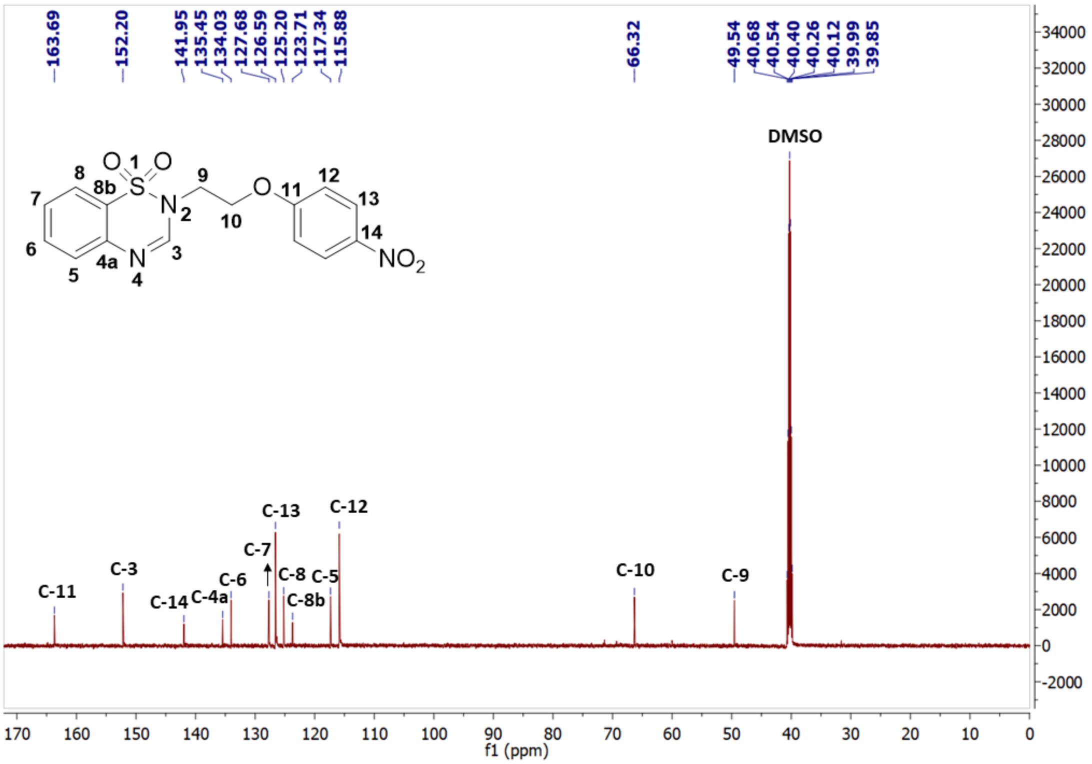

IR Spectrum

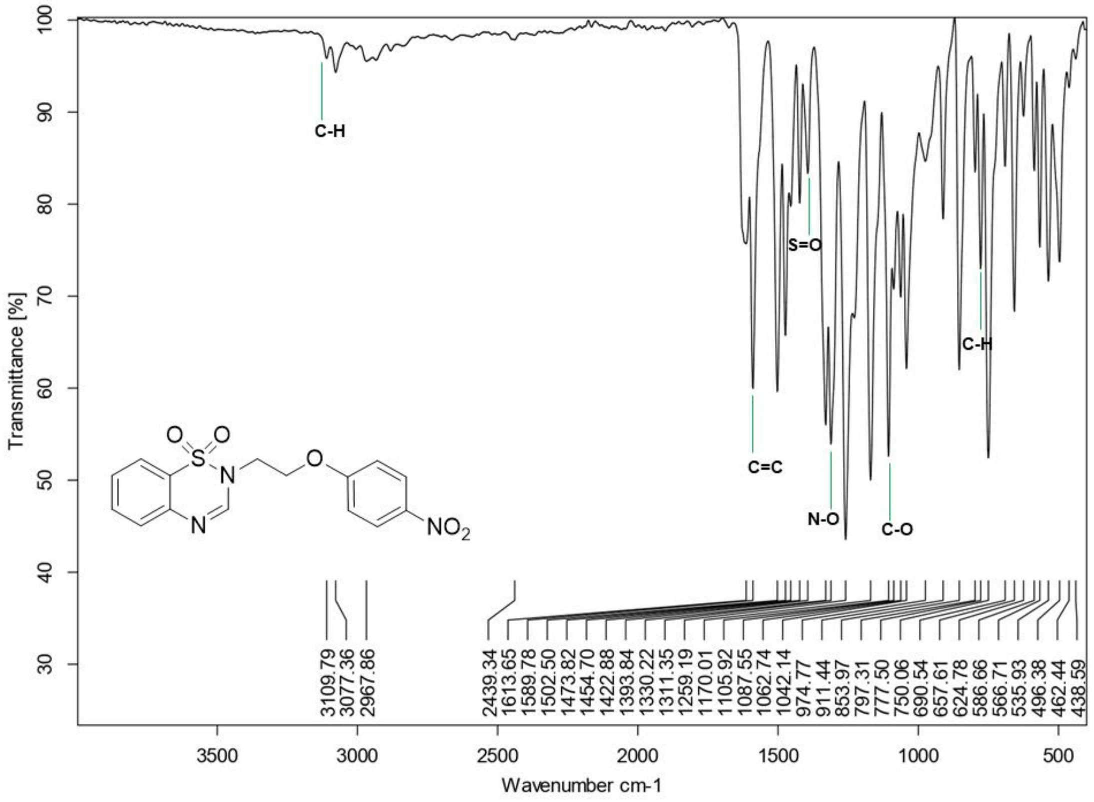

HRMS

Mass Spectrum SmartFormula Report

Analysis Info

Analysis Name	D:\Data\05102021\LADMS000014.d	Acquisition Date	10/5/2021 2:04:04 PM
Method	tune_low no focus50-1600da31052021.m	Operator	Dr JHL Jordaan
Sample Name	NH-10	Instrument / Ser#	micrOTOF-Q II 2010390
Comment			

Acquisition Parameter

Source Type	APCI	Ion Polarity	Positive	Set Nebulizer	1.8 Bar
Focus	Not active	Set Capillary	4500 V	Set Dry Heater	200 °C
Scan Begin	50 m/z	Set End Plate Offset	-500 V	Set Dry Gas	8.0 l/min
Scan End	1600 m/z	Set Collision Cell RF	150.0 Vpp	Set Divert Valve	Waste

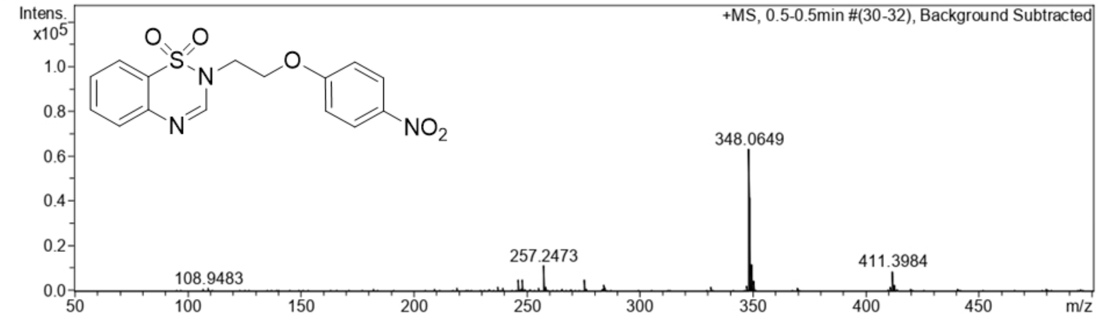

Meas. m/z	#	Formula	Score	m/z	err [mDa]	err [ppm]	mSigma	rdb	e⁻ Conf	N-Rule
348.0649	1	C 15 H 14 N 3 O 5 S	100.00	348.0649	-0.0	-0.0	4.4	10.5	even	ok

HPLC Purity

Analysis Report

SHIMADZU LabSolutions

<Sample Information>

Sample Name : NH-12
Sample ID : NH-12
Data Filename : NH-12_005.lcd
Method Filename : screening.lcm
Batch Filename : purity Sept 2024.lcb
Vial # : 1-13
Injection Volume : 1 uL
Date Acquired : 16/09/2024 14:03:31
Date Processed : 16/09/2024 14:13:32

Sample Type : Unknown

Acquired by : System Administrator
Processed by : System Administrator

<Chromatogram>

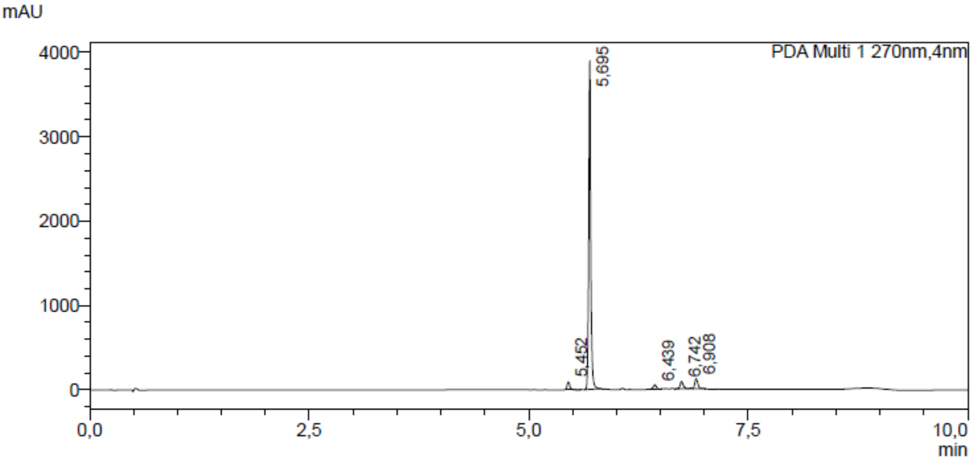

<Peak Table>

PDA Ch1 270nm

Peak#	Ret. Time	Area	Area%
1	5,452	170079	2,350
2	5,695	6465636	89,348
3	6,439	119276	1,648
4	6,742	207209	2,863
5	6,908	274300	3,791
Total		7236498	100,000

2-[2-(Allyloxy)ethyl]-2*H*-benzo[*e*][1,2,4]thiadiazine-1,1-dioxide (13)

^{1}H NMR in DMSO

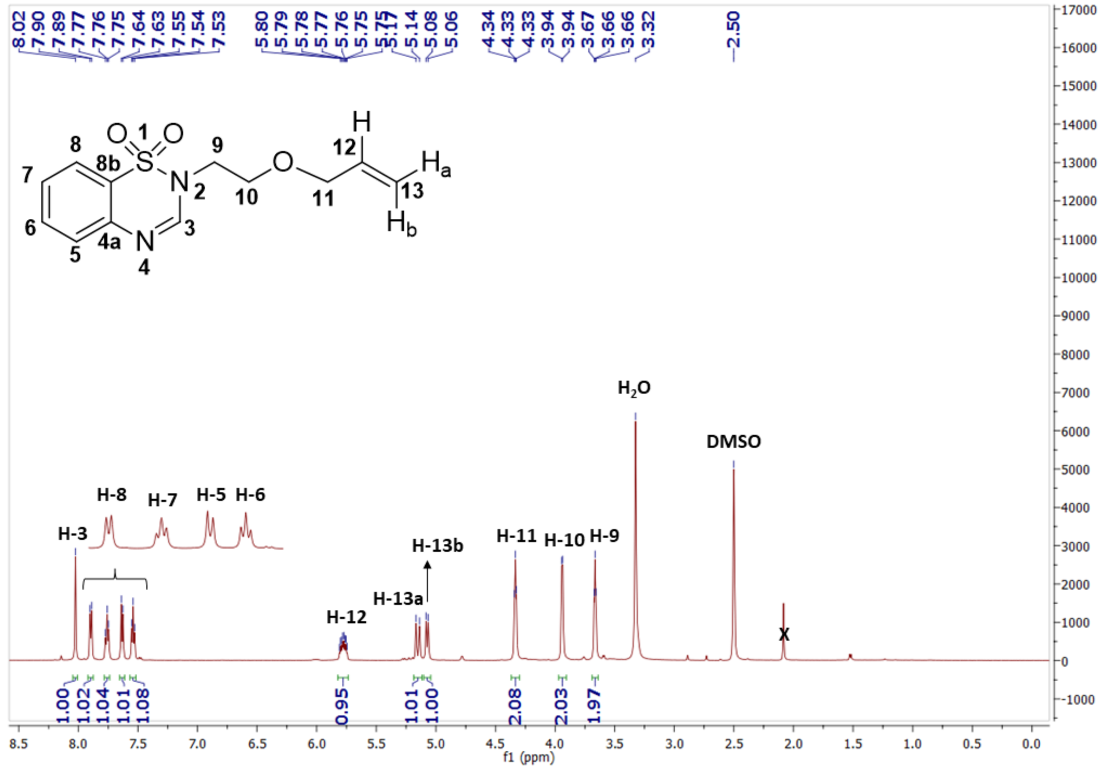

^{13}C NMR in DMSO

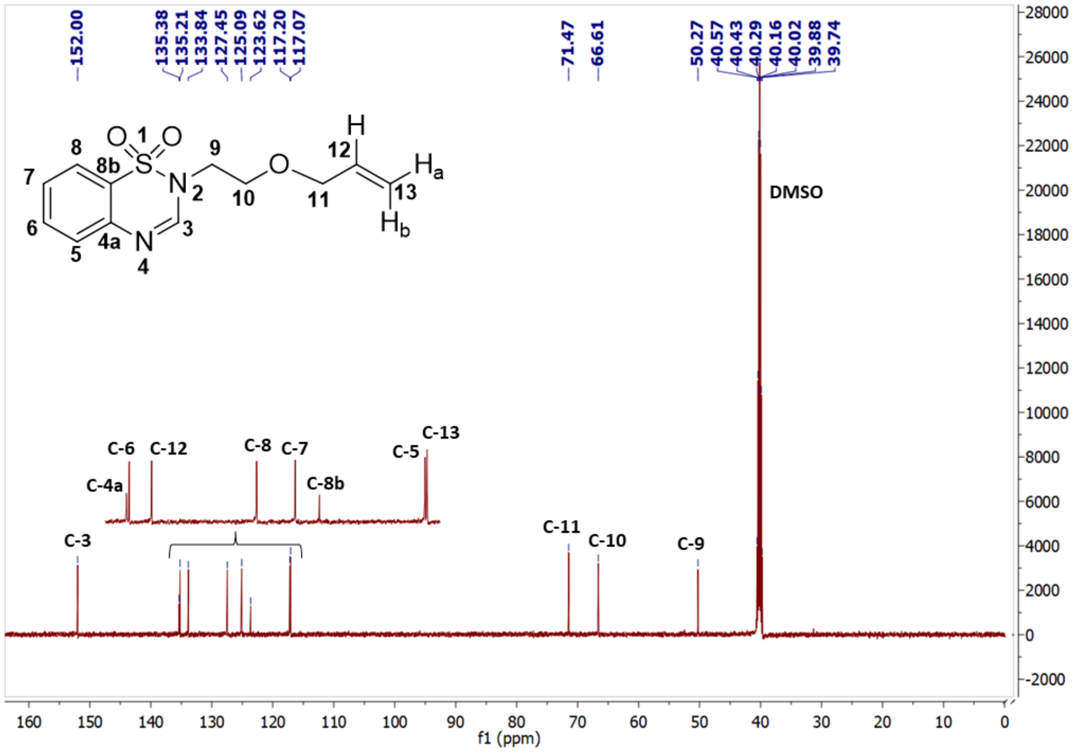

IR Spectrum

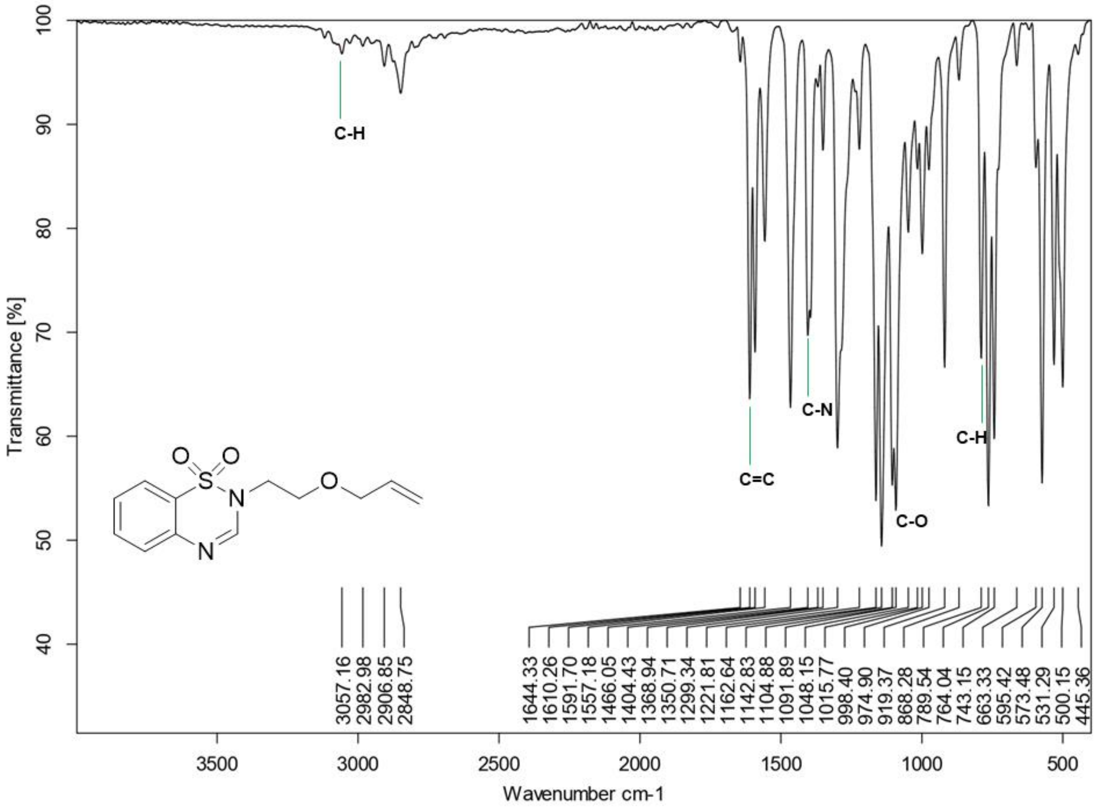

HRMS

Mass Spectrum SmartFormula Report

Analysis Info

Analysis Name	D:\Data\05102021\LADMS000012.d	Acquisition Date	10/5/2021 1:51:22 PM
Method	tune_low no focus50-1600da31052021.m	Operator	Dr JHL Jordaan
Sample Name	NH-8	Instrument / Ser#	micrOTOF-Q II 2010390
Comment			

Acquisition Parameter

Source Type	APCI	Ion Polarity	Positive	Set Nebulizer	1.8 Bar
Focus	Not active	Set Capillary	4500 V	Set Dry Heater	200 °C
Scan Begin	50 m/z	Set End Plate Offset	-500 V	Set Dry Gas	8.0 l/min
Scan End	1600 m/z	Set Collision Cell RF	150.0 Vpp	Set Divert Valve	Waste

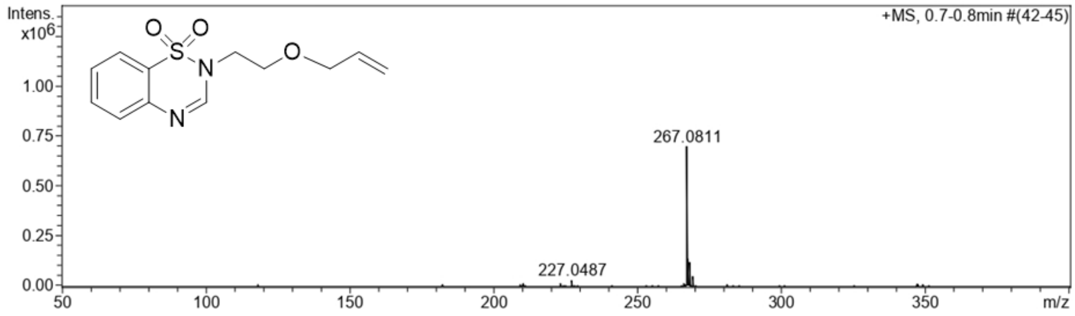

Meas. m/z	#	Formula	Score	m/z	err [mDa]	err [ppm]	mSigma	rdb	e⁻ Conf	N-Rule
267.0811	1	C 12 H 15 N 2 O 3 S	100.00	267.0798	-1.3	-4.9	13.4	6.5	even	ok

HPLC Purity

Analysis Report

SHIMADZU LabSolutions

<Sample Information>

Sample Name : NH-013
Sample ID : NH-013
Data Filename : NH-013_006.lcd
Method Filename : screening.lcm
Batch Filename : purity Sept 2024.lcb
Vial # : 1-14
Injection Volume : 1 uL
Date Acquired : 16/09/2024 14:13:54
Date Processed : 16/09/2024 14:23:55

Sample Type : Unknown
Acquired by : System Administrator
Processed by : System Administrator

<Chromatogram>

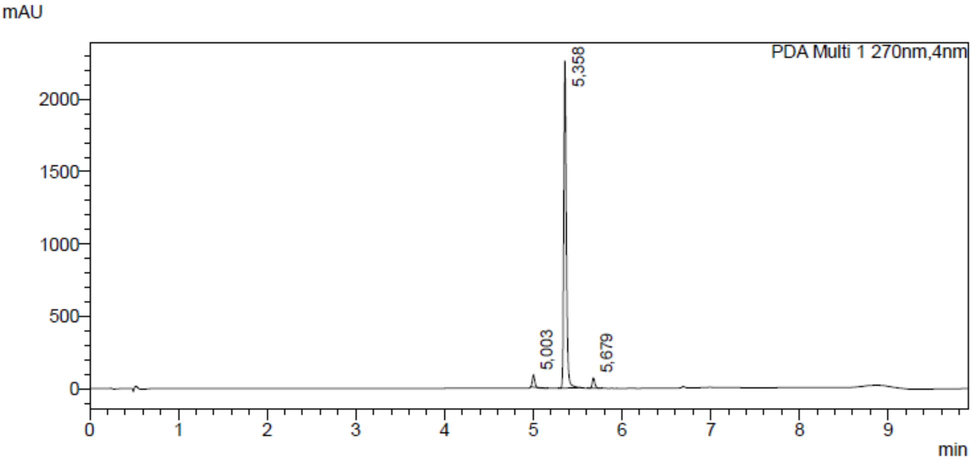

<Peak Table>

PDA Ch1 270nm

Peak#	Ret. Time	Area	Area%
1	5,003	142715	3,006
2	5,358	4455758	93,841
3	5,679	149739	3,154
Total		4748211	100,000

2-(Prop-2-yn-1-yl)-2*H*-benzo[*e*][1,2,4]thiadiazine-1,1-dioxide (14)

^{1}H NMR in DMSO

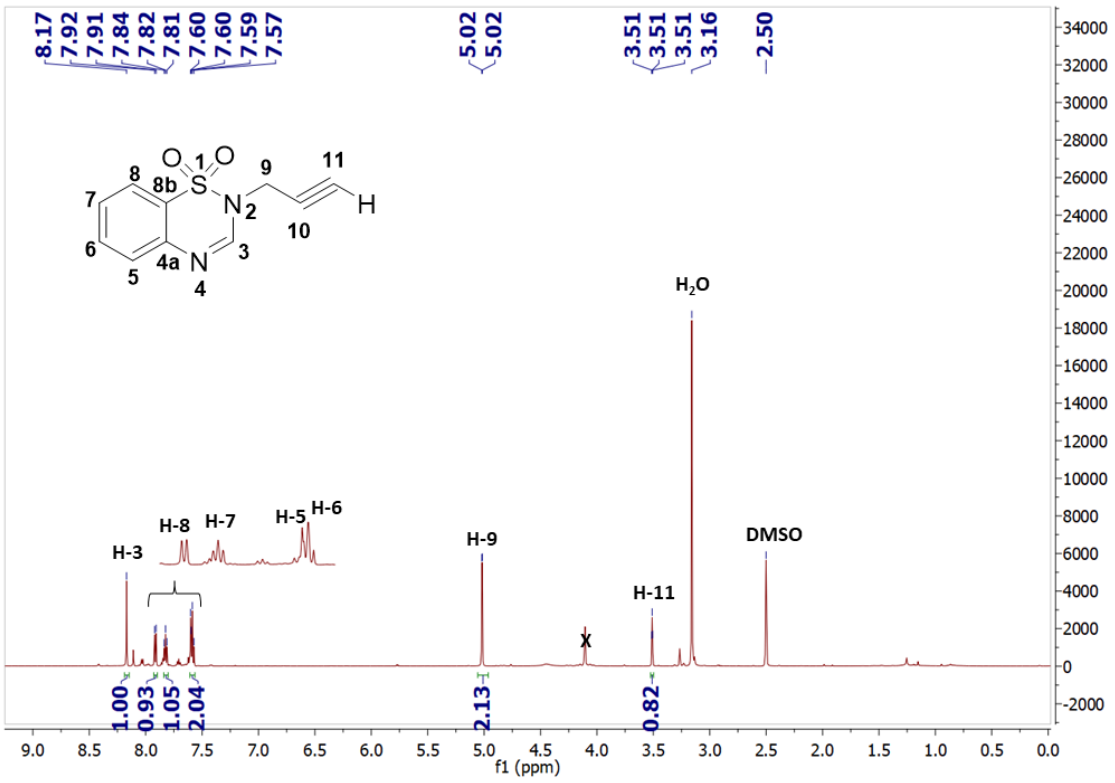

^{13}C NMR in DMSO

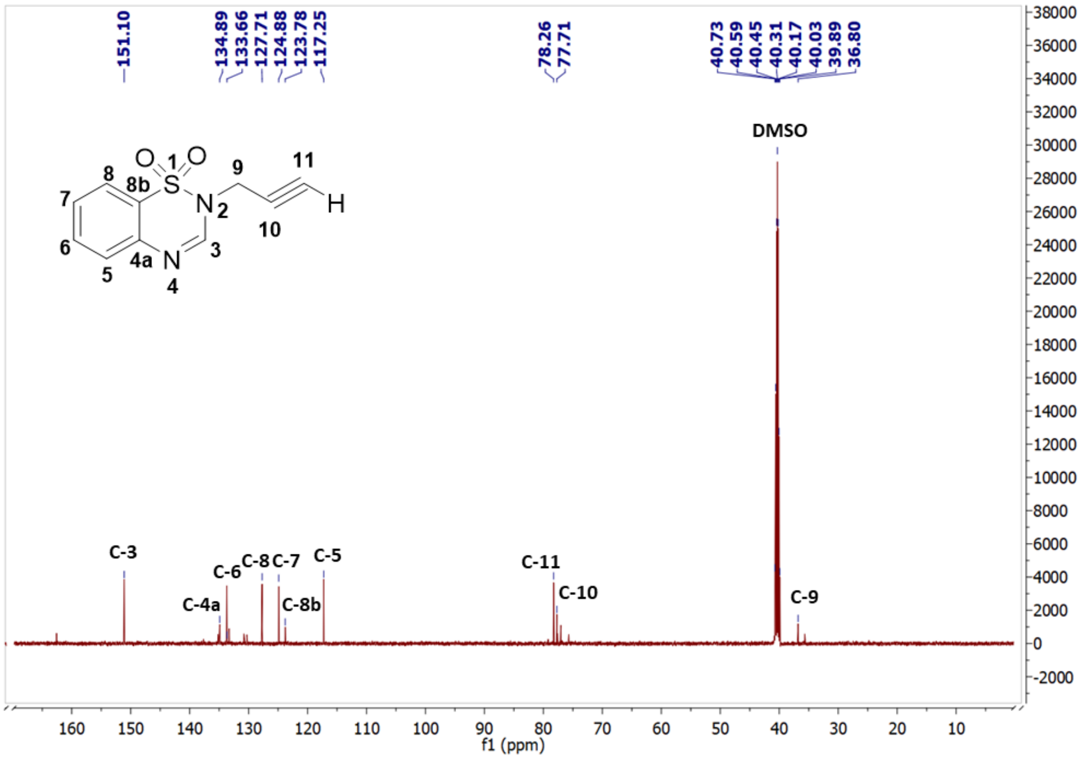

IR Spectrum

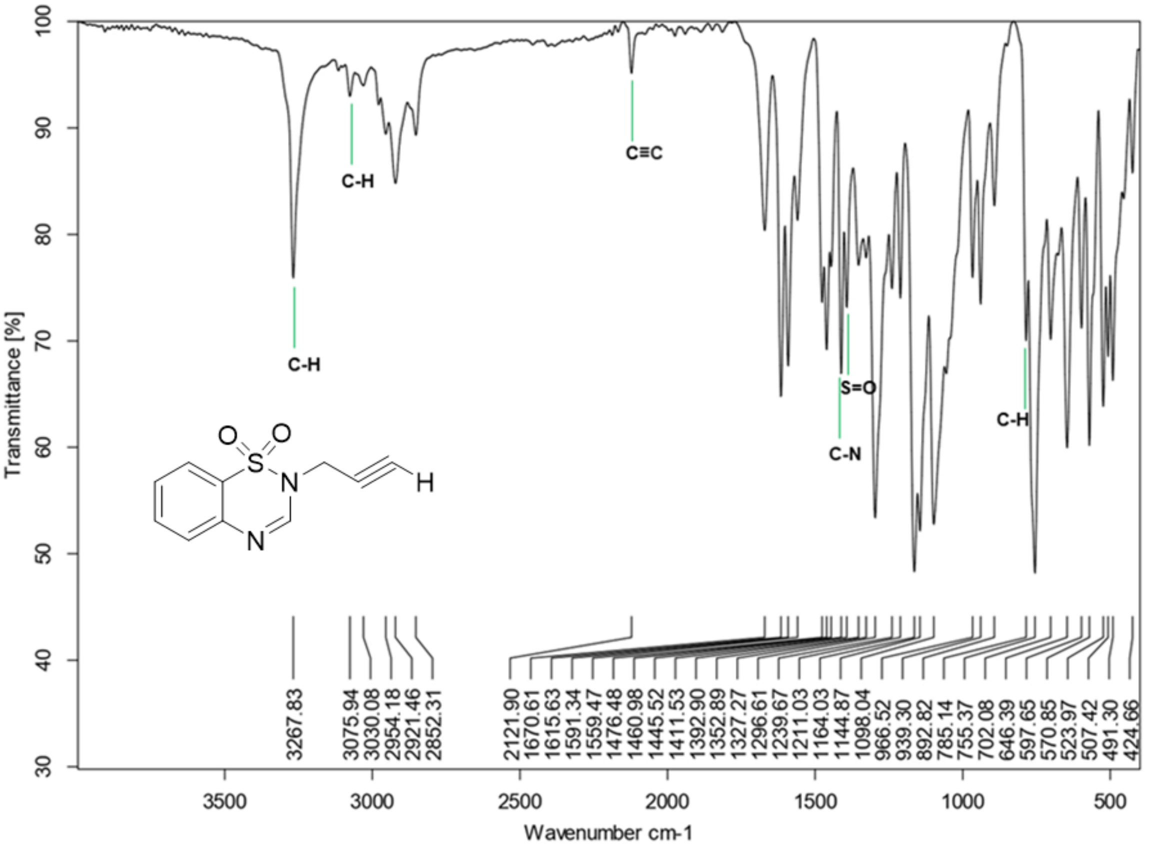

HRMS

Mass Spectrum SmartFormula Report

Analysis Info

Analysis Name	D:\Data\11022022\LADMS000005.d	Acquisition Date	2/11/2022 10:38:38 AM
Method	tune_low no focus50-1600da27012022HPLC.m	Operator	Dr JHL Jordaan
Sample Name	NH-18	Instrument / Ser#	micrOTOF-Q II 2010390
Comment			

Acquisition Parameter

Source Type	APCI	Ion Polarity	Positive	Set Nebulizer	1.8 Bar
Focus	Not active	Set Capillary	4500 V	Set Dry Heater	200 °C
Scan Begin	50 m/z	Set End Plate Offset	-500 V	Set Dry Gas	4.0 l/min
Scan End	1600 m/z	Set Collision Cell RF	150.0 Vpp	Set Divert Valve	Waste

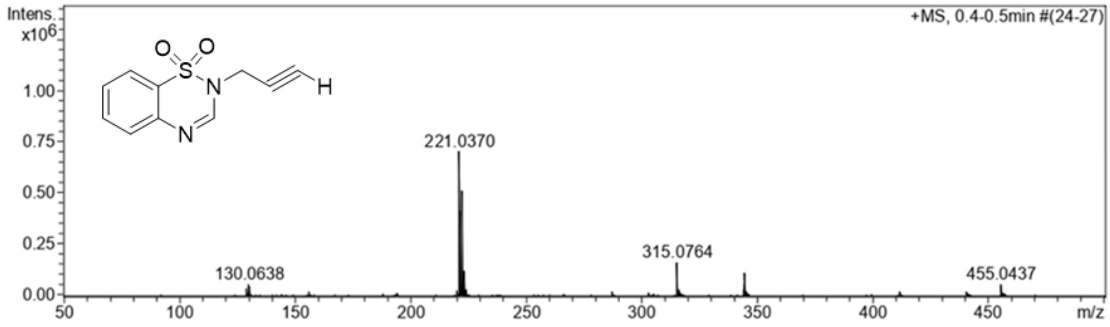

Meas. m/z	#	Formula	Score	m/z	err [mDa]	err [ppm]	mSigma	rdb	e¯ Conf	N-Rule
221.0370	1	C 10 H 9 N 2 O 2 S	100.00	221.0379	1.0	4.3	308.6	7.5	even	ok

HPLC Purity

Analysis Report

SHIMADZU LabSolutions

<Sample Information>

Sample Name : NH-014
Sample ID : NH-014
Data Filename : NH-014_016.lcd
Method Filename : screening.lcm
Batch Filename : purity Sept 2024.lcb
Vial # : 1-15
Injection Volume : 0,2 uL
Date Acquired : 16/09/2024 13:07:13
Date Processed : 16/09/2024 13:17:15

Sample Type : Unknown
Acquired by : System Administrator
Processed by : System Administrator

<Chromatogram>

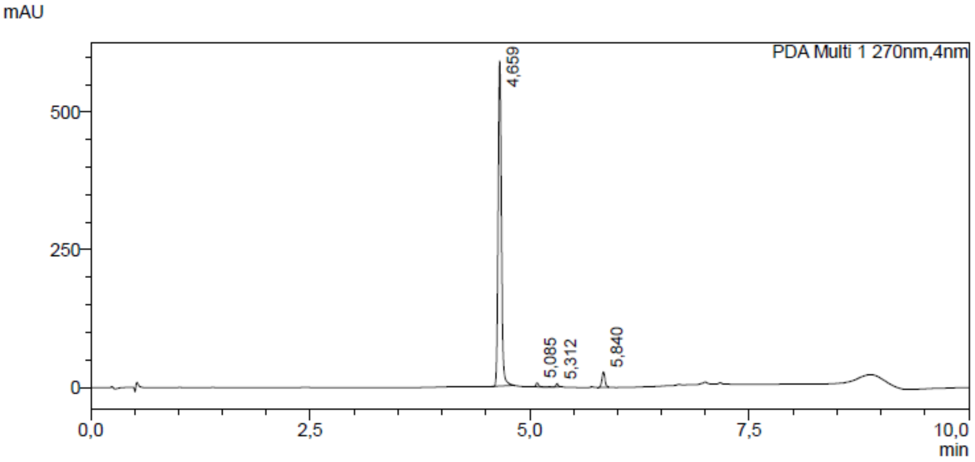

<Peak Table>

PDA Ch1 270nm

Peak#	Ret. Time	Area	Area%
1	4,659	1505872	94,232
2	5,085	15311	0,958
3	5,312	10261	0,642
4	5,840	66605	4,168
Total		1598050	100,000
